# Yi-Shen-Hua-Shi granules inhibit diabetic nephropathy by ameliorating podocyte injury induced by macrophage-derived exosomes

**DOI:** 10.3389/fphar.2022.962606

**Published:** 2022-11-25

**Authors:** Mingzhu Liang, Xiaodong Zhu, Di Zhang, Wenfang He, Jinshi Zhang, Shizhu Yuan, Qiang He, Juan Jin

**Affiliations:** ^1^ Urology and Nephrology Center, Department of Nephrology, Zhejiang Provincial People’s Hospital, Affiliated People’s Hospital, Hangzhou Medical College, Hangzhou, China; ^2^ Department of Nephrology, The Medical College of Qingdao University, Qingdao, China; ^3^ Department of Nephrology, The First Affiliated Hospital of Zhejiang Chinese Medical University (Zhejiang Provincial Hospital of Traditional Chinese Medicine), Hangzhou, China

**Keywords:** Yi-Shen-Hua-Shi granule, diabetic nephropathy, podocyte injury, macrophage-derived exosomes, miR-21a-5p

## Abstract

**Objective:** To observe the therapeutic effect of Yi-Shen-Hua-Shi (YSHS) granule in podocyte damage and diabetic nephropathy (DN) proteinuria and to explore the corresponding mechanism.

**Methods:** The db/db mice were used to establish the DN model. Serum creatinine (SCr), blood urea nitrogen (BUN), and 24 h urinary proteinuria were detected with specific kits. Glomerular structural lesions and podocyte apoptosis were detected through HE staining, TUNEL assay, and immunofluorescence. The medicated serum of YSHS granule (YSHS-serum) or control serum was prepared. Macrophage-derived exosomes were extracted using an exosome extraction kit. Morphology and the protein concentration of exosomes were evaluated by a transmission electron microscope (TEM) and BCA kit. The activity and apoptosis of podocyte MPC5 cells, the M1 macrophage polarization, and the protein expression of an exosome marker and cleaved caspase were detected by the CCK8 experiment, flow cytometry, and Western blot, respectively. The miR-21a-5p expression in podocytes and the exosomes from macrophages were measured by qRT-PCR. The effect of YSHS granule on the infiltration of M1 macrophages in the kidney tissue in db/db mice was measured by immunofluorescence.

**Results:** The YSHS granule could improve renal function, reduce proteinuria, and inhibit glomerular structural lesions and podocyte apoptosis in db/db mice. High-glucose (HG) stimulation and YSHS granule treatment did not affect the protein concentration in macrophage-derived exosomes. Macrophage-derived exosomes could inhibit the cell viability and increase apoptosis of podocytes, especially the exosomes from macrophages treated with HG and control serum. Compared with the exosomes secreted by macrophages after an HG treatment, the exosome from macrophages treated with HG and YSHS granule showed lower inhibitory effects on podocyte activity, accompanied by the decreased upregulating effects of macrophage-derived exosomes on the miR-21a-5p in podocytes. miR-21a-5p mimics could reduce podocyte activity and promote caspase-3 shearing. M1 polarization of macrophages could change the content of miR-21a-5p in macrophage-derived exosomes. In addition, YSHS granule could inhibit HG-induced M1 polarization of macrophages and M1 macrophage infiltration in renal tissues.

**Conclusion:** The YSHS granule could improve the podocyte injury induced by macrophage-derived exosomes and alleviate the progression of DN. This regulation might be related to the inhibition of M1 macrophage polarization by YSHS granule and the reduction of the miR-21a-5p content in macrophage-derived exosomes.

## 1 Introduction

Diabetic nephropathy (DN), the most common and severe complication of diabetes, has been identified as the main pathogenic factor in the development of chronic kidney diseases and terminal renal failure. About 30–40% of patients with DN in the world gradually progress to end-stage renal disease (ESRD) (Maezawa et al., 2015). The main manifestations of DN are a continuous increase of proteinuria, thickening of glomerular basement membrane (GBM), accumulation of extracellular matrix, promotion of podocyte injury, and progressive renal insufficiency (Zhang et al., 2013). Once proteinuria is not effectively controlled, renal function will rapidly deteriorate and rapidly progress to ESRD. Therefore, it is necessary to search for novel and effective therapeutic approaches to reverse the progression of DN. Podocyte cells (podocytes), as terminally differentiated cells, are the crucial component of the glomerular filtration barrier (Li et al., 2020), and podocyte damage is closely related to proteinuria. The decrease in the number of podocytes and the abnormality of their functional morphology play key roles in the occurrence and development of DN (Zhai et al., 2019). Podocyte apoptosis increases in DN, and the inhibition of podocyte apoptosis could reserve proteinuria in DN (Gong et al., 2019; Jin et al., 2019; Yuan et al., 2021). Decreasing podocyte apoptosis is an important therapeutic strategy for reducing or even reversing DN.

Traditional Chinese medicine (TCM) has been widely applied in clinical treatments (China, Japan, Korea, and other East Asian countries) for 3,000 years. Studies have confirmed that TCM has important therapeutic potentials in DN control. The YSHS granule was derived from the TCM formula of Sheng-Yang-Yi-Wei decoction, which was first recorded in the Nei-Wai-Shang-Bian-Huo-Lun the classic of TCM published in 1247 AD. It is a proprietary TCM composed of 16 herbs, including *Ginseng Radix et Rhizoma (GRR), Astragali Radix (ASR), Atractylodis Macrocephalae Rhizoma (AMR), Poria (POR), Alismatis Rhizoma (ALR), Pinelliae Rhizoma Praeparatum Cum Alumine (PRP), Notopterygii Rhizoma et Radix (NRR), Angelicae Pubescentis Radix (APR), Saposhnikoviae Radix (SAR), Bupleuri Radix (BUR), Coptidis Rhizoma (COR), Paeoniae Radix Alba (PRA), Citri Reticulatae Pericarpium (CRP), Glycyrrhizae Radix et Rhizoma Praeparata Cum Melle (GRP), Zingiberis Rhizoma Recens (ZRR),* and *Jujubae Fructus (JUF)*. The YSHS granule can inhibit fibrosis in FSGS model mice through the suppression of the BMP2/Smad signaling pathway (Tan et al., 2022). YSHS granules have achieved good therapeutic results in clinical and chronic glomerulonephritis (CGN) rat models (Zhao et al., 2019). However, the regulatory mechanism of YSHS granule in regulating DN proteinuria remains unclear. One study showed that Astragalus, a component of YSHS granule, can be applied to inhibit podocyte damage induced by high glucose (Xiao,W et al., 2013). This study will investigate if YSHS granule improves the progress of DN by inhibiting podocyte apoptosis.

Exosomes are vesicles, approximately 30–120 nm in size, secreted by various types of cells and contain proteins, lipids, DNA, mRNA, and microRNAs (Zhu Q. et al., 2019). Exosomes can regulate cell activity and reprogram the phenotype of recipient cells by transporting substances in the vesicles to target cells (Khalyfa et al., 2018) and are involved in a variety of biological processes and pathogenesis of human diseases. For instance, exosomes secreted from adipose-derived stem cells attenuate DN (Jin et al., 2019). Aging-related insulin resistance can be regulated by the exosomal MiR-29b-3p derived from the bone marrow mesenchymal stem cells (Su et al., 2019). The exosomes secreted by macrophages treated by HG can reduce the expression level of a podocyte marker such as nephrin and induce podocyte pyroptosis, thus playing an important role in the progression of DN (Ding et al., 2021). However, little is known about the underlying molecular events of podocyte injury induced by macrophage-derived exosomes, and the effects of macrophage-derived exosomes on podocyte apoptosis are unknown. MicroRNAs in exosomes secreted by macrophages can be involved in the occurrence and development of a variety of diseases. For example, miR-210 in macrophage-derived exosomes can promote the onset of obesity and diabetes in mice by targeting the NDUFA4 gene (Tian et al., 2020). Therefore, the effect of macrophage-derived exosomes treated with HG on podocyte injury may be closely related to the change of miRNA content in exosomes.

The phenotypic polarization of macrophages determines kidney damage (Wang et al., 2007). Two different polarization phenotypes of macrophages depend on the renal microenvironment, which is divided into M1 (classically activated macrophages) or M2 subtypes (alternatively activated macrophages) (Guo et al., 2017). M1 is mainly involved in the promotion of inflammation, while M2 mainly has anti-inflammatory and immunomodulatory functions (Cao et al., 2011). Macrophages in DN are mainly M1 type, and the intensity of infiltration is proportional to the rate of renal function declined (Zhang et al., 2019). Overexpression of SIRT6 can promote the transformation of macrophages from M1 phenotypes to M2 phenotypes, thereby inhibiting the apoptosis-inducing effect of macrophages on podocytes stimulated by HG (Ji et al., 2019). Nicotine can change the content of miR-21–3p in macrophage-derived exosomes while promoting macrophage M1 polarization (Shaykhiev et al., 2009; Zhu J. et al., 2019). This indicates that DN progression and podocyte injury are related to M1 type macrophages, and macrophage M1 polarization may lead to changes in the miRNA content in macrophage-derived exosomes.

In this study, we explored the regulatory effects of YSHS granule on proteinuria, renal function, and podocyte apoptosis in DN mice and found that YSHS can improve the progression of DN and inhibit podocyte apoptosis. It was found that this regulatory effect may be related to the change of miRNA contents from macrophage-derived exosomes caused by M1 polarization.

## 2 Materials and methods

### 2.1 Yi-Shen-Hua-Shi (YSHS) granule

The YSHS granule (approved number: Z20090250) is a TCM drug that the National Medical Products Administration of China approved, which was provided by Guangzhou Consun Pharmaceuticals Co., Ltd. The chemical components of the YSHS granule were obtained from the reported literature (Tan et al., 2022).

### 2.2 Animal experiments

In this study, male db/m (n = 5) and db/db mice (n = 20) weighing 30–35 g were purchased from Beijing Weitong Lihua Laboratory Animal Technology Co., Ltd. (Beijing, China). All mice were fed in a 12 h light/dark cycle room, and the temperature was controlled at 24 ± 0.5°C. The db/m mice were treated with deionized water. The db/db mice were randomly divided into four groups: db/db mice + deionized water group (n = 5), db/db mice + low YSHS group (n = 5), db/db mice + medium YSHS group (n = 5), and db/db mice + high YSHS group (n = 5). After the mice were raised for 12 weeks, deionized water or YSHS granule were treated by gavage administration twice a day (10 ml/kg body weight) for 8 weeks (the experiment ended when the mice were 20-weeks-old). YSHS was obtained from Guangzhou Consun Pharmaceuticals Co., Ltd. (Guangzhou, China). The 50 g YSHS granule was dissolved in 100 ml of deionized water, and the YSHS granule stock solution was obtained after filtration (0.5 g/ml). In the high-dose group, YSHS stock solution was directly administered by gavage without dilution (0.5 g/ml). In the medium-dose group, the stock solution was diluted at 1:1 with deionized water (0.25 g/ml), and in the low-dose group, the stock solution was diluted at 1:3 with distilled water (0.125 mg/ml). The high-dose, medium-dose, and low-dose groups were given YSHS granule doses of 5, 2.5, and 1.25 g/kg body weight twice daily by gavage administered, respectively. After 8 weeks of gavage administration with deionized water or YSHS, mouse blood and 24 h urine were collected for testing, and the mouse kidney tissue was collected for fixation and embedded for pathology and fluorescence testing in subsequent experiments.

SD rats were obtained from Cavens Experimental Animal Co., Ltd. (Changzhou, China). The rats were divided into two groups and given normal saline or YSHS granule (Z20090250, Kangchen Pharmaceutical Co., Ltd., Guangzhou, China) by intragastric administration twice a day (10 ml/kg each time), which is 10 times the clinical equivalent dose (31.25 g of crude drug/kg/d, that is, 156.25 g of crude drug is dissolved in 100 ml of deionized water, filtered, and directly administered by gavage) (Shi and Wei, 2015). After intragastric treatment on the third day, the serum of rats was collected, which was the control serum and serum containing medicine (YSHS-serum).

All animal experiments were carried out according to the National Institutes of Health Guide for Care and Use of Laboratory Animals and approved by the Laboratory Animals of Zhejiang Provincial People’s Hospital.

### 2.3 Serum creatinine and urea nitrogen detection

The blood samples were taken from the abdominal aorta of the mice and placed at room temperature for 2 h, centrifuged at 3,500 r/min for 5 min, and detected by referring to the creatinine detection kit (C011-1, Nanjing Jiancheng, Nanjing, China) and BUN detection kit (C013-2, Nanjing Jiancheng, Nanjing, China).

### 2.4 Determination of urinary protein content

Urine was collected from individual mice house in a metabolic cage for 24 h before the end of 8 weeks of treatment to determine the urinary protein content. The standard substance and appropriate volume samples were added to the sample wells of a 96-well plate, and BCA working solution from the BCA protein concentration determination kit (P0010, Beyotime, Shanghai, China) was added to each well. The absorbance value of each well at 562 nm was measured using a microplate analyzer at 37°C for 30 min, and the protein concentration of the sample was calculated according to the standard curve and the sample volume used.

### 2.5 Hematoxylin-eosin staining

The tissue was dehydrated, embedded, and then placed into slices and stained with Mayer’s hematoxylin solution for 5–7 min. The slices were divided into 1% alcohol hydrochloric acid for differentiation for 2–5 s and changed back to blue. After air-drying, neutral gum was sealed, and a microscopic examination was performed.

### 2.6 TUNEL staining

The kidney tissues were collected and fixed in 4% paraformaldehyde and then embedded in paraffin. The tissues were sliced with a thickness of 10 µm. The paraffin section was dewaxed in the water, then the protease K working solution was dropped to cover the tissues, and the slides were incubated at 37°C for 25 min. The slides were washed, and the membrane-breaking working solution was dropped to cover the tissues and incubated for 20 min at room temperature. Then, reagent 1 (TdT) and reagent 2 (dUTP) were mixed at a mass ratio of 1: 9 in the TUNEL kit (Roche, China, 11684817910) and were added to the covered tissue in the circle. Then, the number of TUNEL-positive cells in the glomerular cells of mice in each group was observed and counted under a light microscope.

### 2.7 Immunofluorescence staining of kidney tissue

The paraffin section was dewaxed in the water. An appropriate amount of repair solution (0.01 M citrate buffer, pH 6.0) was added to the beaker for antigen retrieval using an electric ceramic furnace. The repair time was 15 min, followed by washing with PBS for 3 min and washing four times. We dry the slide with an absorbent paper, draw a circle around the tissue with an immunohistochemical pen, add diluted normal goat serum dropwise, and seal it for 30 min at room temperature to reduce nonspecific staining. We shake off the excess liquid, then add the diluted primary antibody dropwise, and incubate overnight (15 h) in a 4°C wet box after adding the primary antibody. It was washed four times with PBST, wiped dry with an absorbent paper, added with diluted fluorescent (cy3)-labeled goat antirabbit IgG dropwise, and incubated at 37°C for 1 h in a wet box. Dry the slides with an absorbent paper, add diluted normal goat serum dropwise, and block at room temperature for 30 min to reduce nonspecific staining. Shake off the blocking solution, and then add the diluted primary antibody dropwise. After adding the primary antibody, incubate overnight (15 h) in a 4°C humid box in the dark, add fluorescent secondary antibody, rinse three times with PBST, wipe dry, add diluted fluorescent (FITC)-labeled goat antimouse IgG, incubate at 37°C for 1 h in a wet box, and rinse four times with PBST. DAPI was added dropwise and incubated in the dark for 5 min; the specimens were stained and washed four times with PBST, and the excess DAPI (Beyotime, C1002) was washed away. The acquired images were observed under a fluorescence microscope (Olympus, BX53).

Primary antibody information: nephrin (1:100, Abcam, ab216341) and cleaved caspase-3 (1:100, Invitrogen, PA5-114687).

Secondary antibody information: fluorescent (Cy3)-labeled sheep antirabbit IgG (Wuhan Bode Bioengineering Co., Ltd, BA1032) and fluorescence (FITC)-labeled sheep against mouse IgG (Wuhan Bode Bioengineering Co., Ltd, BA1101).

### 2.8 Cell culture

RAW264.7 cell, a cell line of murine macrophage, was purchased from ATCC, using DMEM + 10% FBS medium, and subcultured at 37°C and 5% CO_2_. The mouse podocyte (MPC5) cells were purchased from ATCC, cultured using RPMI-1640 medium (11 mM d-glucose) that contained 20 U/ml γ-IFN and 10% FBS, amplified, and subcultured at 33°C and 5% CO_2_. Then, podocytes were seeded into a culture flask covered with type IV collagen (1.5 ml/25cm2) at 37°C and 5% CO_2_ for 10 days, and the medium was changed once every 2 days. The podocyte was then cultured with RPMI-1640 containing 0.2% FBS and 5.5 mM d-glucose for 24 h at 37°C and 5% CO_2_, followed by subsequent operations.

### 2.9 Exosome purification

RAW264.7 cells in the logarithmic growth phase were inoculated and cultured. Cells were treated with normal glucose (NG, 5.5 mM d-glucose) or high glucose (HG, 30 mM d-glucose) in the presence of control serum (10%) or YSHS-serum (10%) combined with FBS, which was centrifuged at 100000 g for 18 h to remove the exosomes. After RAW264.7 cells were treated for 24 h, the conditioned medium (CM) was collected. According to the instructions, an exosome extraction kit (Wako, Japan, 293–77601) was used to extract exosomes from the supernatant.

### 2.10 Transmission electron microscopy (TEM)

The exosome electron microscope analysis kit (E1610, Weihui Biotechnology, China) was used for staining observation. Put two to three drops of exosomes suspension on a clean Parafilm. Formvar/carbon-coated EM mesh (coating side down) is attached to the surface of the exosomes suspension. The EM net was used to absorb exosomes for 10 min under a dry environment. On the clean Parafilm membrane, the washing buffer was dropped. Tweezers were used to transfer the EM mesh (with the coating side down) to the washing buffer, and the mesh was left to stand for 30 s to allow the EM mesh to be washed in the washing buffer. The cleaning step was repeated. On the clean Parafilm, the EM solution was dropped and transferred to the EM mesh (with the coating side down), and the EM mesh was left to stand for 10 min. The mesh was rinsed twice according to the cleaning procedure. The EM mesh was transferred to the filter paper (coated side up) and air–dried overnight at room temperature. Exosomes were scanned under a transmission electron microscope.

### 2.11 miRNA mimics transfection

miRNA mimics NC or miR-21a-5-p mimics (GenePharma Shanghai) were transfected into podocyte MPC5 cells using Lipo6000 transfection reagent (C0526FT, Beyotime, Shanghai, China) according to the specific manufacturer’s instructions. The transfection lasted for 48 h, and the podocyte was collected for experiments.

### 2.12 CCK8

After MPC5 cells were transfected with miRNA mimics NC or miR-21a-5p mimics or treated with macrophage-derived exosomes (25 μg/ml), the commercially available CCK8 detection kit (20150520, Seven Seas) was used to detect the cell reagent of each treatment group. The operation was carried out according to the instructions, and the Multiskan MK3 Microplate reader (MD, Spectramac M3) measured the absorbance value of each well at 450 nm.

### 2.13 Caspase-3 activity assays

Detection is performed using the caspase-3 activity assay kit (Beyotime, C1115). The approximate steps are as follows: collect podocytes, centrifuge to take the supernatant after lysis for subsequent detection; add Ac-DEVD-pNA (2 mM), incubate for 60 min, and use A405 to measure the absorbance according to the standard curve (PNA-OD450) to obtain the formula; and we can obtain the caspase-3 catalysis generated in the sample, obtain its caspase-3 activity, and compare the caspase-3 activity of other groups with the control group 1 for calculation.

### 2.14 Protein concentration detection and Western blot

An appropriate amount of exosomal extract or 2 × 105 cells was taken, washed with PBS, and a lysis buffer (P0013B, Beyotime, Shanghai, China) containing PMSF (329–98–6, Nanjing Wohong, Nanjing, China) was added to lyse cells on ice for 30 min. After that, the lysate was transferred to a 1.5 ml centrifuge tube and centrifuged at 12000 rpm for 15 min at 4°C and the supernatant was taken. The protein lysate concentration was measured for protein concentration. After using the BCA protein concentration determination kit (P0010, Beyotime, Shanghai, China) to determine the protein concentration, 30 μg tissue lysate was mixed with 5x sample buffer (15 g SDS, 15.6 ml 2 M Tris pH 6.8, 57.5 g glycerol, 16.6 ml b-mercaptoethanol). The sample was loaded on a 10% polyacrylamide gel, separated by SDS-PAGE, and transferred to a PVDF membrane (Bio-Rad no.162–0177). After blocking with 4% milk containing 0.1% Tween, add primary anti-CD9 antibody (1:2000; Abcam, ab92726), CD63 antibody (1:2000; Abcam, ab216130), CD81 antibody (1:1000; Abcam, ab155760), caspase-3 antibody (1:2000; Abcam, ab184787), GAPDH antibody (1:2500; Abcam, ab9485), and incubate overnight at 4°C. After adding HRP-labeled secondary antibody (Dianova, Hamburg, Germany) and incubating at room temperature for 2 h, the ECL developer (Bio-Rad no. 170–5060) was added dropwise on the membrane and placed into the Gel Doc imaging system (Bio-Rad) for taking pictures. The protein expression level was normalized with the internal reference protein GAPDH.

### 2.15 Flow cytometric detection of the apoptosis ratio

Flow cytometry apoptosis detection kit (KGA101, Jiangsu Keygen Biotech Corp, Ltd., Nanjing, China) was used to detect the apoptosis of MPC5 cells with different treatments. The approximate steps are as follows: after the cell confluence reached 80%–90%, the cells were digested with 2.5 g/L trypsin for 2–4 min. After the cells were suspended, the digestion was terminated with serum, and a cell suspension was carefully formed. The cell suspension was filtered through a 100-mesh sieve. The filtrate was centrifuged at 2000 rpm for 5 min. The supernatant was discarded and a 2 × 105/ml cell suspension was made with PBS. The dye was added to the sample tube according to the instructions, and the mixture was mixed well and reacted overnight at 4°C in the dark. Further, 1 ml of PBS was added to each tube and mixed, and the mixture was centrifuged at 2000 rpm for 10 min to wash away the unlabeled antibodies; the supernatant was discarded, 0.5 ml of PBS was added to each tube to resuspend the cells, and a flow cytometer (BD, Accuri C6) was used to count the proportion of positive cells.

### 2.16 RNA isolation and quantitative real-time polymerase chain reaction

First, a proper amount of cells or exosomes was collected, and TRIzol (15596–026, Ambion, Texas, United States) reagent was added and the cells were lysed for 10 min. RNA was extracted according to the RNeasy Mini Kit (no. 74106, Qiagen, United States) instructions. cDNA synthesis was performed using a large-capacity cDNA Reverse Transcription Kit (no. 4368813, Applied Biosystems, United States); the qRT-PCR experiment was performed using the StepOnePlus Real-Time PCR System in the SYBRGreen experimental method (Applied Biosystems, United States), and the comparative CT value (ΔΔCT) and the U6 normalization method to determine the relative expression of genes in different samples. The PCR primer sequences and related primer sequences were listed in Table 1.

**TABLE 1:** Primer sequences for real-time PCR analysis.

Gene	Primer (5′-3′)
miR-125a-5p	TGC​GGC​TCC​CTG​AGA​CCC​TTT​AAC
miR-148a-3p	TGC​GGC​TCA​GTG​CAC​TAC​AGA​A
miR-21a-5p	TTG​CGG​CAG​CTT​ATC​AGA​CTG​A
miR universal primer	CCA​GTC​TCA​GGG​TCC​GAG​GTA​TTC
U6 (forward)	CTCGCTTCGGCAGCACA
U6 (reverse)	AAC​GCT​TCA​CGA​ATT​TGC​GT

### 2.17 Flow cytometry to detect M1 macrophage polarization

After RAW264.7 cells reached the confluence of 80%–90%, they were digested with 2.5 g/L trypsin for 2–4 min. After about 90% of the cells were suspended, the digestion was terminated with serum, and the cells were carefully pipetted to form a cell suspension. The cell suspension was filtered through a 100-mesh sieve. The filtrate was centrifuged at 2000 rpm for 5 min. The supernatant was discarded and a 2 × 105 /ml cell suspension was made with PBS. HLA-DR-FITC antibody (Abcam, ab91335, Britain) was added to the sample tube according to the instructions, and the mixture was mixed well and reacted overnight at 4°C in the dark. Further, 1 ml of PBS was added to each tube and the mixture was mixed well and centrifuged at 2000 rpm for 10 min to wash away the unlabeled antibodies; discarded the supernatant, added 0.5 ml of PBS to each tube to resuspend the cells, and a flow cytometer (cytoFLEX, Beckmancoulter, California, United States) was used to count the percentage of positive cells detected.

### 2.18 Immunofluorescence (IF) staining

The kidney tissue was embedded and cut into slices with a thickness of 10 µm. After the slice was incubated in 5% BSA (SH30574.03, Hyclone) blocking solution for 1 h, the primary anti-F4/80 antibody (1:100; Abcam, ab6640, Britain) and HLA-DR antibody (1:100; Affinity, DF6475, China) were added to tissues and incubated overnight at 4°C. Next, we added secondary antibody fluorescence (FITC)-labeled goat antirabbit IgG (1:100; BA1105, Wuhan Boster Biological Technology, LTD., Wuhan, China) and fluorescence (Cy3)-labeled goat antirat IgG (1:100; bioss, bs-0293G-CY3, China) and incubated for 1 h at 37°C in the dark. Then, it was counterstained with Hoechst or DAPI for 5 min, and pictures were taken with a fluorescence microscope.

### 2.19 Statistical analysis

The data were presented using the Prism 9.0 statistical software. All data were expressed as mean ± standard deviation (±SD). The pairwise comparison between different groups was performed using the LSD method in one-way ANOVA (least significant method). The comparison between the two groups was performed using Student’s *t*-test. *p* < 0.05 was considered statistically significant.

## 3 Results

### 3.1 YSHS granule alleviates the biochemical parameters in DN mice

**FIGURE 1:**
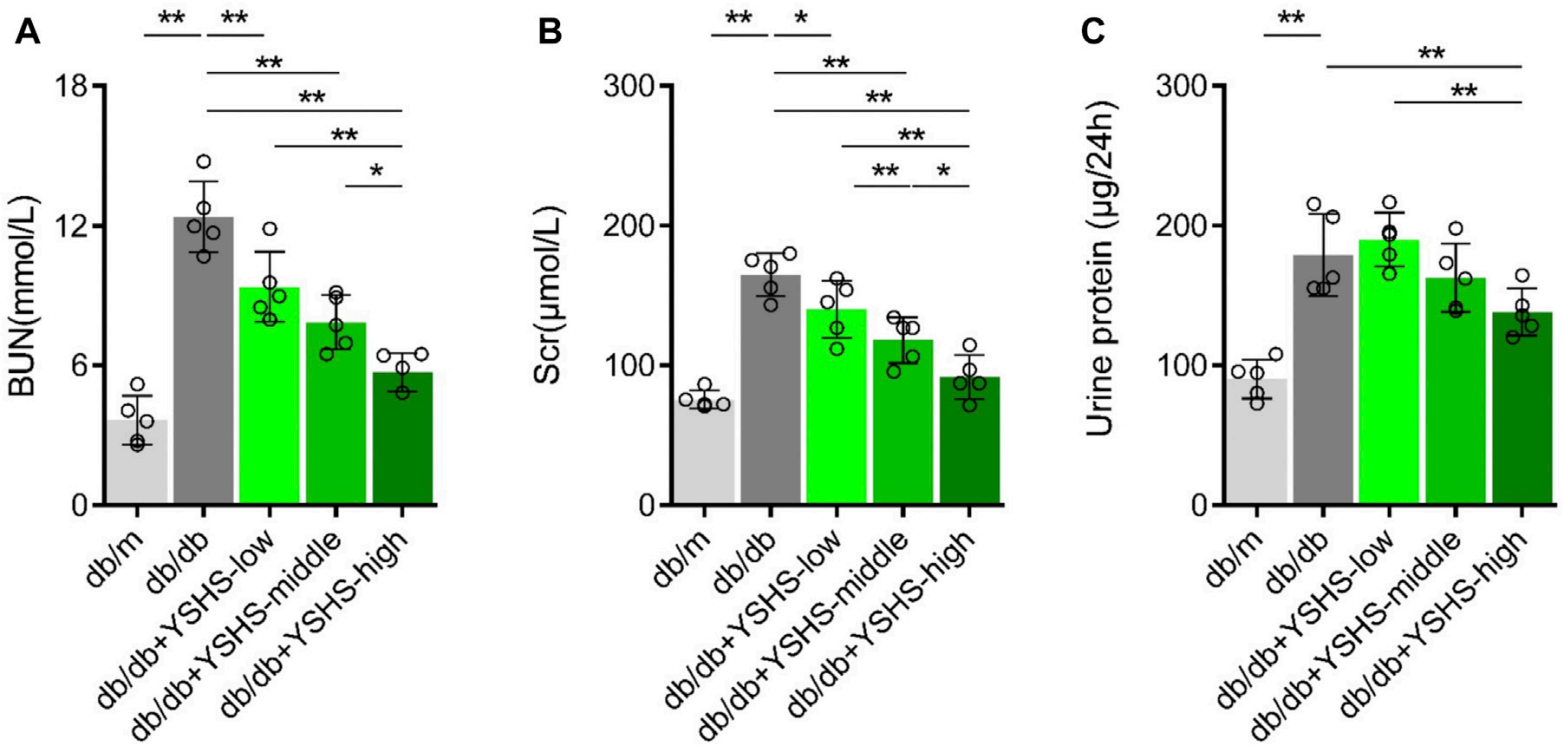
Effects of YSHS on renal function and proteinuria in db/db mice. **(A-C)** Measurement of Serum Scr, BUN levels, and 24-h-urine protein in db/m mice and db/db mice with or without a YSHS treatment. Data analysis adopts one-way ANOVA analysis of variance, and error bars indicate SD.*p < 0.05 and **p < 0.01. YSHS-low, YSHS-middle, and YSHS-high indicate the low-dose group, middle-dose group, and high-dose group of Yi-Shen-Hua-Shi granules drugs dose, respectively.

To confirm the ameliorating effect of YSHS on renal function and proteinuria in DN mice, we used the DN model db/db mice as the animal model. The detection results showed that the contents of SCr and BUN in db/db mice at the same age were significantly higher than those in db/m mice at the age of 20 weeks (*p* < 0.01 db/m group vs. db/db group, Figures 1A,B). The results also showed that the 24-h urine protein content of 20-week-old db/db mice was significantly higher than that of db/m mice (*p* < 0.01 db/m group vs. db/db group, Figure 1C). The db/db mice showed abnormal renal function and proteinuria, that is, spontaneous DN mice were successfully established.

The db/db mice were given different doses of YSHS granule by gavage administration at the age of 12 weeks. At the age of 20 weeks, the serum of mice in each group was taken for the detection of SCr and BUN, and 24 h-urine samples were collected to observe the improvement of proteinuria. The results showed that the significantly decreased SCr and BUN of db/db mice in the YSHS granule treatment group were compared to those treated with normal deionized water group (Figures 1A,B). In addition, a high dose and a medium dose of YSHS granule had a greater effect on the decrease of SCr and BUN in db/db mice, and the high-dose group had the strongest inhibitory effect (*p* < 0.05 db/db + YSHS-high vs. db/db + YSHS-middle and *p* < 0.01 db/db + YSHS-high vs. db/db + YSHS-low, Figures 1A,B). The 24-h-urine protein content of mice treated with YSHS granule significantly decreased, and the inhibitory effect of the high-dose group was significantly stronger than that of the low-dose group (*p* < 0.01 db/db + YSHS-high vs. db/db + YSHS-low, Figure 1C). In addition, another batch of animal experiments also showed that YSHS granule can significantly reduce the 24-h-urine protein content and kidney function of DN mice (Supplementary Figure S2A–S2C). It shows that YSHS granule can significantly ameliorate renal function and proteinuria of DN mice, and the high-dose group has the strongest inhibitory effect.

### 3.2 YSHS granule could significantly improve the abnormal histology and the apoptotic level of glomerular cells in DN mice

**FIGURE 2:**
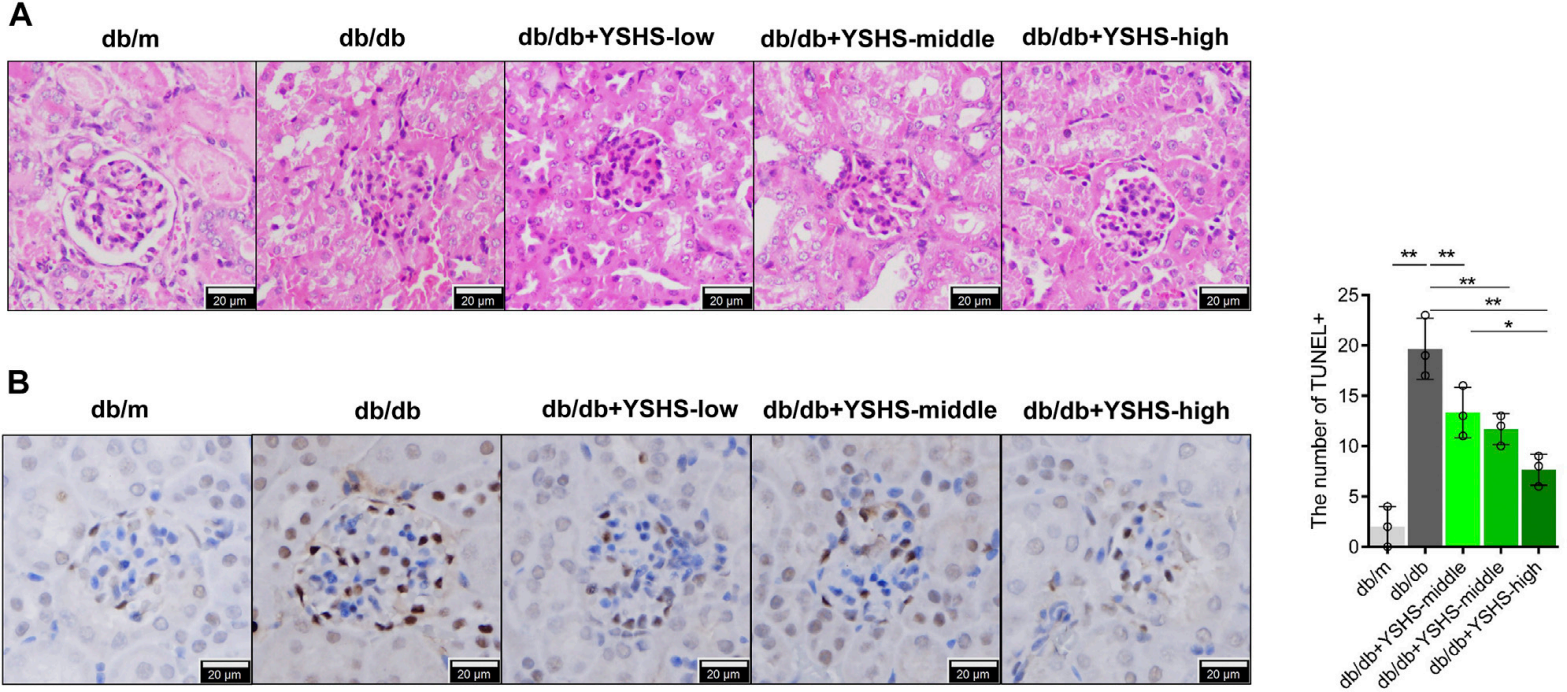
Effects of YSHS on glomerular structural lesions and glomerular cell apoptosis in db/db mice. **(A)** HE detected the glomerular structure of each group of mice. **(B)** TUNEL detected the glomerular apoptosis and statistical results of each group of mice. Data analysis adopts a one-way ANOVA analysis of variance, and error bars indicate SD.*p < 0.05 and **p < 0.01. YSHS-low, YSHS-middle, and YSHS-high indicate the low-dose group, middle-dose group, and high-dose group of Yi-Shen-Hua-Shi granules drugs dose, respectively.

**FIGURE 3:**
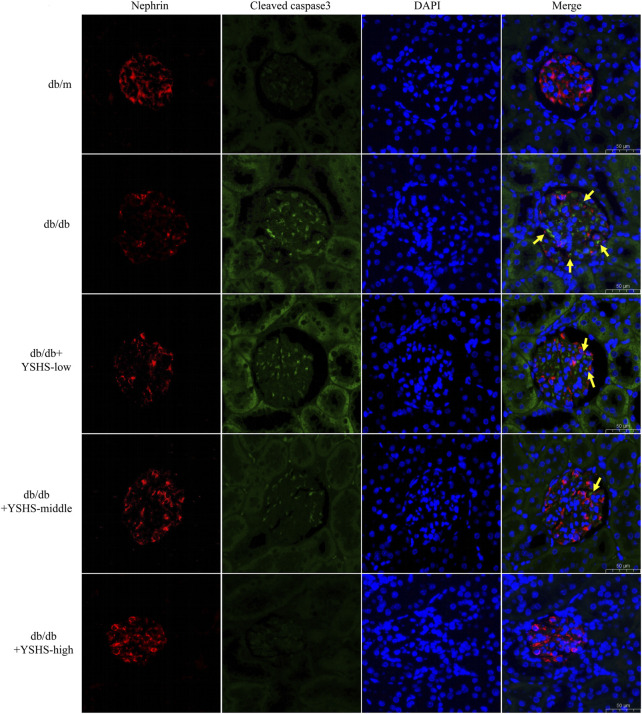
Immunofluorescence detection of the expression level of cleaved caspase-3 in podocytes in the kidney tissue of DN mice. Red indicates the podocyte marker nephrin, green indicates cleaved caspase-3, and yellow arrows indicate cleaved caspase-3 staining sites in nepherin + cells; the size of the scale is 50 μm.

To detect the effects of YSHS granule on glomerular structure pathology and podocyte apoptosis in DN mice, HE staining, TUNEL staining, and immunofluorescence (IF) staining were used. Compared with db/m mice, the renal glomerular volume and mesangial matrix of db/db mice increased, while intragastric administration of YSHS granule could reduce the increase of glomerular volume and mesangial matrix in db/db mice (Figure 2A). In addition, compared with db/m mice, the number of TUNEL+ cells in renal glomerulus of db/db mice increased (Figure 2B). Compared with db/db mice not treated with YSHS granule, the number of TUNEL + cells in the renal glomerulus of db/db mice treated with YSHS granule decreased, and the number of TUNEL + cells in renal glomerulus decreased with the increase of YSHS granule (Figure 2B). The result of IF detection showed that the apoptosis level of podocytes in the kidney tissue of DN model mice is increased, and compared with control db/db mice, the fluorescence expression intensity of podocyte marker nephrin in the kidney tissue of db/db mice treated with YSHS granule increased, while the fluorescence expression intensity of cleaved caspase-3 in nephrin + cells decreased in a dose-dependent manner (Figure 3). The fluorescence expression intensity of nephrin and cleaved caspase-3 in the kidney tissue of db/db mice treated with a high-dose YSHS granule by gavage was similar to that of db/m mice (Figure 3). It indicated that YSHS granule could alleviate the apoptosis of podocytes in the kidney tissue of DN. In addition, another batch of animal experiments also showed that YSHS granule can alleviate the apoptosis of podocytes in the kidney tissue of DN (Supplementary Figure S3). All results showed that YSHS granule could improve glomerular structural lesions and podocyte apoptosis in DN model mice in a dose-dependent manner.

### 3.3 Exosomes from macrophages treated with HG and YSHS granule had lower effects on podocyte injury

**FIGURE 4:**
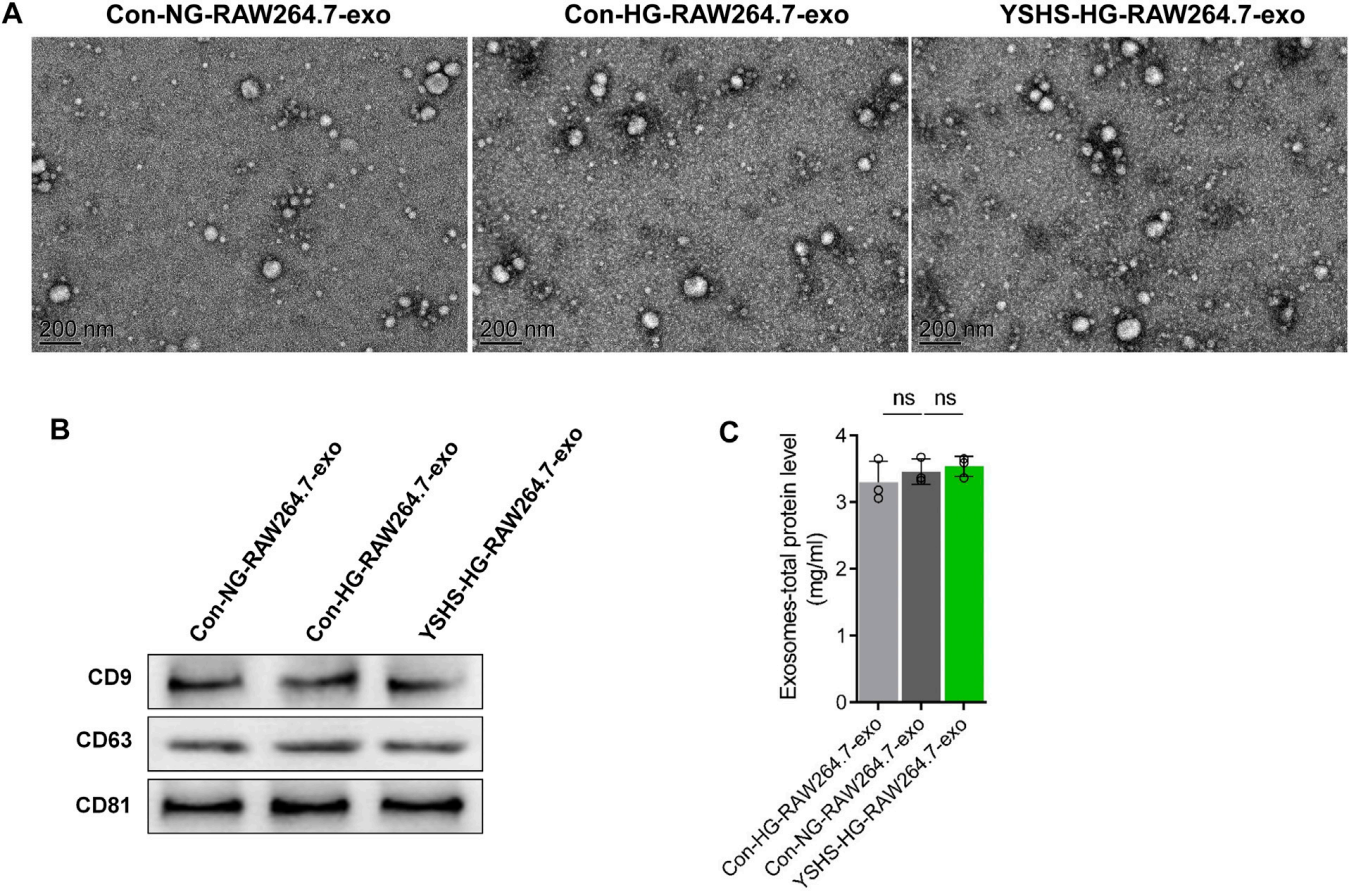
Identification of macrophage exosomes and the effects of YSHS and high-glucose stimulation on macrophage-derived exosome secretion. **(A)** Images of macrophage-derived exosome morphology taken by transmission electron microscopy (TEM). **(B)** Measurement of the protein level of CD9, CD63, and CD81 of macrophage-derived exosomes by Western blotting. **(C)** The protein concentration of macrophage-derived exosomes was detected by the BCA method. Data analysis adopts a one-way ANOVA analysis of variance. SD indicates error bars ns *p* > 0.05. NG indicates normal glucose (5.5 mM d-glucose), and HG indicates high glucose (30 mM d-glucose). Con-NG-RAW264.7-exo indicates exosomes from macrophage RAW264.7 cells treated with normal glucose and 10% control serum, Con-HG-RAW264.7-exo represents exosomes from macrophage RAW264.7 cells treated with high glucose and 10% control serum, and YSHS-HG-RAW264.7-exo represents exosomes from macrophage RAW264.7 cells treated with high glucose and 10% YSHS-serum.

To explore whether YSHS granule can change the effect of macrophage-derived exosomes on podocyte injury, the supernatant of macrophages RAW264.7 treated with normal glucose or HG combined with control serum or YSHS-Serum was collected. Then, exosomes from the supernatant were isolated by the exosome extraction kit. The purified three groups of macrophage-derived exosomes were identified by transmission electron microscopy and Western blotting. TEM showed that the exosomes of macrophages in the three groups were typical round vesicles with a diameter of about 30–100 nm, which was consistent with the characteristics of exosomes (Figure 4A). As shown in Figure 4B, the expression of surface markers of exosomes (CD9, CD63, and CD81) in the three groups could be detected by Western blot. It shows the successful isolation of exosomes from the three groups of macrophages. The BCA method was used to detect the protein concentration of three groups of macrophage-derived exosomes. These results indicated that the HG treatment and YSHS granule treatment did not influence the protein concentration of macrophage-derived exosomes (Figure 4C). In conclusion, these data consistently supported that HG stimulation and YSHS granule do not affect the secretion of macrophage-derived exosomes.

**FIGURE 5:**
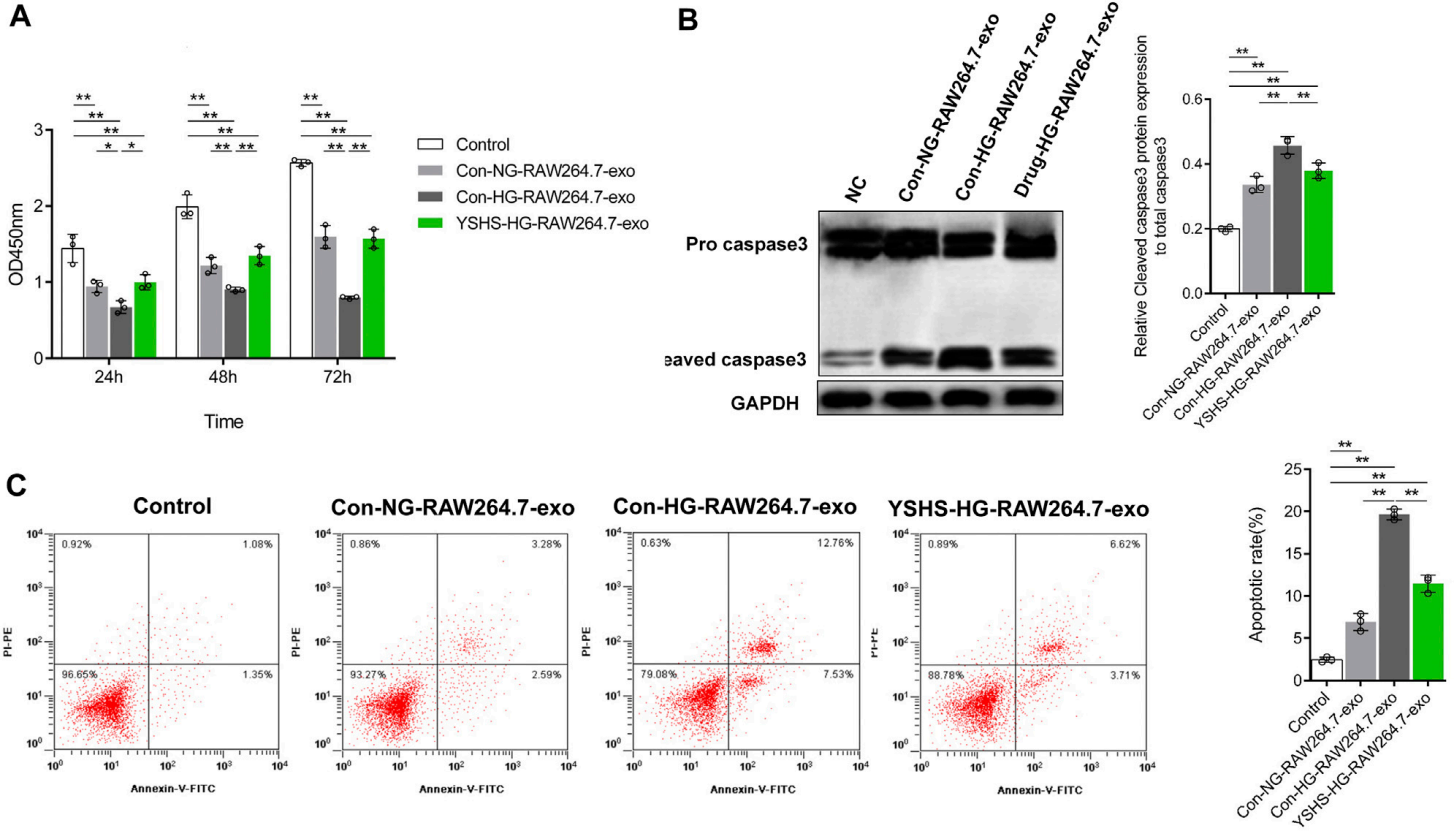
Effect of exosomes secreted by macrophages treated with HG and (or) YSHS on podocyte MPC5 cell injury. **(A)** Measurement of the activity of MPC5 cells treated with different kinds of macrophage-derived exosomes (25 μg/ml) by CCK8. **(B)** Western Blot was used to detect the caspase-3 cleavage of podocyte MPC5 cells in each treatment group. **(C)** Measurement of the level of apoptosis of MPC5 cells by flow cytometry. Data analysis adopts a two-way ANOVA (A) or a one-way analysis (C, E) of variance. SD indicates error bars. *p < 0.05 and **p < 0.01.

Then, the changes in the cell activity of podocytes treated with macrophage-derived exosomes at different times were detected by the CCK8 experiment. The data showed that compared with MPC5 cells without any treatment, the cell activity of MPC5 cells decreased significantly after treatment with macrophage-derived exosomes, and the longer the treatment time, the stronger the inhibition of the cell activity (Figure 5A). In addition, macrophage-derived exosomes could also promote caspase-3 shearing in podocytes and increase the level of podocyte apoptosis (Figure 5B). Among them, compared with the group treated by the exosomes secreted by macrophages treated with control serum and normal glucose, the group treated by the exosomes secreted by macrophages treated with control serum and HG had stronger effects on the podocyte activity, caspase-3 shearing, and apoptosis (Figure 5B), while the group treated by the exosomes secreted by macrophages treated with YSHS granule and HG had significantly lower effects on the podocyte activity, caspase-3 shearing, and apoptosis (Figure 5B), compared with the group treated by the exosomes secreted by macrophages treated with control serum and HG. In addition, another batch of cell experiments also showed the YSHS can change the effect of macrophage-derived exosomes on podocyte injury (Supplementary Figure S4). Compared to without any treatment (control group), the activity of caspase-3 in podocytes after macrophage-derived exosomes treatment increased, the activity of caspase-3 in podocytes in the HG-induced macrophage-derived exosomes treatment was further increased, and the activity of podocytes in the YSHS granule treatment was significantly reduced compared with the HG-induced macrophage-derived exosomes treatment group (Supplementary Figure S8A). The results showed that compared with the exosomes secreted by macrophages treated with HG, the exosomes secreted by macrophages treated with YSHS granule and HG significantly reduced the promotion of apoptosis of podocytes. The results showed that YSHS granule could inhibit the induction of podocyte injury by macrophage-derived exosomes after an HG treatment, and it was directly realized by regulating the function of macrophage-derived exosomes.

### 3.4 YSHS granule could block the upregulating effects of macrophage-derived exosomes on the miR-21a-5p content in podocytes by lowering the macrophage-derived exosomal miR-21a-5p content

**FIGURE 6:**
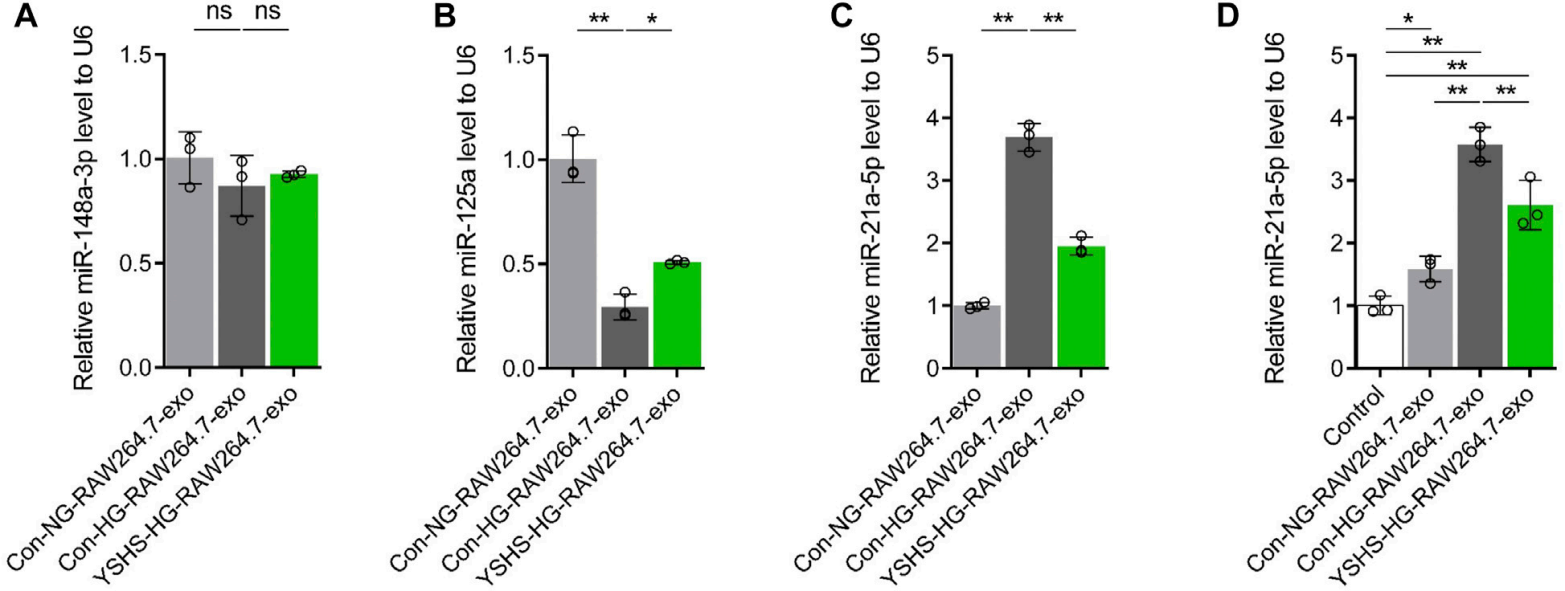
Effect of exosomes secreted by macrophages treated with high glucose and (or) YSHS-serum on the content of miR-21a-5p in podocyte MPC5 cells. **(A-C)** Effect of medicated serum of YSHS granule on the contents of miR-148a-3p, miR-125a, and miR-21a-5p in macrophage-derived exosomes induced by HG. **(D)** Measurement of the miR-21a-5p content by qRT-PCR in MPC5 cells with different treatments. Data analysis adopts a one-way analysis of variance. SD indicates error bars. *p < 0.05 and **p < 0.01.

Our previous studies found that the function of exosomes is closely related to miRNA (Jin et al., 2019; Jin et al., 2020). It has been found that miR-125a can improve the hepatic glucose and lipid metabolism disorder of type II diabetes (Xu et al., 2018), and miR-148a-3p can slow down diabetic retinopathy (Wang et al., 2020). The expression of miR-21a-5p increased in the PBMCs of diabetic patients (Salas-Pérez et al., 2013). To clarify that the improvement of macrophage-derived exosomes functions by YSHS granule is related to the changes of the previously mentioned miRNA content in macrophage-derived exosomes, we first detected the contents of miR-125a, miR-148a-3p, and miR-21a-5p in macrophage-derived exosomes. qRT PCR data showed that compared with the normal glucose and control group, the content of miR-125a in the exosomes secreted by macrophages after HG and control serum treatment decreased significantly, accompanied by the increase of the miR-21a-5p content, but the content of miR-148a-3p did not change significantly (Figures 6A–C). In addition, the contents of miR-125a and miR-21a-5p in the exosomes secreted by macrophages treated with HG and YSHS granule were improved compared with those of macrophages treated with HG and control serum, while the content of miR-148a-3p remained unchanged (Figures 6A–C). The results showed that HG stimulation could change the contents of miR-125a and miR-21a-5p in macrophage-derived exosomes, while YSHS granule could regulate macrophages to block the change of miR-125a and miR-21a-5p contents in macrophages-derived exosomes after HG stimulation.

Exosomes play a therapeutic role mainly by transferring various miRNAs or growth factors to recipient cells (Jin et al., 2019). To clarify whether the podocyte injury induced by macrophage-derived exosomes is related to miRNA, we selected miR-21a-5p, which is consistent with the changing trend of podocyte injury for the follow-up study. Podocyte RNA samples treated with macrophage-derived exosomes were collected, and then the content of miR-21a-5p was detected by qRT-PCR. The data showed that macrophage-derived exosomes could increase the content of miR-21a-5p in podocytes compared with podocytes without any treatment, and the exosomes secreted by macrophages treated with HG and control serum had the strongest promoting effect on the content of miR-21a-5p in podocytes, compared with the macrophage-derived exosomes treated with HG and control serum, the exosomes of macrophages treated with HG and YSHS granule decreased the promotion of the miR-21a-5p content in podocytes (Figure 6D). It was suggested that the content of miR-21a-5p in macrophage-derived exosomes can affect the content of miR-21a-5p in podocytes and YSHS granule may block the promoting effect of macrophage-derived exosomes on the content of miR-21a-5p in podocytes by reducing the content of miR-21a-5p in macrophage-derived exosomes.

### 3.5 miR-21a-5p could induce podocyte injury

**FIGURE 7:**
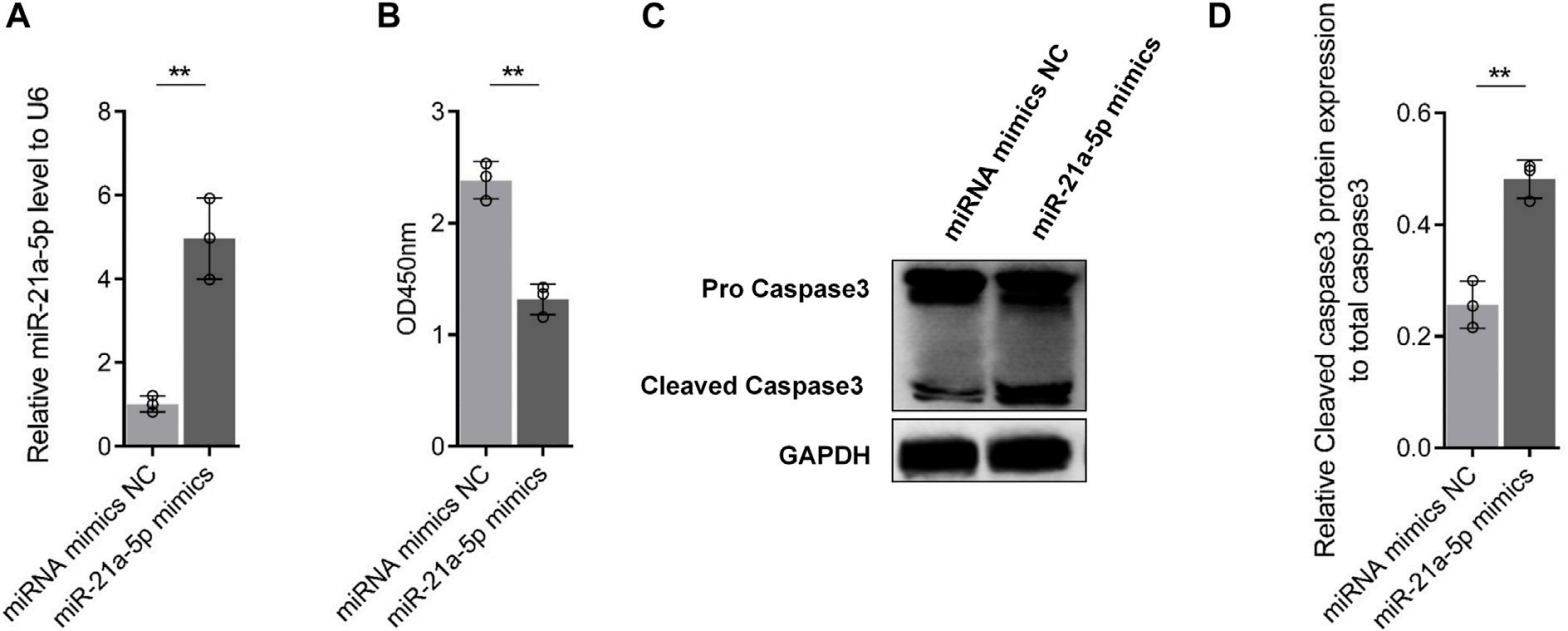
Effect of miR-21a-5p on podocyte injury. **(A)** Effect of miR-21a-5p mimics transfection on the content of miR-21a-5p in MPC5 cells. **(B)** Effect of miR-21a-5p mimics transfection on MPC5 cell viability. **(C-D)** Effect of miR-21a-5p on the cleavage of apoptotic protein caspase-3 in MPC5 cells. Data analysis adopts Student’s *t*-test. SD indicates error bars. *p < 0.05 and **p < 0.01.

Podocytes were treated with miR-21a-5p mimics or miRNA mimics NC transfection to explore the effect of miR-21a-5p on podocyte injury. Figure 7A shows that miR-21a-5p mimics could significantly increase the miR-21a-5p content in podocytes (nearly 5 times). The CCK8 experimental data showed that the podocyte activity of the miR-21a-5p mimics transfection group was significantly lower than that of the miRNA mimics NC treatment group (Figure 7B). The results of the Western blot experiment were consistent with those of the CCK8 experiment. miR-21a-5p mimics could significantly promote caspase-3 shearing (Figures 7C,D). Similarly, compared with the miRNA mimics NC treatment group, the activity of podocytes caspase-3 in the miR-21a-5p mimics transfection group was significantly increased. The results showed that miR-21a-5p could induce apoptosis in podocytes (Supplementary Figure S8B). From the aforementioned results, it can be seen that miR-21a-5p could induce podocyte injury by promoting the apoptosis level of podocytes.

### 3.6 YSHS granule may change the content of miR-21a-5p in macrophage-derived exosomes by inhibiting macrophage M1 polarization

Macrophage polarization plays an important role in the progression of diabetic nephropathy and podocyte injury (Ji et al., 2019; Zhang et al., 2019), and macrophage polarization may change the miRNA content of exosomes (Shaykhiev et al., 2009; Zhu J. et al., 2019). Some studies have pointed out that miR-21a-5p can promote M1 polarization of macrophages (Gao et al., 2018). Human M2 macrophage-derived exosomes and mouse M1 macrophage-derived exosomes are rich in miR-21a-5p (Chang et al., 2021; Lu et al., 2021). To analyze the correlation between the change of the miR-21a-5p content in macrophage-derived exosomes and macrophage M1 polarization, we first induced macrophage M1 polarization, and then analyzed the change of the miR-21a-5p content in M1 macrophage-derived exosomes. The results of flow cytometry showed that after 24 h of

**FIGURE 8:**
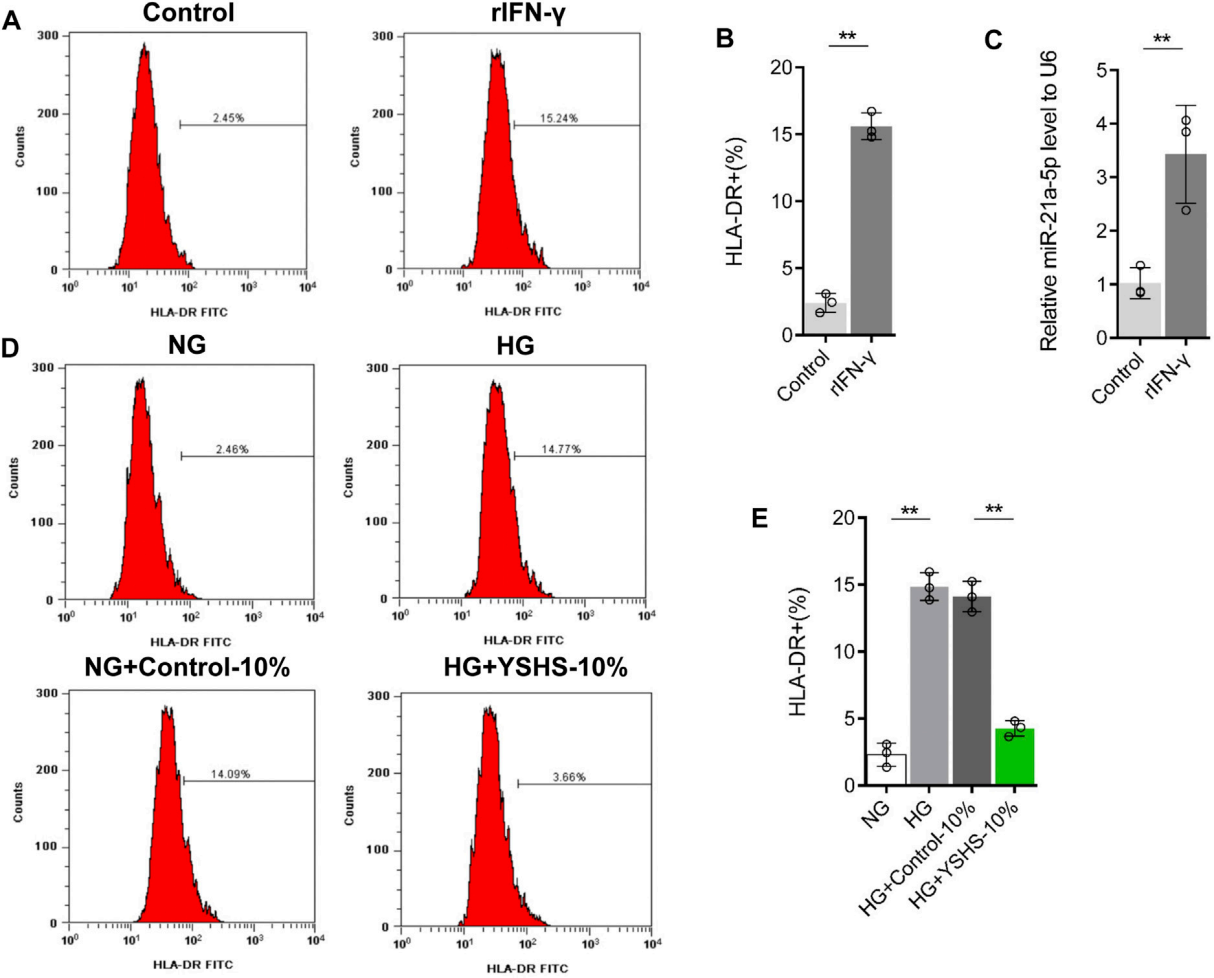
Effect of YSHS on the polarization of M1 macrophages. **(A–B)** Flow cytometry to detect the expression of HLA-DR in the macrophage treated with or without LPS (100 ng/ml) and recombinant IFN-γ (20 ng/ml) for 24 h. **(C)** Measurement of the miR-21a-5p content in macrophage-derived exosomes by qRT-PCR. **(D–E)** Flow cytometry to detect the expression of HLA-DR in macrophages treated with or without YSHS-serum in the presence of NG or HG. Data analysis adopts a Student’s *t*-test **(B–C)** or a one-way analysis of variance (E). LPS + rIFN-γ represents that macrophage RAW264.7 cells were treated with LPS (100 ng/ml) and recombinant IFN-γ (20 ng/ml) for 24 h, NG indicates that macrophage RAW264.7 cells were treated with normal glucose, HG represents that macrophage RAW264.7 cells were treated with high glucose, HG + control-serum indicates that macrophage RAW264.7 cells were treated with high glucose and 10% control serum, and HG + YSHS-serum represents that macrophage RAW264.7 cells were treated with high glucose and 10% YSHS-serum. SD indicated error bars. **p < 0.01.

LPS and recombinant protein IFN-γ treatment, the content of an M1 marker HLA-DR on the surface of macrophages increased significantly, that is, M1 macrophages were successfully induced (Figures 8A,B). Then, we obtained the exosomes in the supernatant of M1 macrophages with the exosome extraction kit. Through Western blot detection, it was found that the exosomes extracted from the noninduced group and M1 macrophage-induced group expressed exosome markers CD9, CD63, and CD81, indicating that the exosomes of the two groups were extracted successfully (Supplementay Figure S1A). Through the BCA protein concentration determination kit, it was found that there was no significant difference in the concentration of macrophage-derived exosome protein between the two groups (*p* > 0.05), indicating that M1 macrophage polarization induction did not change macrophage-derived exosome secretion (Supplementary Figure S1B). Subsequently, the qRT-PCR experiment showed that compared with the noninduced treatment group, the content of miR-21a-5p in exosomes from the M1 macrophage-induced treatment group increased (Figure 8C). The results showed that the content of miR-21a-5p in macrophage-derived exosomes was significantly upregulated after M1 polarization.

**FIGURE 9:**
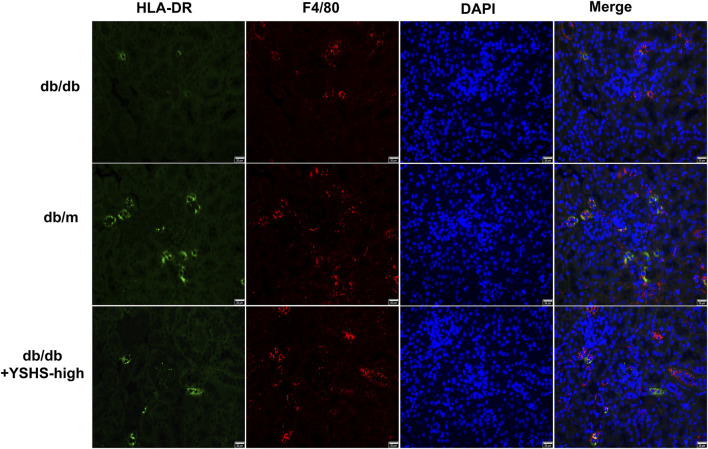
Evaluation of the role of YSHS in the alteration of macrophage infiltration and M1 polarization in kidney tissues in db/db mice. IF detected the distribution of HLA-DR and F4/80 fluorescence in the kidney tissues of mice in each group. The size of the scale is 20 μm.

To clarify that the effects of HG stimulation and YSHS granule on the content of miR-21a-5p in macrophage-derived exosomes may be related to macrophage M1 polarization, we analyzed the effects of HG stimulation and YSHS granule treatments on macrophage M1 polarization. The results of flow cytometry showed that HG stimulation increased the proportion of HLA-DR + cells compared with the normal glucose stimulation group; the control serum did not change the expression of HLA-DR on macrophages, compared with control serum and the HG treatment group, YSHS granule treatment inhibited the promotion of HG stimulation on HLA-DR expression in macrophages (Figures 8D,E). In addition, we tested the effect of YSHS on macrophage infiltration and M1 polarization in the kidney tissue of db/db mice through IF. It was found that compared with db/m mice there were more F4/80 + cells and F4/80+HLA-DR + cells in the kidney tissue of db/db mice. The gavage treatment of high-dose YSHS granule has little effect on the infiltration of F4/80 + cells in mouse kidney tissue, but it can significantly inhibit the increase of F4/80+HLA-DR + cells (Figure 9). This indicates that although YSHS granule does not affect the macrophage infiltration in the kidney tissue of DN mice, YSHS granule can play a regulatory role by inhibiting the M1 polarization of renal macrophages. It was suggested that YSHS granule can inhibit macrophage M1 polarization and infiltration of M1 macrophages in DN kidneys, thus affecting the content of miR-21a-5p in macrophage-derived exosomes.

## 4 Discussion

Small-scale clinical research reveals that YSHS granule can inhibit SCr and BUN and urinary protein contents in DN patients and improve renal injury (Chen, 2018; Fang et al., 2020; Liu,C et al., 2018; Zhang, 2020). YSHS granule can improve renal function and proteinuria (Zhao and Dong, 2016) in diabetic rats induced by HG and a high-fat diet. In this study, we found that YSHS granule could significantly improve the renal function, proteinuria, glomerular volume, and mesangial matrix of other DN model db/db mice, which further confirmed that YSHS granule can improve the progression of DN. The results once again confirmed the important potential of TCM in the treatment of chronic disease DN. In addition, we found that YSHS granule improved renal function and proteinuria in DN mice in a dose-dependent manner. At the clinical level, whether high-dose YSHS granule has a better DN improvement effect and whether high-dose YSHS granule has side effects that need to be further explored.

The occurrence of DN has been closely related to podocyte fall off after being stimulated by HG. Inhibition of podocyte apoptosis can improve DN progression (Qi et al., 2017; Jin et al., 2019). Macrophages play an important role in podocyte injury in DN. For example, the supernatant of macrophages stimulated by HG can induce podocyte injury, including decreased expression of podocyte markers, decreased migration ability, and increased apoptosis levels (Ji et al., 2019; Yang et al., 2019). Exosomes are membrane vesicles secreted by cells and released into the extracellular matrix. They are important mediators for cells to exert paracrine function. It has been found that macrophages could regulate inflammation through exosomes to affect wound healing and podocyte injury (Wang et al., 2019; Huang et al., 2020). Our study confirmed that macrophage-derived exosomes can inhibit a podocyte activity and induce podocyte apoptosis, and the exosomes secreted by macrophages after HG stimulation have a stronger induction effect on podocyte injury. Some studies have found that the improvement of TCM on the progress of DN is related to its inhibitory effect on podocyte injury. Huangqi decoction can inhibit podocyte apoptosis *in vivo* and *in vitro* through a NOX4/p53/Bax pathway (Li et al., 2019). In this study, we found that YSHS granule could inhibit the level of glomerular cell apoptosis in DN model mice in a dose-dependent manner. Podocytes are located in the glomerulus. *In vitro* experiments showed that compared with macrophage-derived exosomes stimulated by HG, the exosomes secreted by macrophages treated with YSHS granule had a lower induction effect on podocyte apoptosis. *In vivo* and *in vitro* experiments suggested that YSHS granule could inhibit the podocyte injury induced by macrophage-derived exosomes by regulating the function of macrophage-derived exosomes, resulting in an improvement of DN progression. It further showed that the TCM could target podocyte injury and alleviate the progress of DN.

Exosomes are rich in noncoding RNA, protein, and lipid, which can change the function of target cells by transmitting substances in exosomes to target cells. Our previous studies found that ADSCs-Exo changes the contents of miR-486 and miR-215–5p in podocytes by transmitting miR-486 and miR-215–5p in exosomes to podocytes, to alleviate podocyte apoptosis, and EMT process induced by high glucose (Jin et al., 2019; Jin et al., 2020). Some studies have pointed out that podocytes can phagocytize macrophage-derived exosomes, resulting in miR-25–3p in macrophage-derived exosomes entering podocytes, changing the content of miR-25–3p in podocytes and HG-induced injury of podocytes (Huang et al., 2020). miR-21a-5p is upregulated in PBMCs of diabetic patients, which can regulate diabetic retinopathy (Chen et al., 2017) and diabetic heart dysfunction (Dai et al., 2018). Macrophages can exacerbate enteritis through secreted miR-21a-5p (Lu et al., 2021). We found that HG stimulation did not change the secretion concentration of macrophage-derived exosomes. The content of miR-21a-5p in macrophage-derived exosomes increased after HG stimulation; macrophage-derived exosomes could increase the content of miR-21a-5p in podocytes. When the content of miR-21a-5p in macrophage-derived exosomes decreased, its promoting effect on the content of miR-21a-5p in podocytes decreased. Also, miR-21a-5p could induce a decrease in the podocyte activity. It is suggested that macrophage-derived exosomes could change the content of miR-21a-5p in podocytes and induce podocyte apoptosis by transmitting exosomal miR-21a-5p to podocytes. In addition, a recent study confirmed that HG stimulation increased the content of miR-21–5p in macrophage-derived exosomes, and it is found that reducing the content of miR-21–5p in macrophage-derived exosomes can improve the inhibitory effect of macrophage-derived exosomes on the expression of podocyte marker nephrin and the induction of inflammation (Ding et al., 2021). The results showed that the podocyte injury induced by macrophage-derived exosomes was closely related to the change of the miR-21a-5p content in exosomes.

The effect of TCM is often closely related to an anti-inflammatory effect. In our study, we found that compared with the exosomes from macrophages stimulated by HG, the miR-21a-5p content of exosomes secreted by macrophages treated with HG and YSHS granule decreased, and its induction of podocyte injury decreased. It was suggested that the alleviating effect of YSHS Granule on podocyte injury in DN may be achieved by altering the content of miR-21a-5p in macrophage-derived exosomes.

The main pathological type of diabetic kidney tissue is M1-type proinflammatory macrophage infiltrating (Zhang et al., 2019). Inhibiting M1 polarization of macrophages can improve the induction of podocyte injury by macrophages and alleviate the progress of DN (Ji et al., 2019). In addition, M2 macrophage-derived exosomes can inhibit HG-induced podocyte injury (Shaykhiev et al., 2009). It is suggested that the regulatory effect of macrophage-derived exosomes on podocytes is related to the polarization state of macrophages. In this study, it was found that the content of miR-21a-5p in macrophage-derived exosomes was significantly upregulated after M1 polarization. The YSHS granule could improve the M1 polarization of macrophages induced by HG and inhibit the infiltration of M1 macrophages in the renal tissue of DN model mice. In addition, studies have pointed out that the promotion of enteritis by mouse M1 macrophage-derived exosomes depends on miR-21a-5p in exosomes (Lu et al., 2021). miR-21a-5p can promote M1 polarization of macrophages and inhibit M2 polarization (Gao et al., 2018). It is speculated that the content of miR-21a-5p in macrophage-derived exosomes increases during macrophage M1 polarization. The YSHS granule may reduce the content of miR-21a-5p in macrophage-derived exosomes by inhibiting macrophage M1 polarization and slowing down the induction of macrophage-derived exosomes on podocyte injury. Whether the intracellular miR-21a-5p content changes during macrophage M1 polarization and whether the M1 polarization inducer promotes macrophage M1 polarization by regulating the expression of miR-21a-5p remains to be further studied.

## 5 Conclusion

In short, the YSHS granule can inhibit M1 polarization of macrophages and M1 macrophage infiltration in renal tissue, reduce the content of miR-21a-5p in macrophage-derived exosomes, and reduce the promoting effect of macrophage-derived exosomes on the content of miR-21a-5p in podocytes to improve podocyte injury and alleviate the progress of DN. Potentially, this is a novel function of YSHS granule and has promising future clinical applications. To sum up, this study provides a new theoretical basis for the treatment of DN with YSHS granule.

## Data Availability

The original contributions presented in the study are included in the article/Supplementary Material; further inquiries can be directed to the corresponding authors.

## Abbreviations

YSHS, Yi-Shen-Hua-Shi granule. DN, diabetic nephropathy. ESRD, end-stage renal disease. GBM, glomerular basement membrane. TEM, transmission electron microscopy.
