# Supplementary figures and images for: Yi-Shen-Hua-Shi granules inhibit diabetic nephropathy by ameliorating podocyte injury induced by macrophage-derived exosomes (part 1 of 2)
Source: Front Pharmacol. 2022 Nov 25;13:962606. doi: 10.3389/fphar.2022.962606 (PMC9732029; doi:10.3389/fphar.2022.962606)

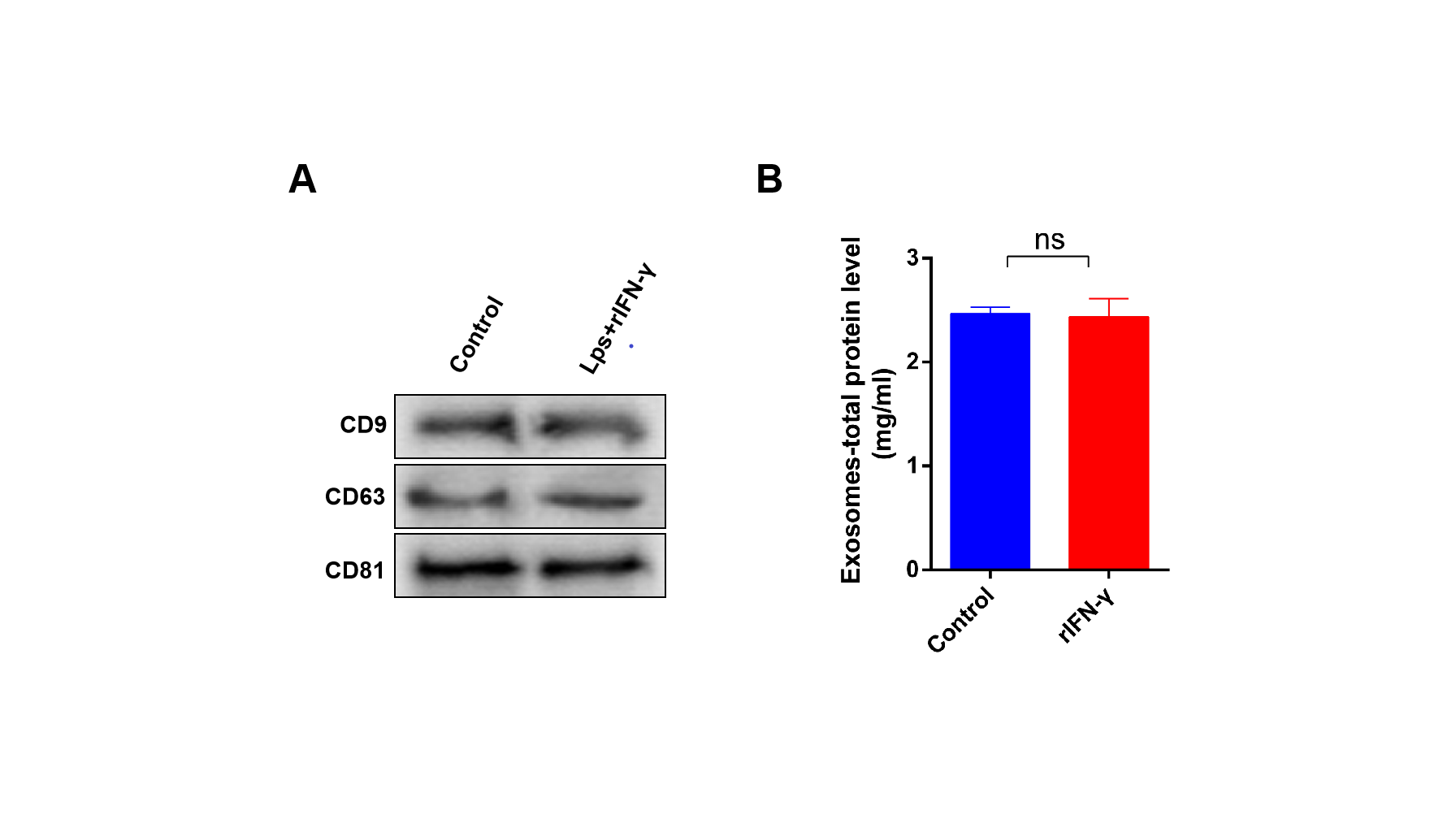

Supplement: Supplementary file 1 [file Image1.TIFF]

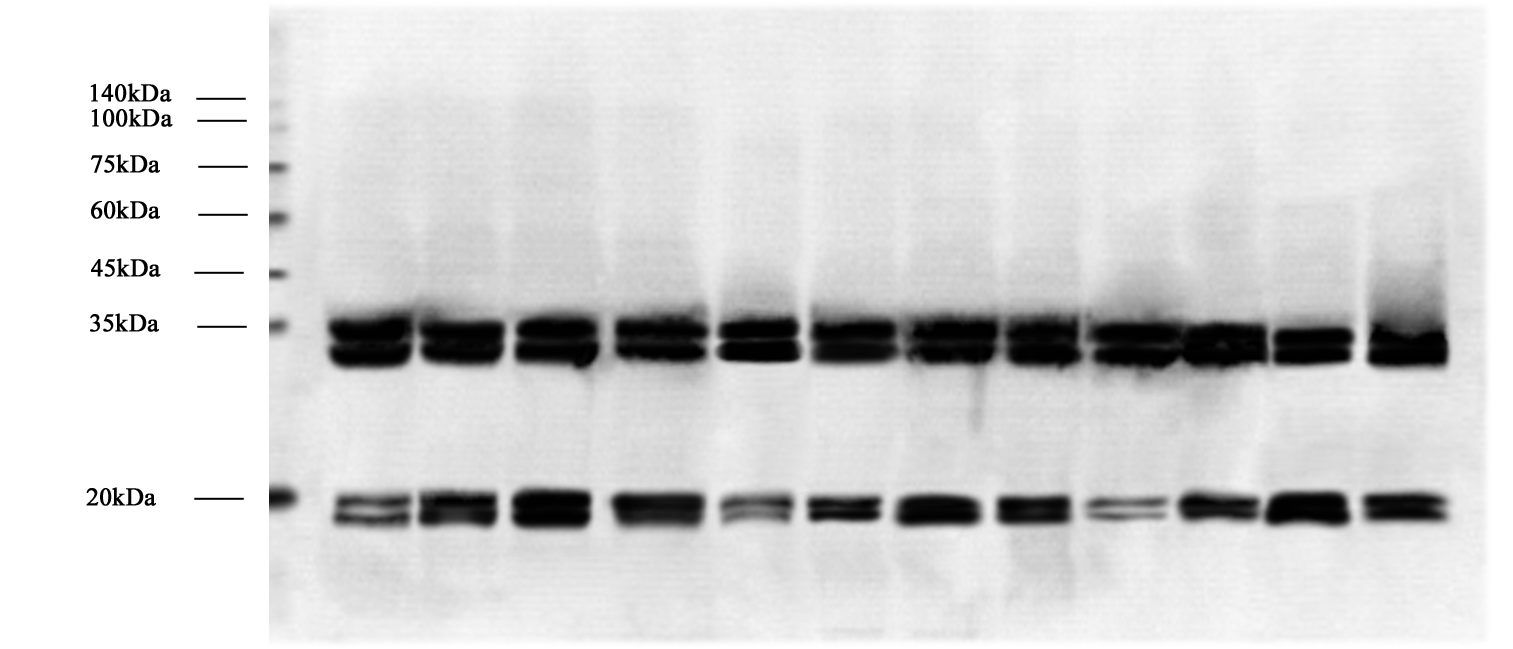

Supplement: Supplementary file 2 [file DataSheet3.ZIP › original data FIG4-7/Fig4B-C caspase3剪切/caspase3-marker.tif]

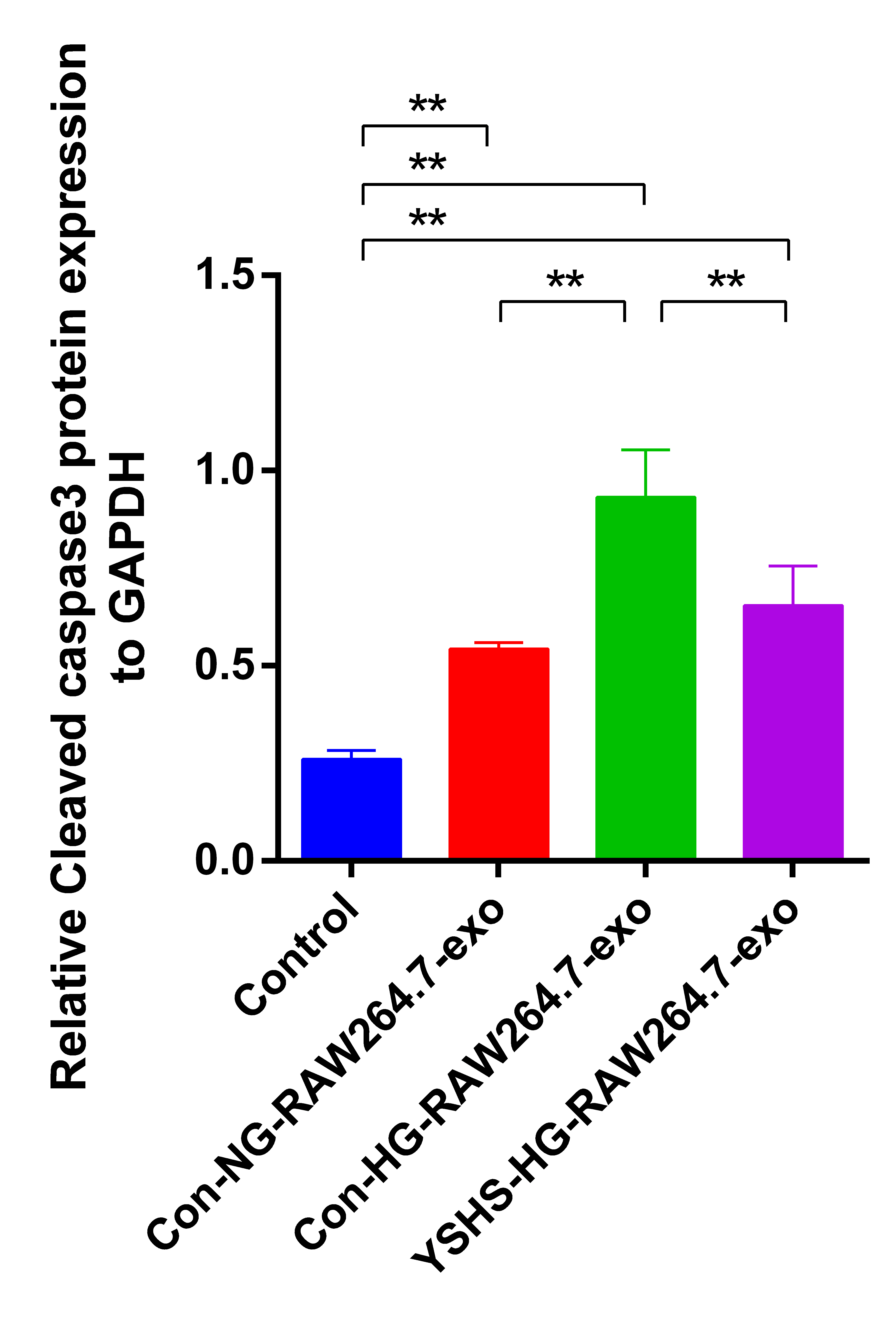

Supplement: Supplementary file 2 [file DataSheet3.ZIP › original data FIG4-7/Fig4B-C caspase3剪切/Cleaved.jpg]

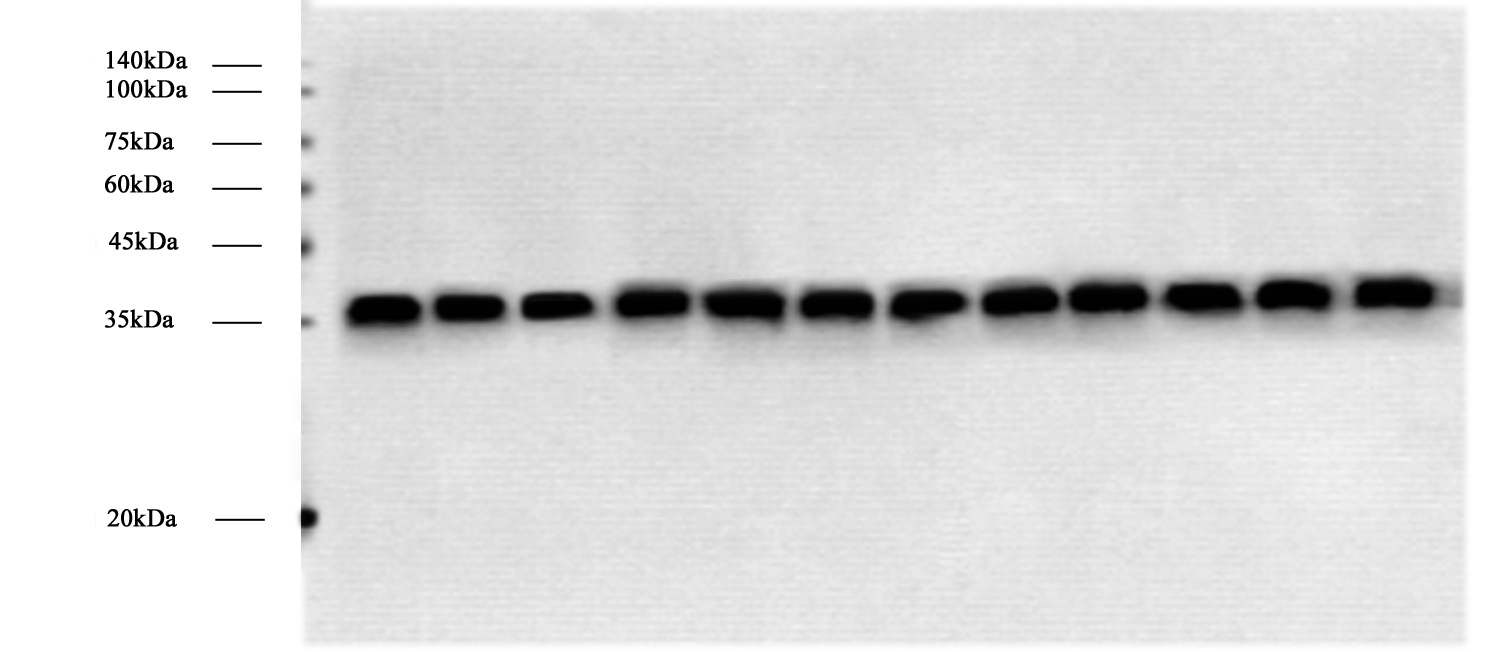

Supplement: Supplementary file 2 [file DataSheet3.ZIP › original data FIG4-7/Fig4B-C caspase3剪切/GAPDH-marker.tif]

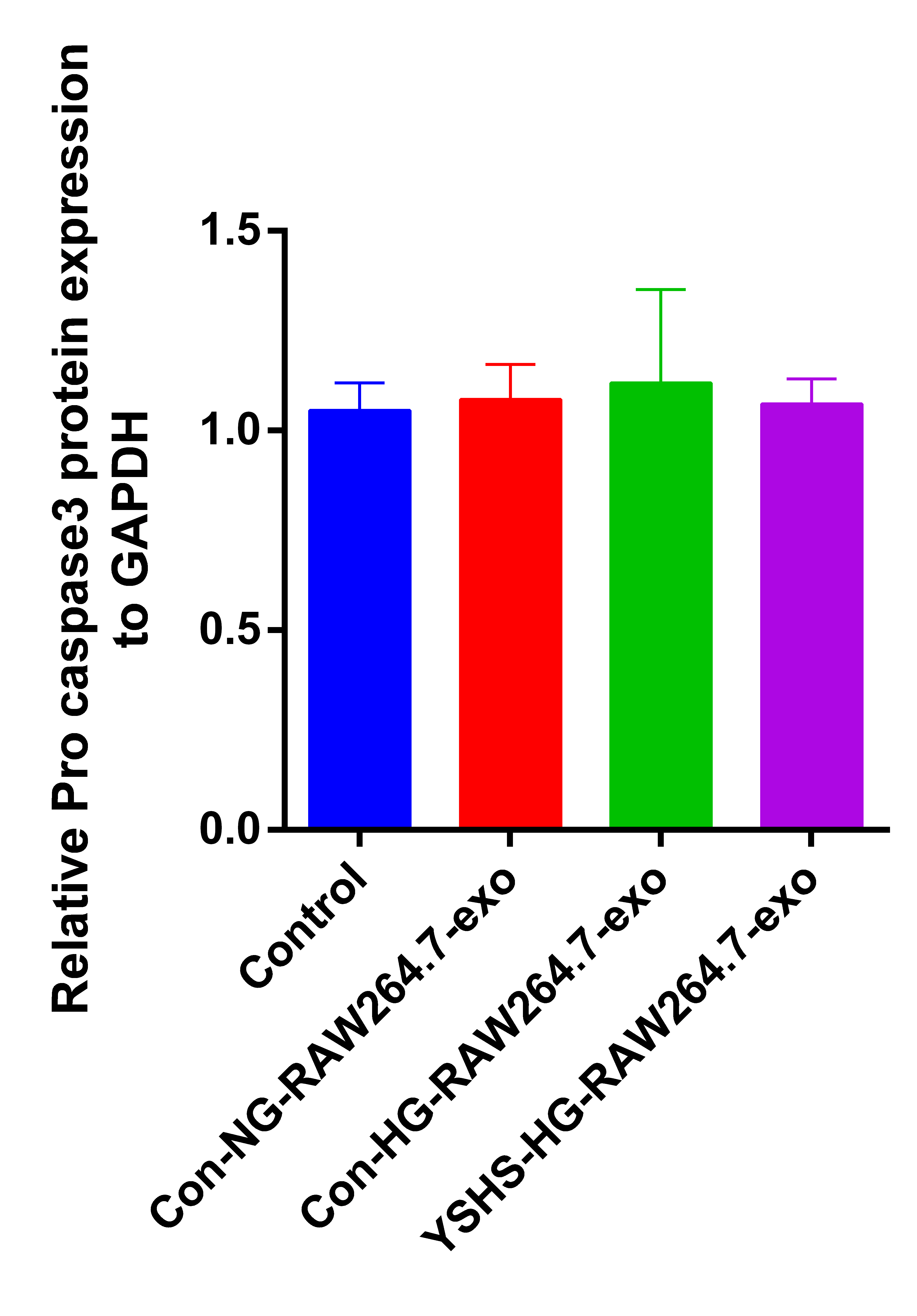

Supplement: Supplementary file 2 [file DataSheet3.ZIP › original data FIG4-7/Fig4B-C caspase3剪切/PRO.jpg]

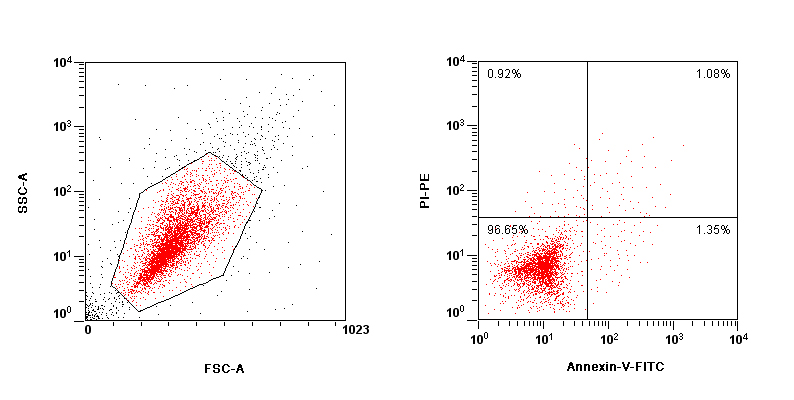

Supplement: Supplementary file 2 [file DataSheet3.ZIP › original data FIG4-7/Fig4D-E apoptosis/1-1.jpg]

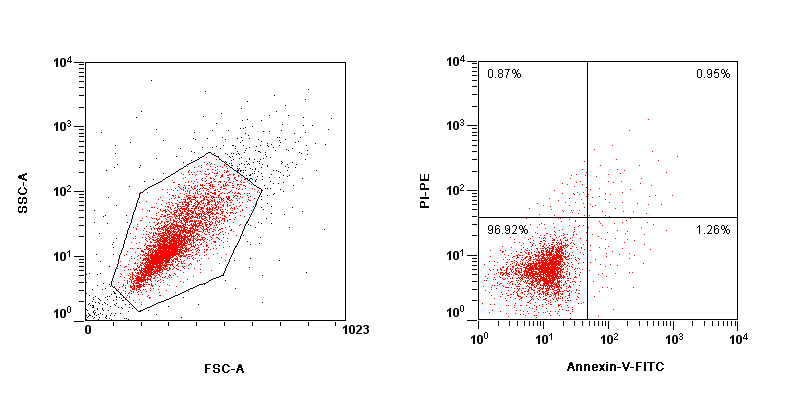

Supplement: Supplementary file 2 [file DataSheet3.ZIP › original data FIG4-7/Fig4D-E apoptosis/1-2.jpg]

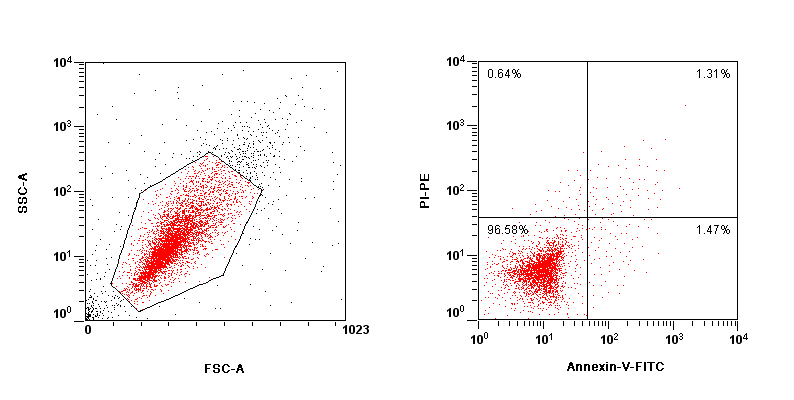

Supplement: Supplementary file 2 [file DataSheet3.ZIP › original data FIG4-7/Fig4D-E apoptosis/1-3.jpg]

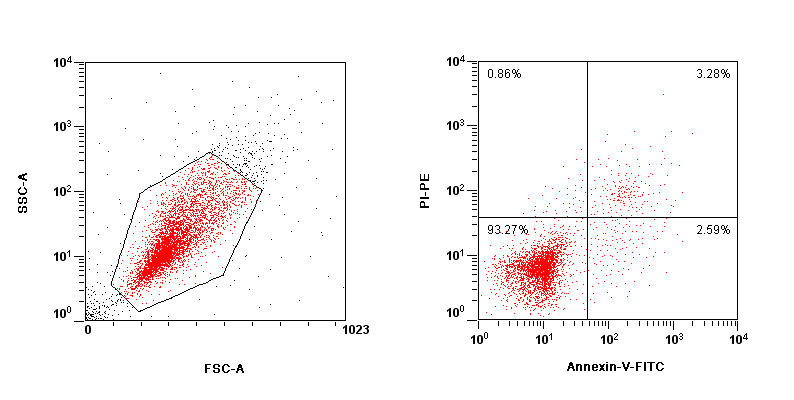

Supplement: Supplementary file 2 [file DataSheet3.ZIP › original data FIG4-7/Fig4D-E apoptosis/2-1.jpg]

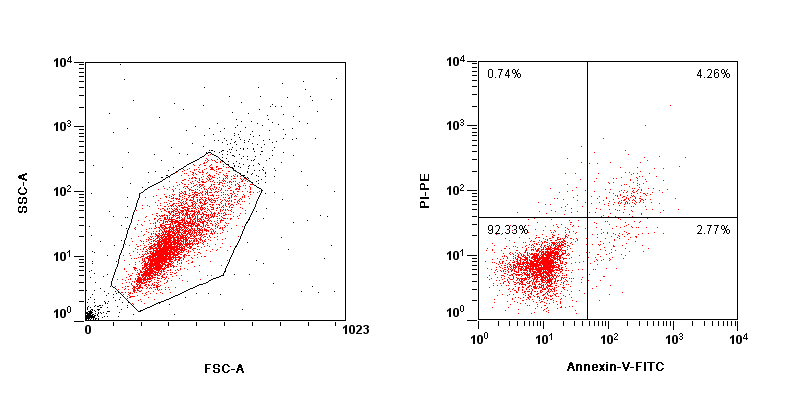

Supplement: Supplementary file 2 [file DataSheet3.ZIP › original data FIG4-7/Fig4D-E apoptosis/2-2.jpg]

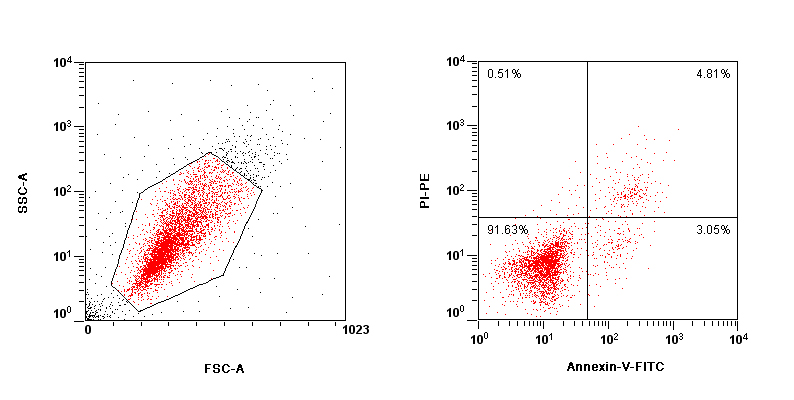

Supplement: Supplementary file 2 [file DataSheet3.ZIP › original data FIG4-7/Fig4D-E apoptosis/2-3.jpg]

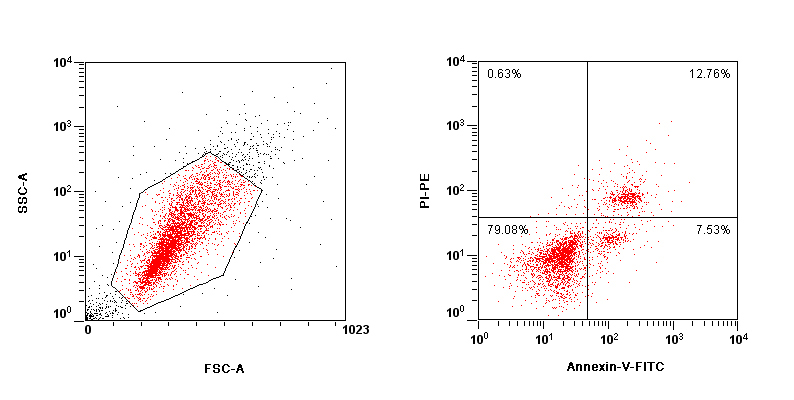

Supplement: Supplementary file 2 [file DataSheet3.ZIP › original data FIG4-7/Fig4D-E apoptosis/3-1.jpg]

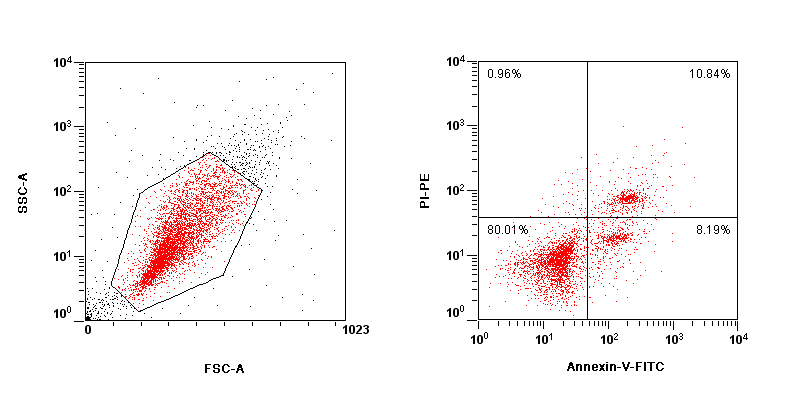

Supplement: Supplementary file 2 [file DataSheet3.ZIP › original data FIG4-7/Fig4D-E apoptosis/3-2.jpg]

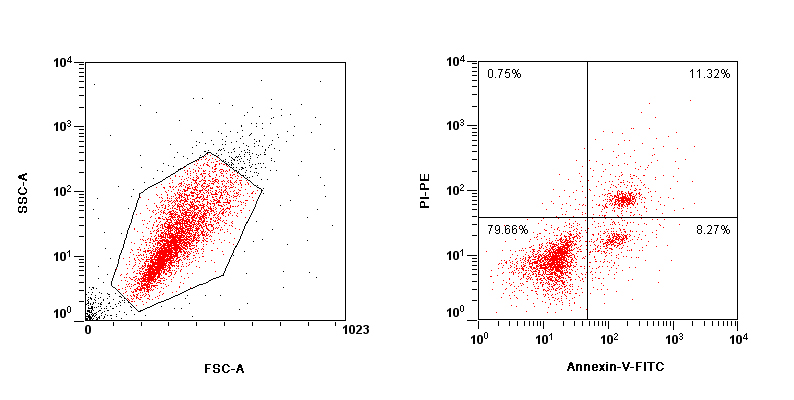

Supplement: Supplementary file 2 [file DataSheet3.ZIP › original data FIG4-7/Fig4D-E apoptosis/3-3.jpg]

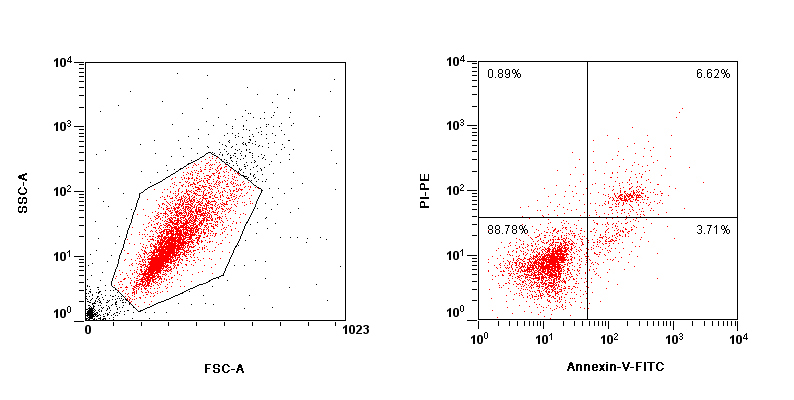

Supplement: Supplementary file 2 [file DataSheet3.ZIP › original data FIG4-7/Fig4D-E apoptosis/4-1.jpg]

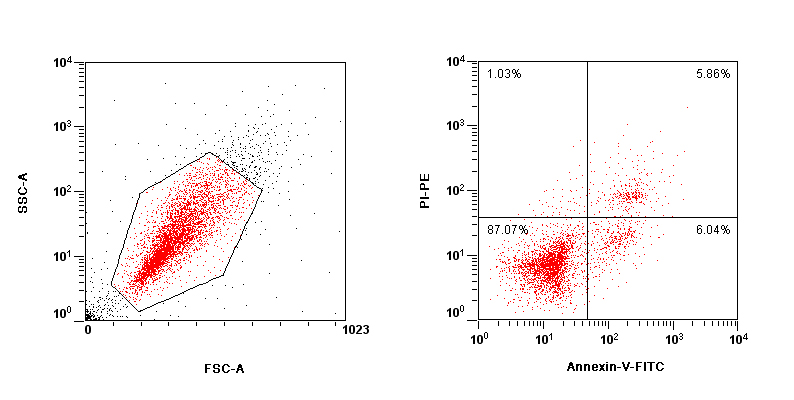

Supplement: Supplementary file 2 [file DataSheet3.ZIP › original data FIG4-7/Fig4D-E apoptosis/4-2.jpg]

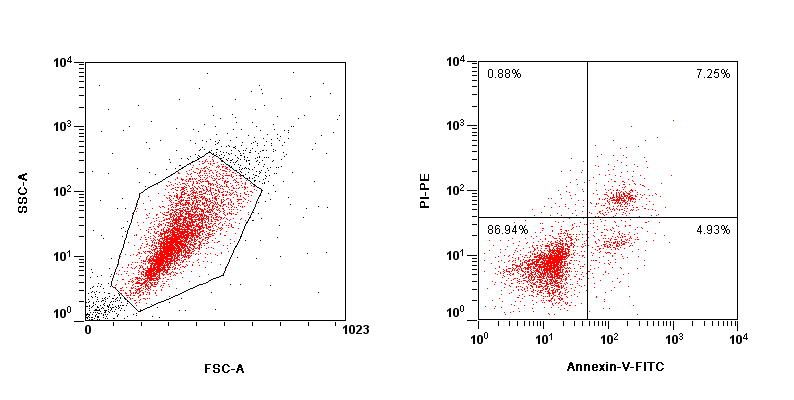

Supplement: Supplementary file 2 [file DataSheet3.ZIP › original data FIG4-7/Fig4D-E apoptosis/4-3.jpg]

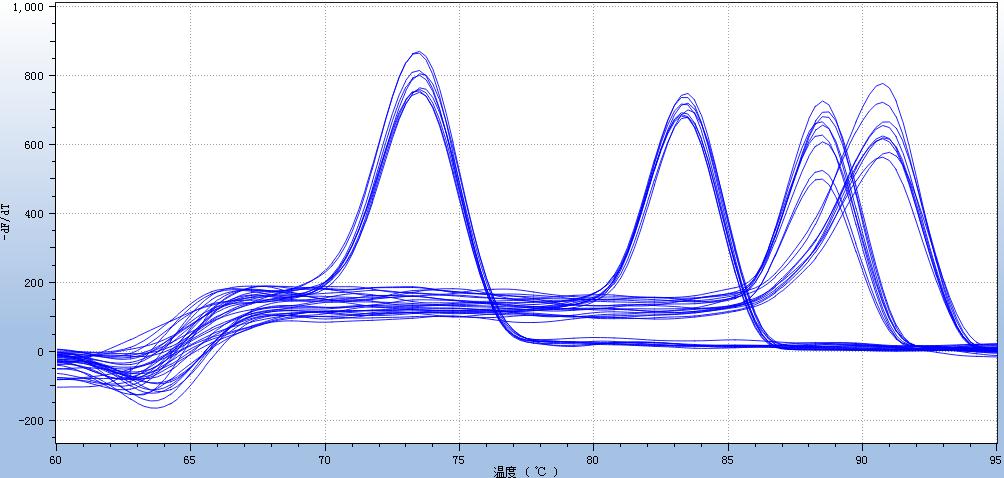

Supplement: Supplementary file 2 [file DataSheet3.ZIP › original data FIG4-7/Fig5A-C rt-pcr-mir/mir-.jpg]

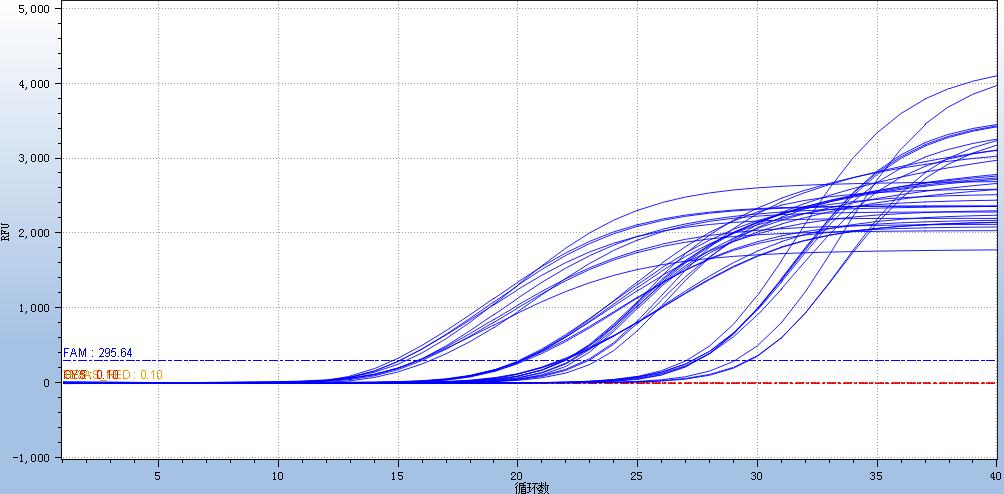

Supplement: Supplementary file 2 [file DataSheet3.ZIP › original data FIG4-7/Fig5A-C rt-pcr-mir/mir.jpg]

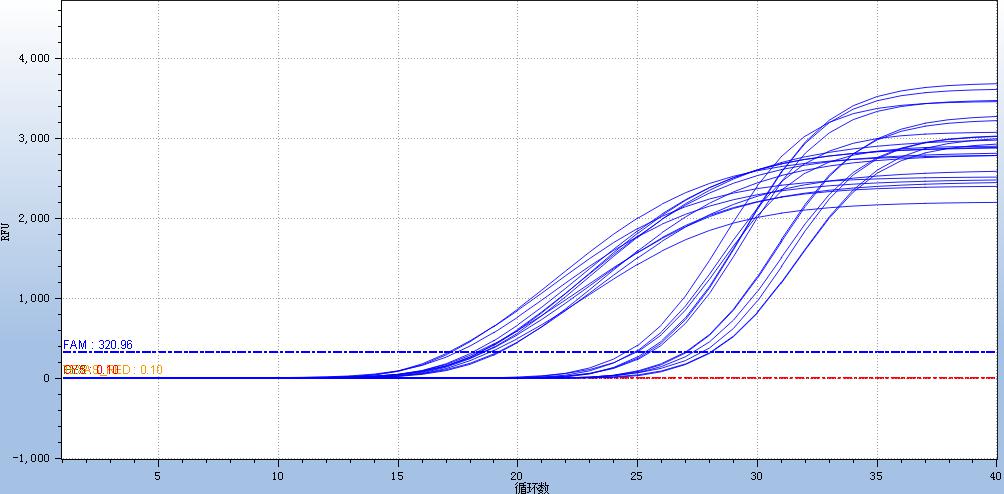

Supplement: Supplementary file 2 [file DataSheet3.ZIP › original data FIG4-7/Fig5D RT-PCR-miR/1.jpg]

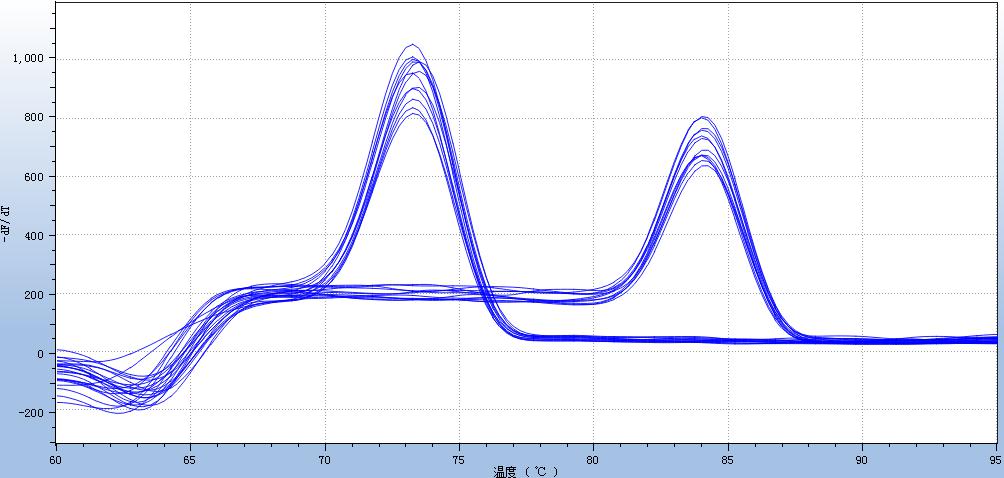

Supplement: Supplementary file 2 [file DataSheet3.ZIP › original data FIG4-7/Fig5D RT-PCR-miR/2.jpg]

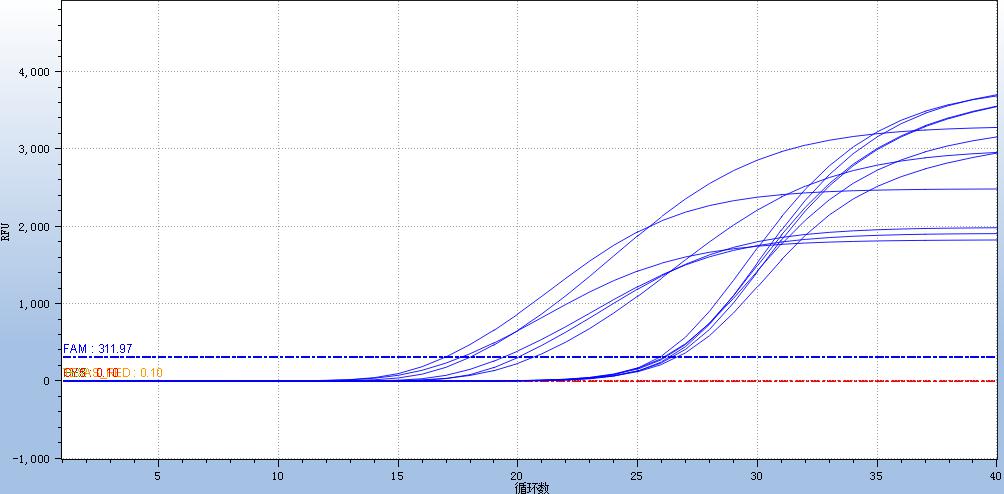

Supplement: Supplementary file 2 [file DataSheet3.ZIP › original data FIG4-7/Fig6A RT-PCR-miR/1.jpg]

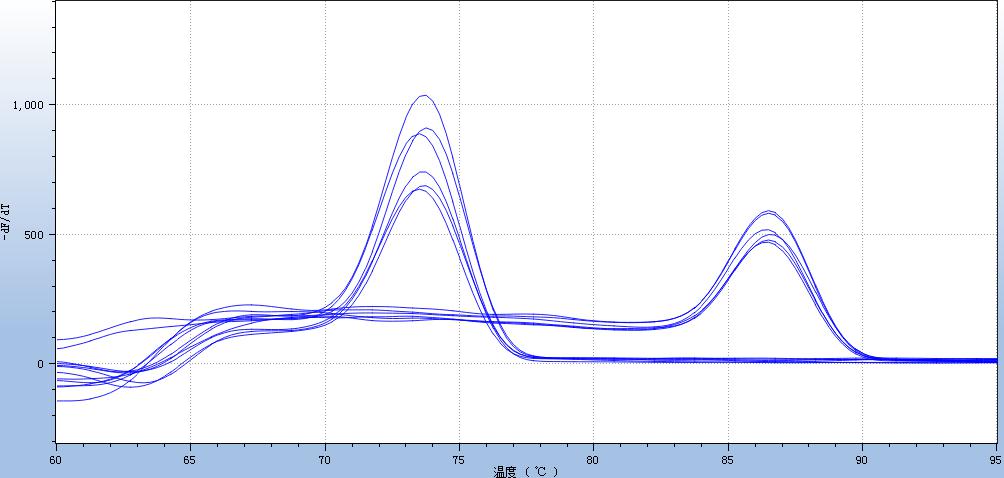

Supplement: Supplementary file 2 [file DataSheet3.ZIP › original data FIG4-7/Fig6A RT-PCR-miR/2.jpg]

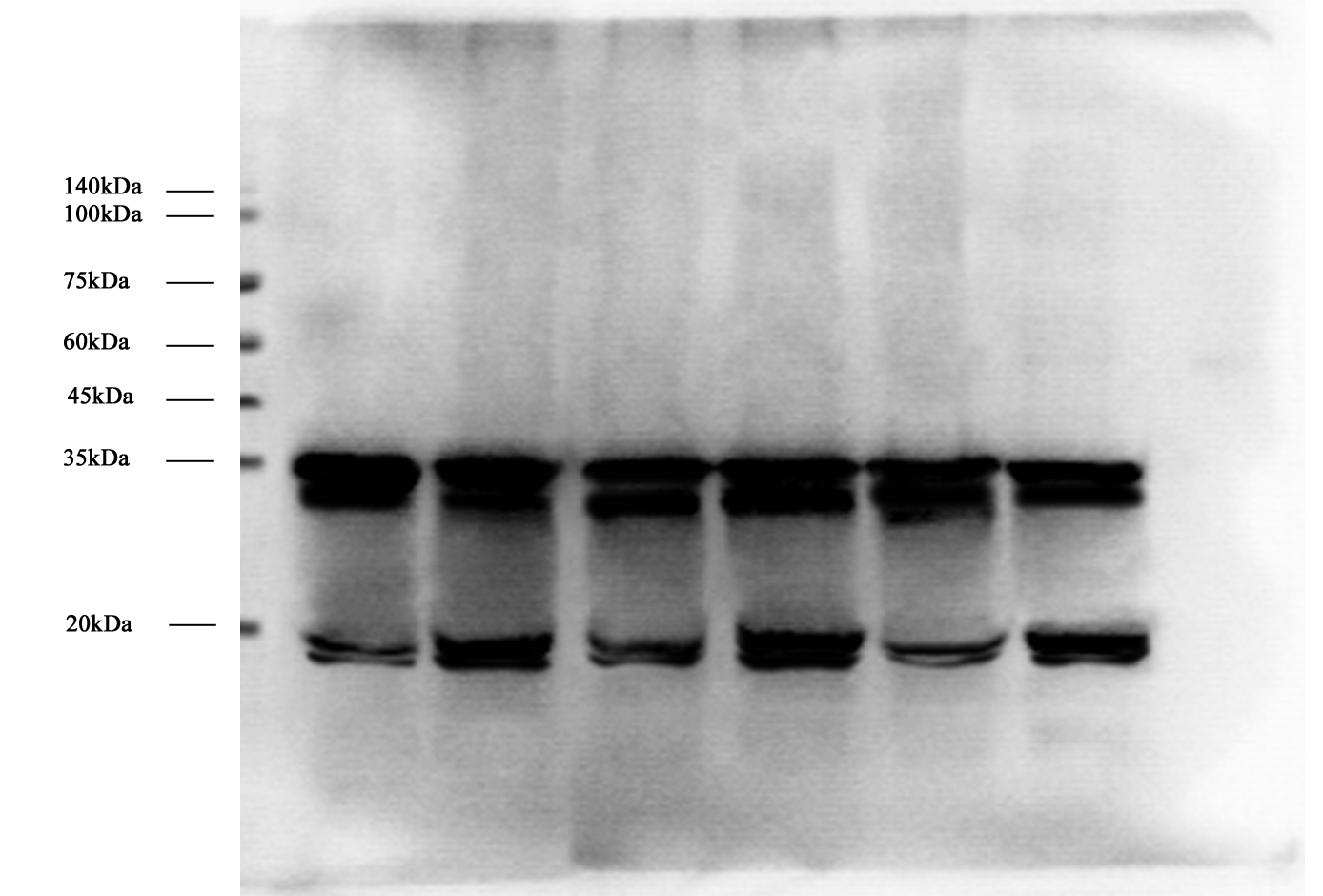

Supplement: Supplementary file 2 [file DataSheet3.ZIP › original data FIG4-7/Fig6C-D caspase3剪切/caspase3-marker.tif]

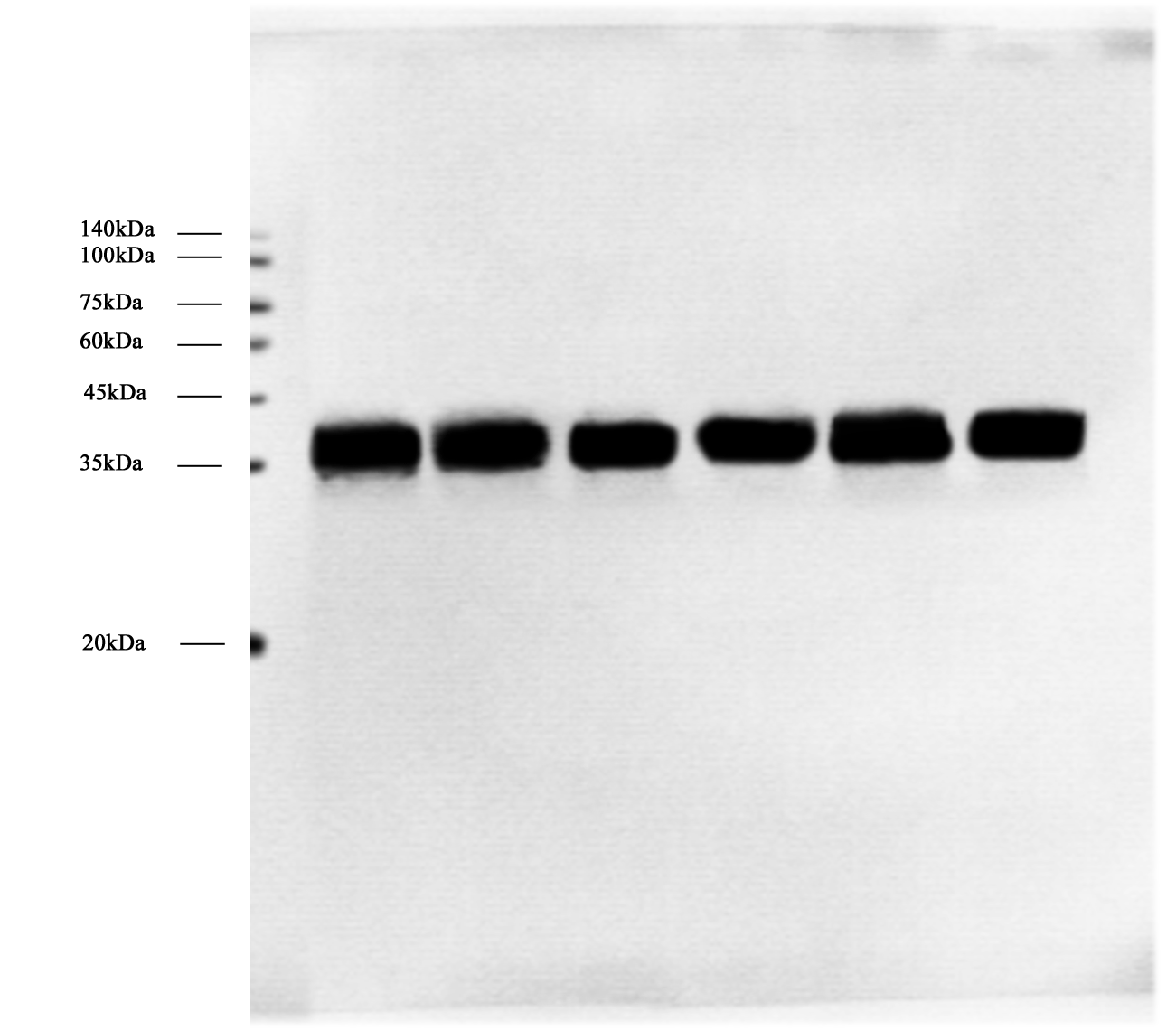

Supplement: Supplementary file 2 [file DataSheet3.ZIP › original data FIG4-7/Fig6C-D caspase3剪切/GAPDH-marker.tif]

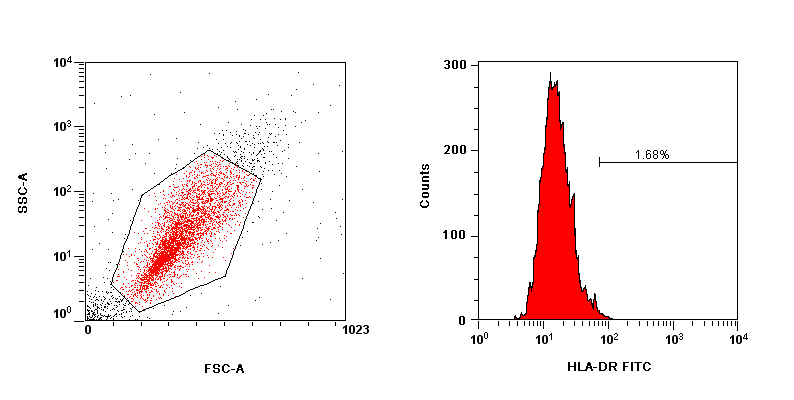

Supplement: Supplementary file 2 [file DataSheet3.ZIP › original data FIG4-7/Fig7A-B flow files/1-1.jpg]

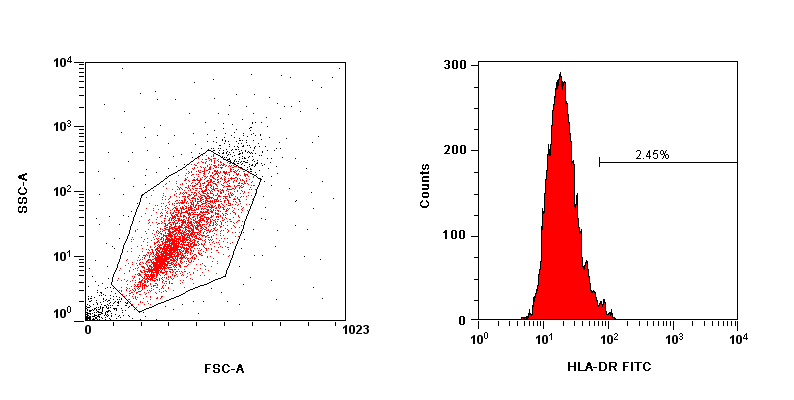

Supplement: Supplementary file 2 [file DataSheet3.ZIP › original data FIG4-7/Fig7A-B flow files/1-2.jpg]

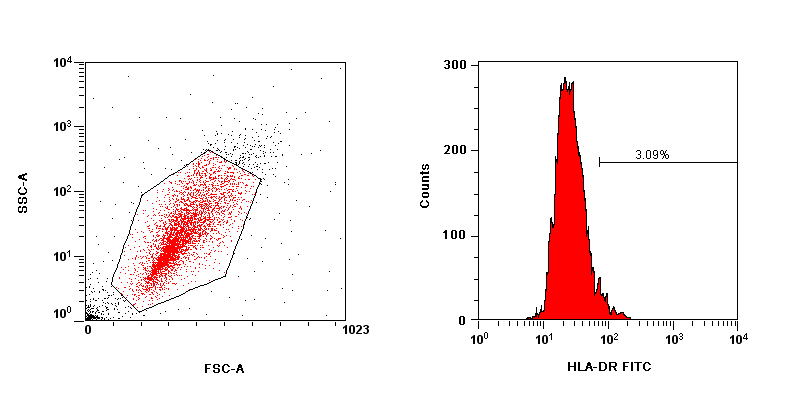

Supplement: Supplementary file 2 [file DataSheet3.ZIP › original data FIG4-7/Fig7A-B flow files/1-3.jpg]

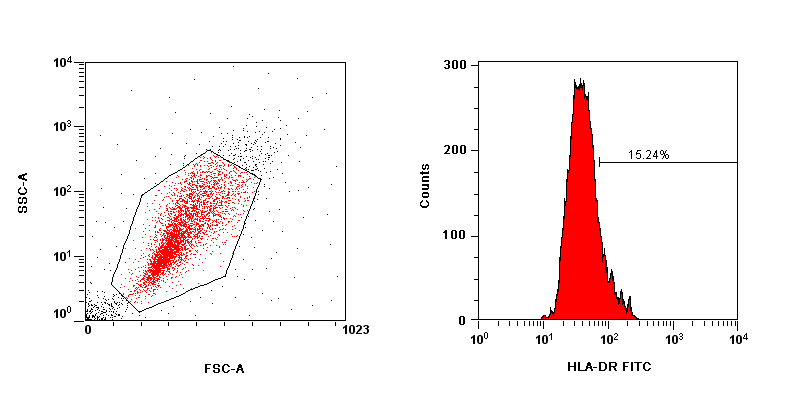

Supplement: Supplementary file 2 [file DataSheet3.ZIP › original data FIG4-7/Fig7A-B flow files/2-1.jpg]

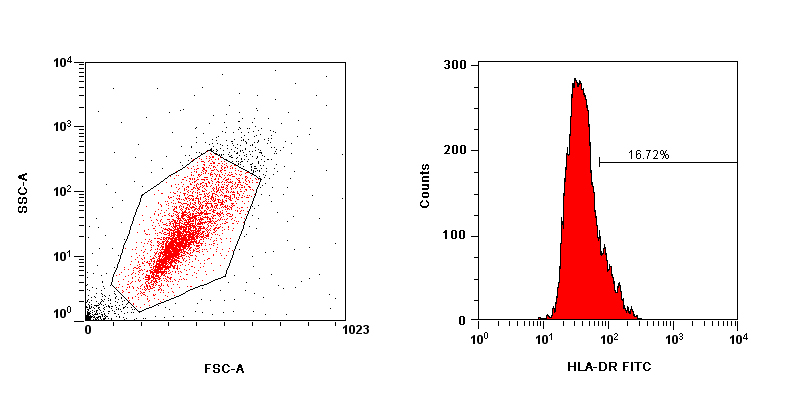

Supplement: Supplementary file 2 [file DataSheet3.ZIP › original data FIG4-7/Fig7A-B flow files/2-2.jpg]

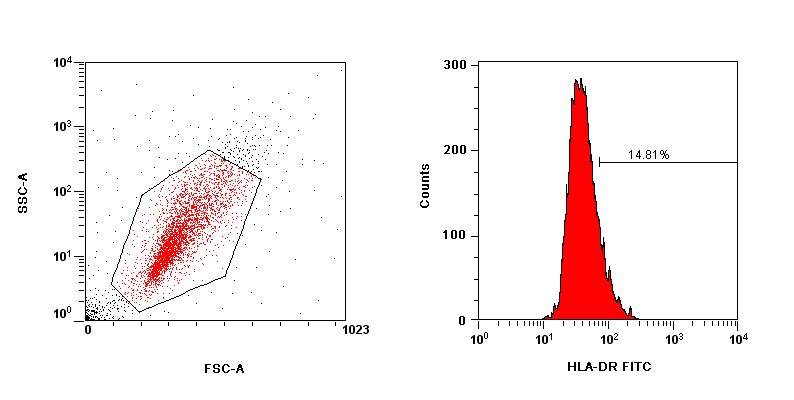

Supplement: Supplementary file 2 [file DataSheet3.ZIP › original data FIG4-7/Fig7A-B flow files/2-3.jpg]

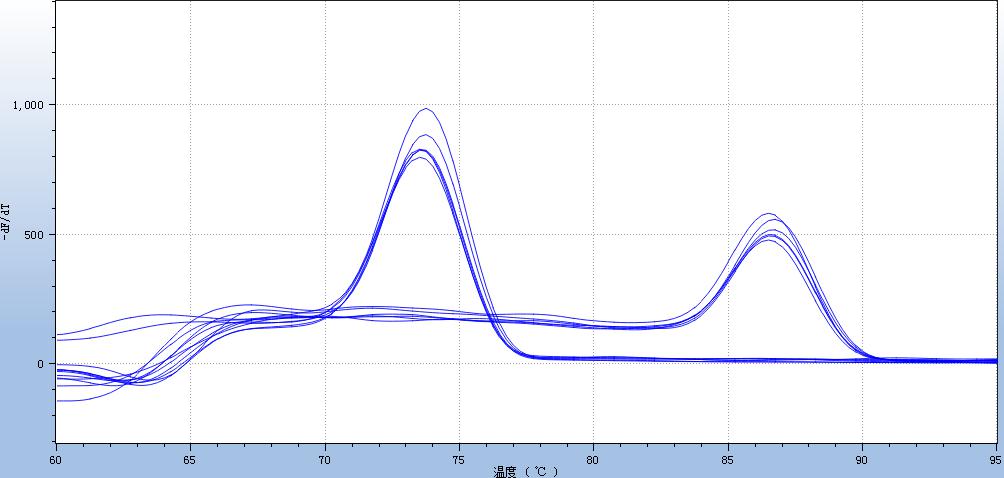

Supplement: Supplementary file 2 [file DataSheet3.ZIP › original data FIG4-7/Fig7C RT-PCR-miR/1-1.jpg]

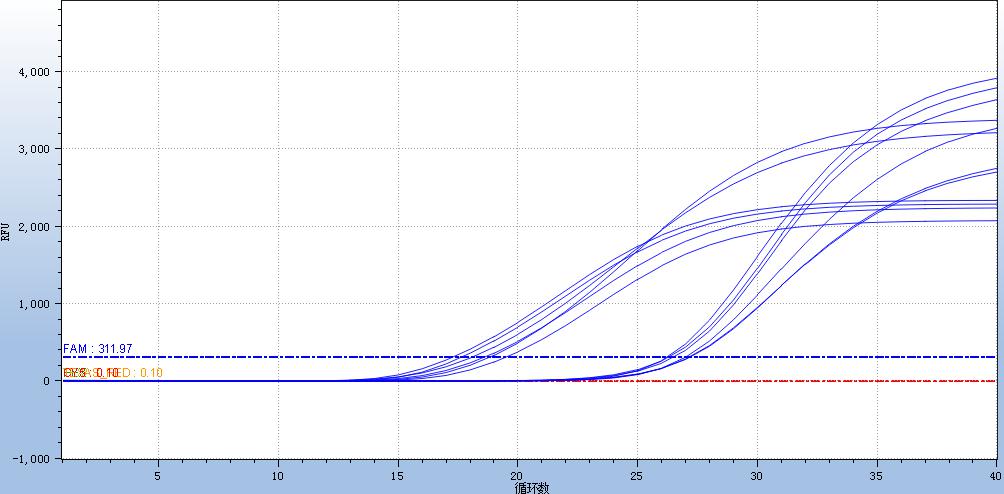

Supplement: Supplementary file 2 [file DataSheet3.ZIP › original data FIG4-7/Fig7C RT-PCR-miR/1.jpg]

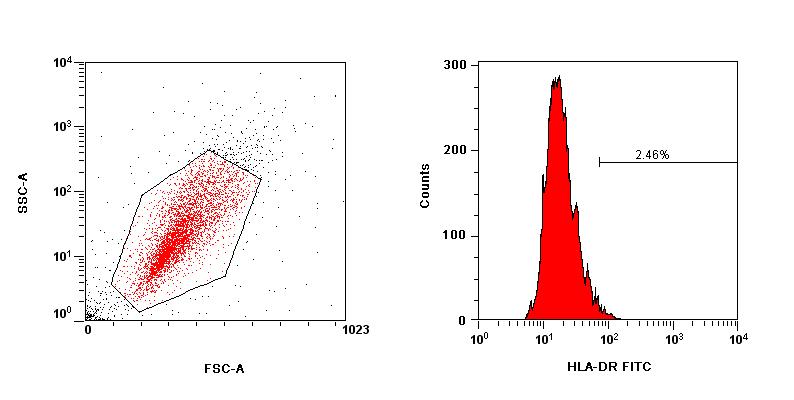

Supplement: Supplementary file 2 [file DataSheet3.ZIP › original data FIG4-7/Fig7D-E flow files/1-1.jpg]

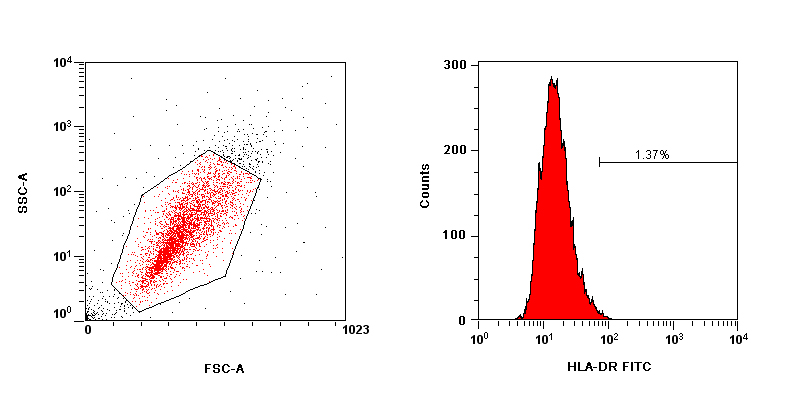

Supplement: Supplementary file 2 [file DataSheet3.ZIP › original data FIG4-7/Fig7D-E flow files/1-2 .jpg]

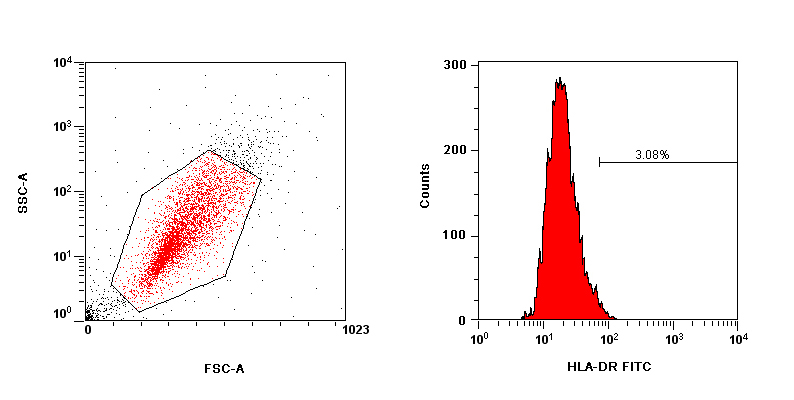

Supplement: Supplementary file 2 [file DataSheet3.ZIP › original data FIG4-7/Fig7D-E flow files/1-3 .jpg]

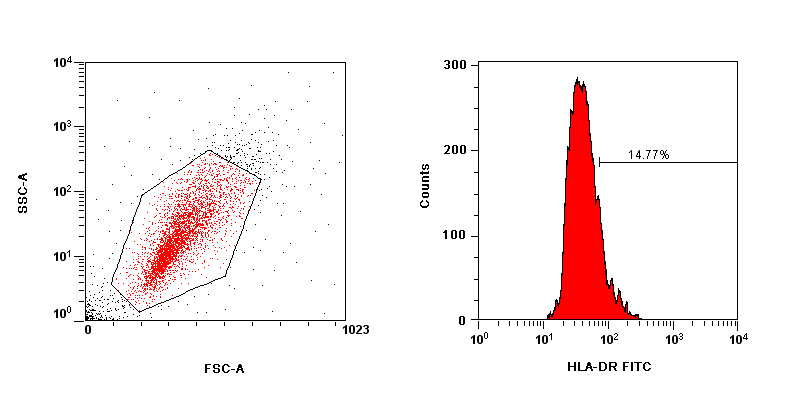

Supplement: Supplementary file 2 [file DataSheet3.ZIP › original data FIG4-7/Fig7D-E flow files/2-1.jpg]

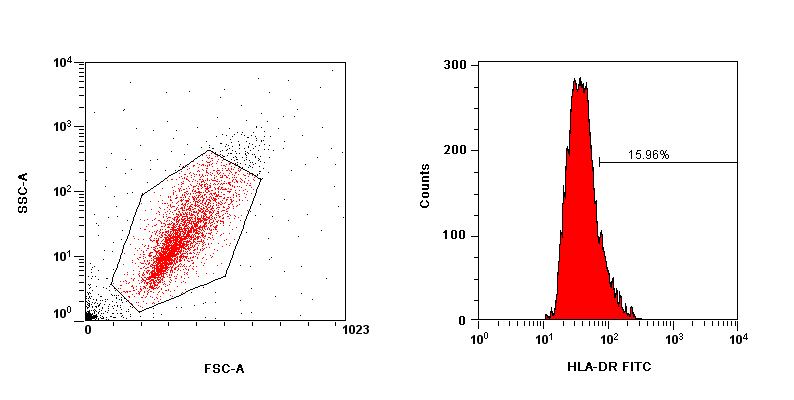

Supplement: Supplementary file 2 [file DataSheet3.ZIP › original data FIG4-7/Fig7D-E flow files/2-2 .jpg]

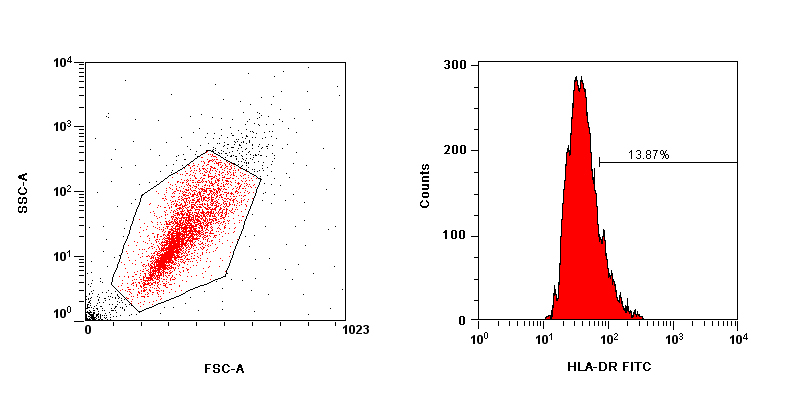

Supplement: Supplementary file 2 [file DataSheet3.ZIP › original data FIG4-7/Fig7D-E flow files/2-3.jpg]

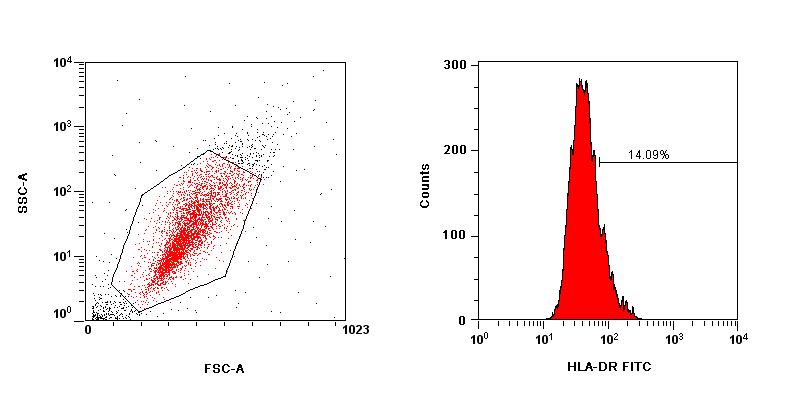

Supplement: Supplementary file 2 [file DataSheet3.ZIP › original data FIG4-7/Fig7D-E flow files/3-1.jpg]

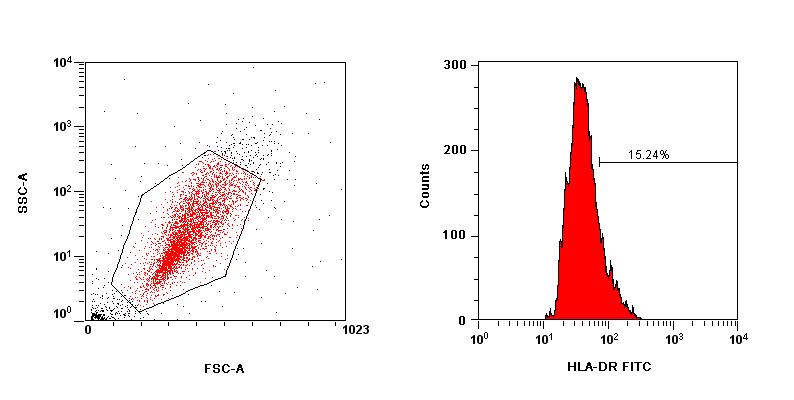

Supplement: Supplementary file 2 [file DataSheet3.ZIP › original data FIG4-7/Fig7D-E flow files/3-2.jpg]

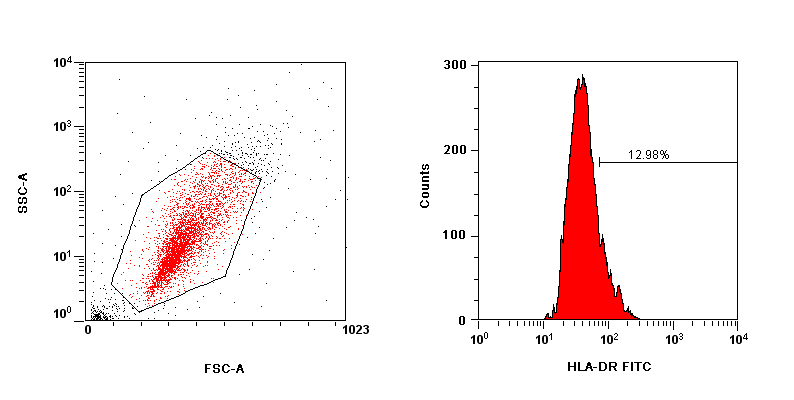

Supplement: Supplementary file 2 [file DataSheet3.ZIP › original data FIG4-7/Fig7D-E flow files/3-3.jpg]

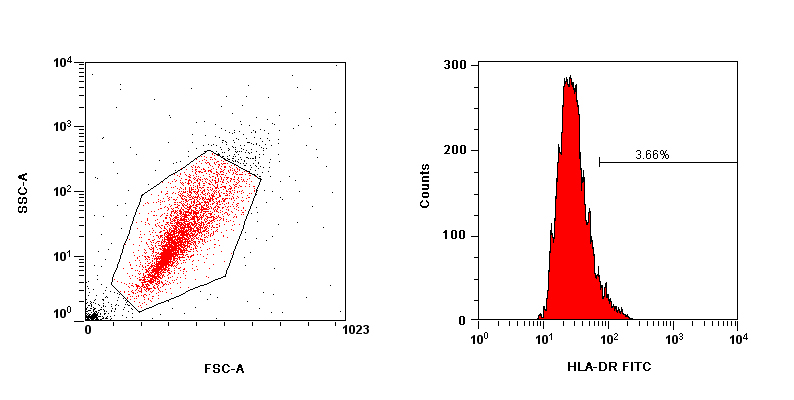

Supplement: Supplementary file 2 [file DataSheet3.ZIP › original data FIG4-7/Fig7D-E flow files/4-1.jpg]

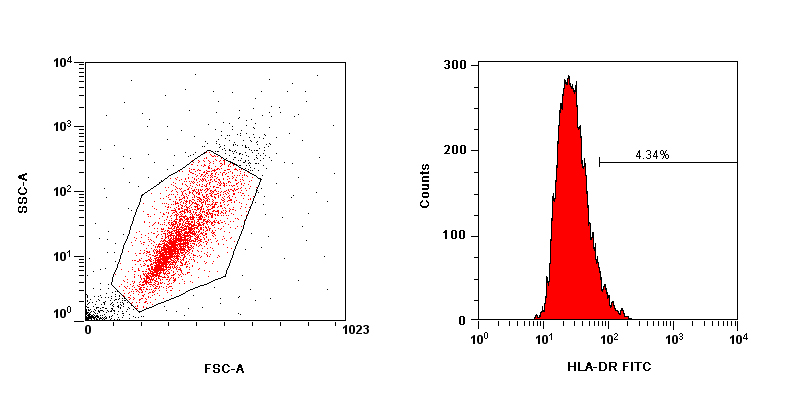

Supplement: Supplementary file 2 [file DataSheet3.ZIP › original data FIG4-7/Fig7D-E flow files/4-2 .jpg]

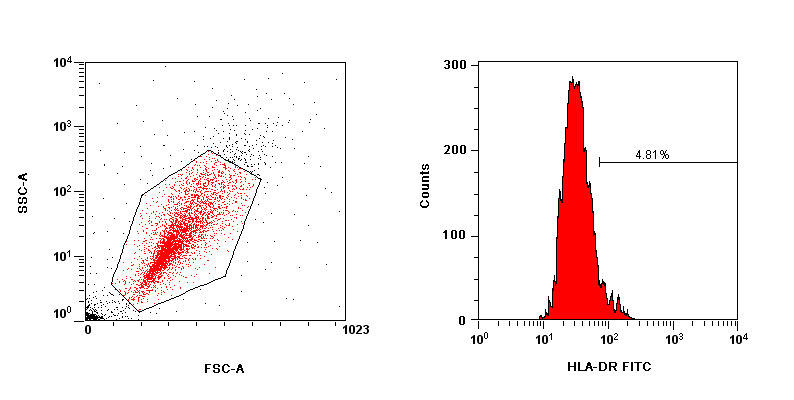

Supplement: Supplementary file 2 [file DataSheet3.ZIP › original data FIG4-7/Fig7D-E flow files/4-3.jpg]

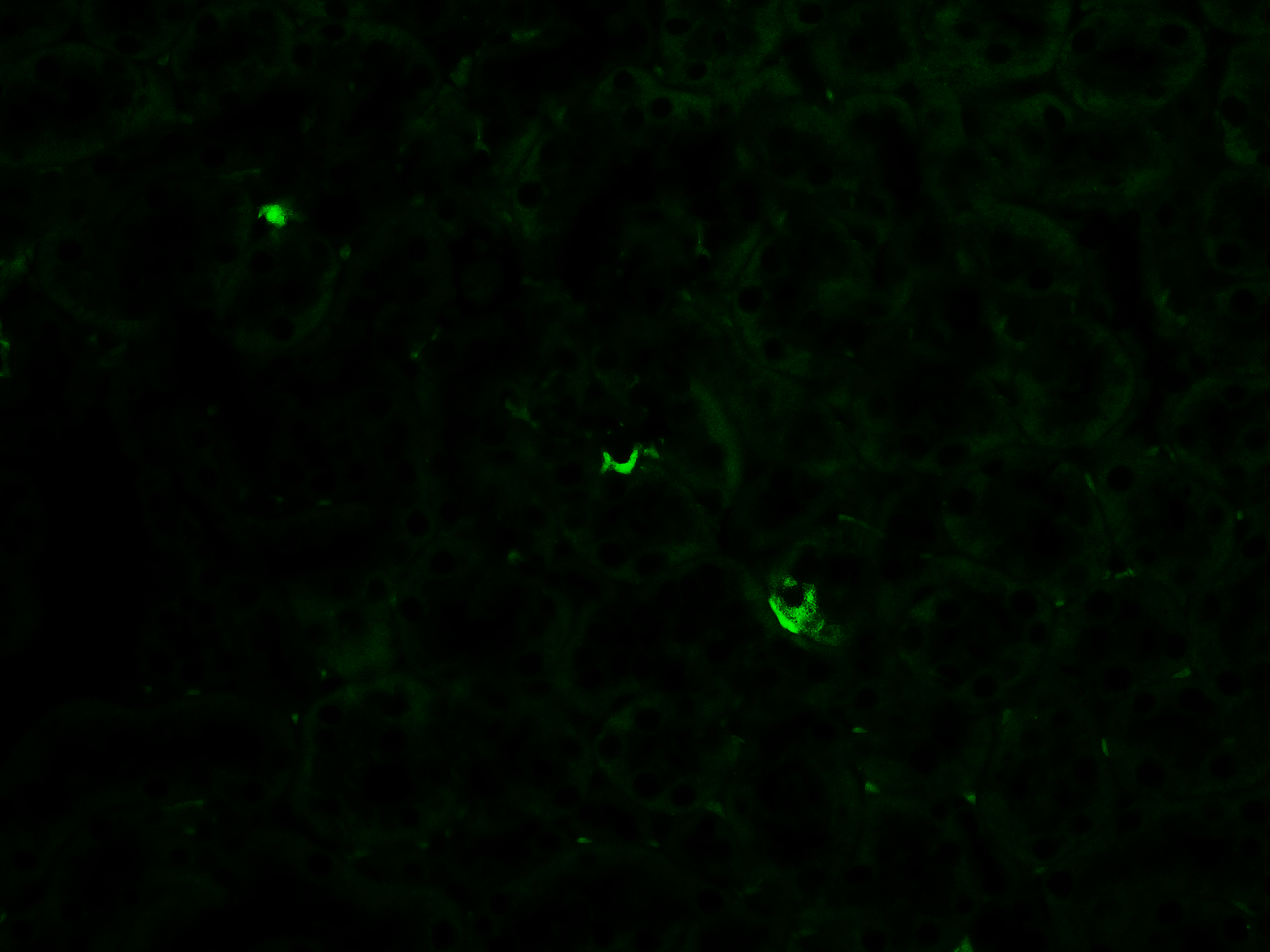

Supplement: Supplementary file 4 [file DataSheet4.ZIP › original data FIG8(I)/HLA-DR-1/1-1(400倍).tif]

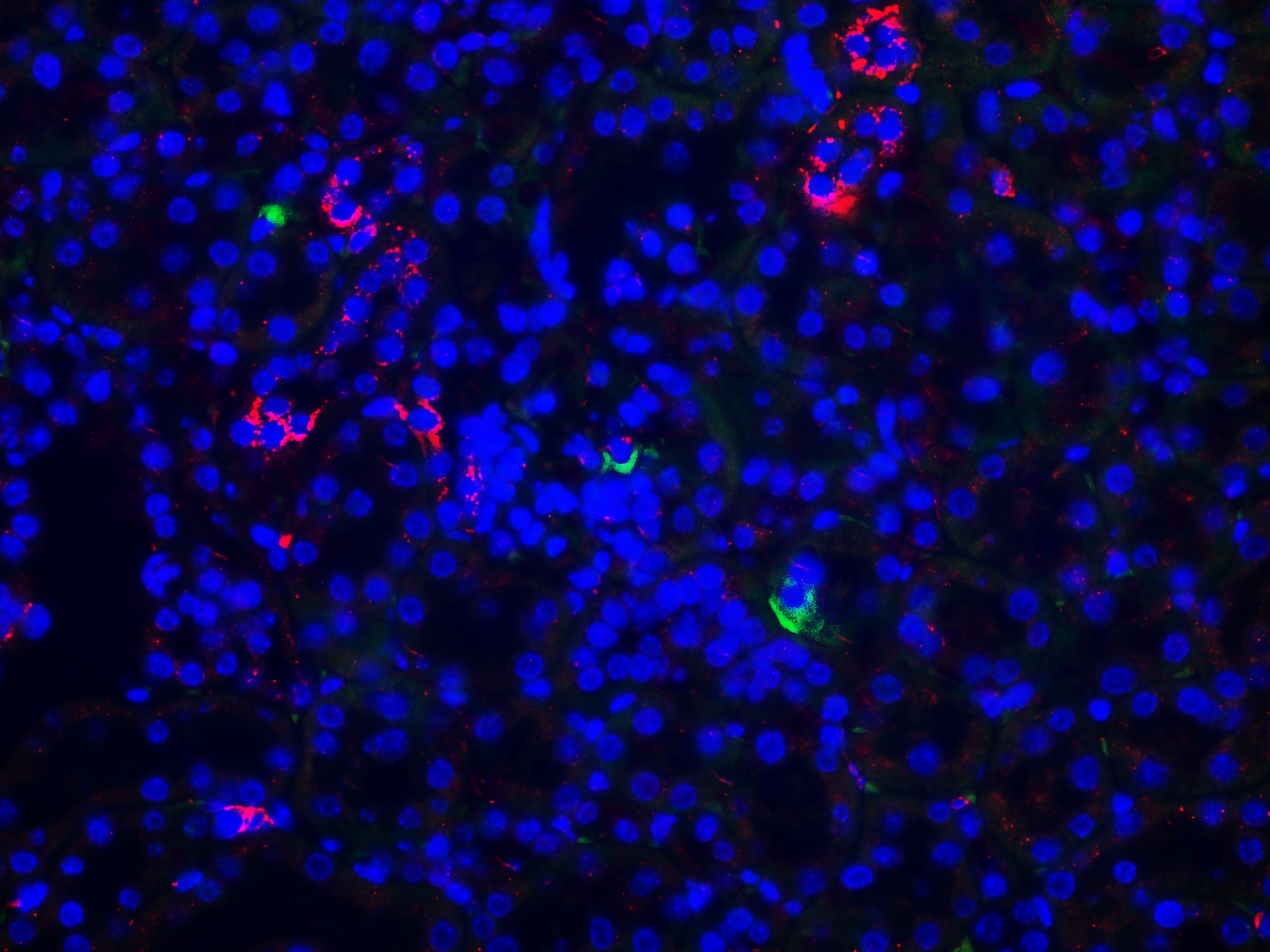

Supplement: Supplementary file 4 [file DataSheet4.ZIP › original data FIG8(I)/HLA-DR-1/1-2(400倍)-merge.tif]

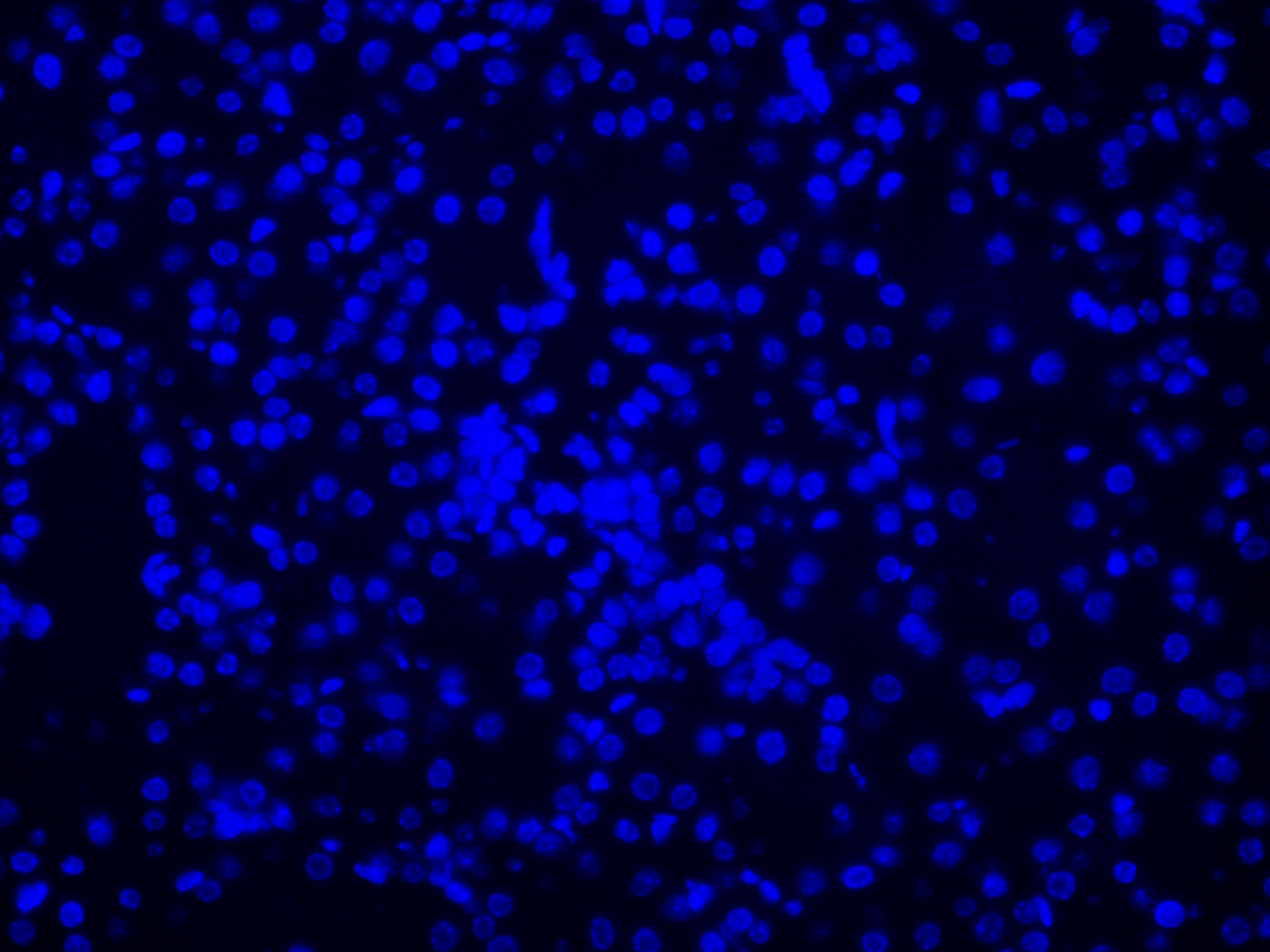

Supplement: Supplementary file 4 [file DataSheet4.ZIP › original data FIG8(I)/HLA-DR-1/1-2(400倍).tif]

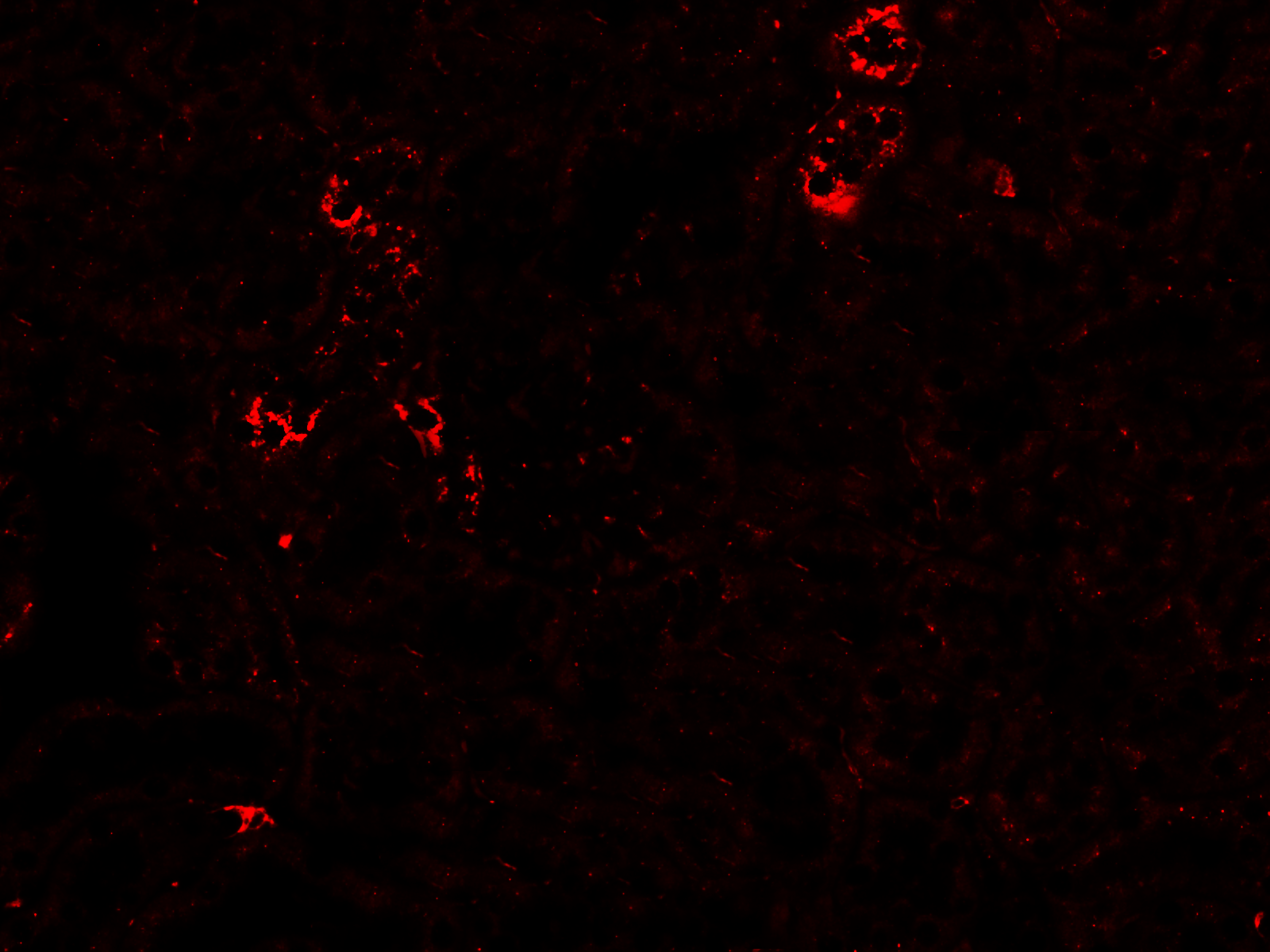

Supplement: Supplementary file 4 [file DataSheet4.ZIP › original data FIG8(I)/HLA-DR-1/1-3(400倍).tif]

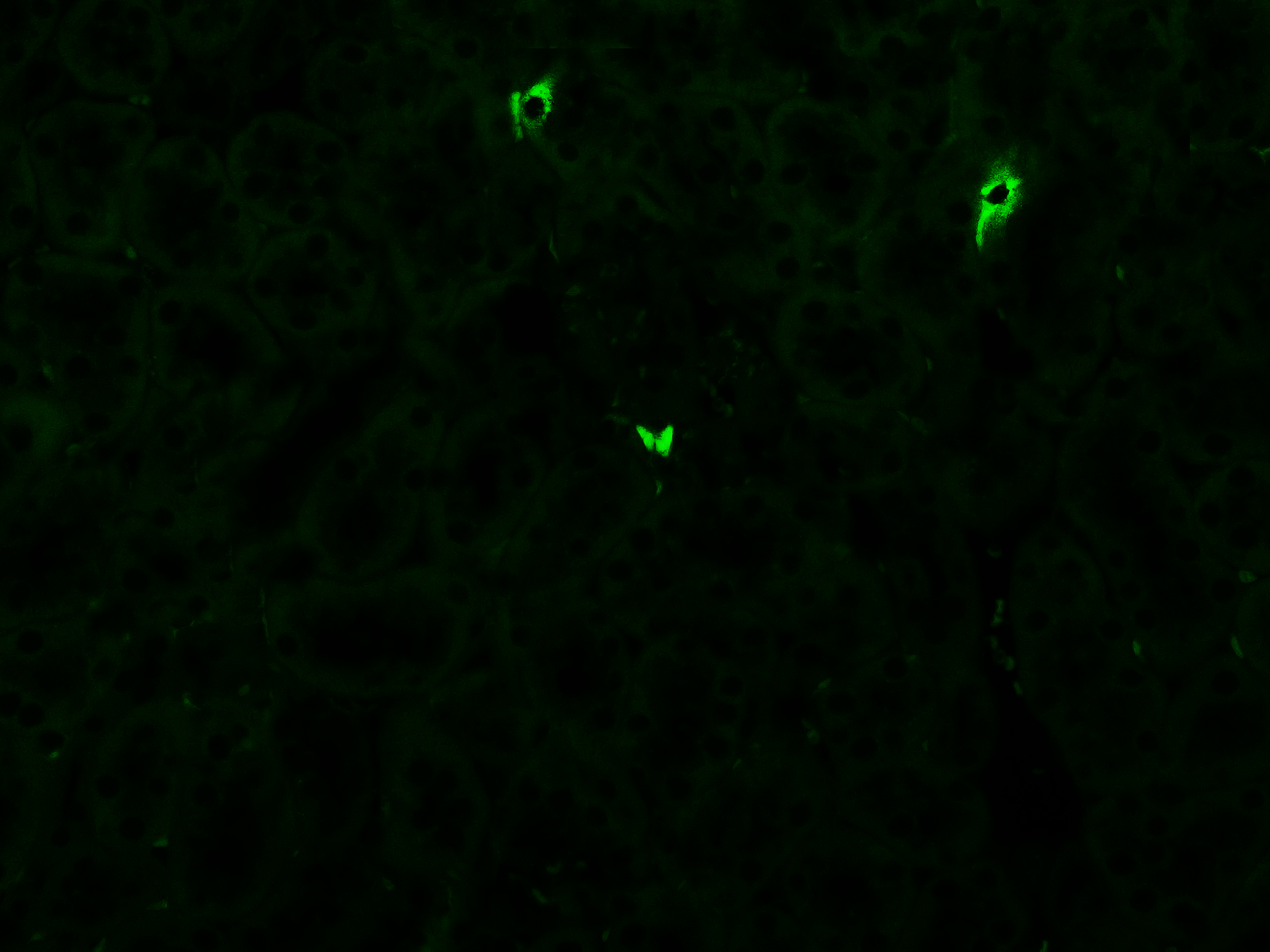

Supplement: Supplementary file 4 [file DataSheet4.ZIP › original data FIG8(I)/HLA-DR-1/1-4(400倍).tif]

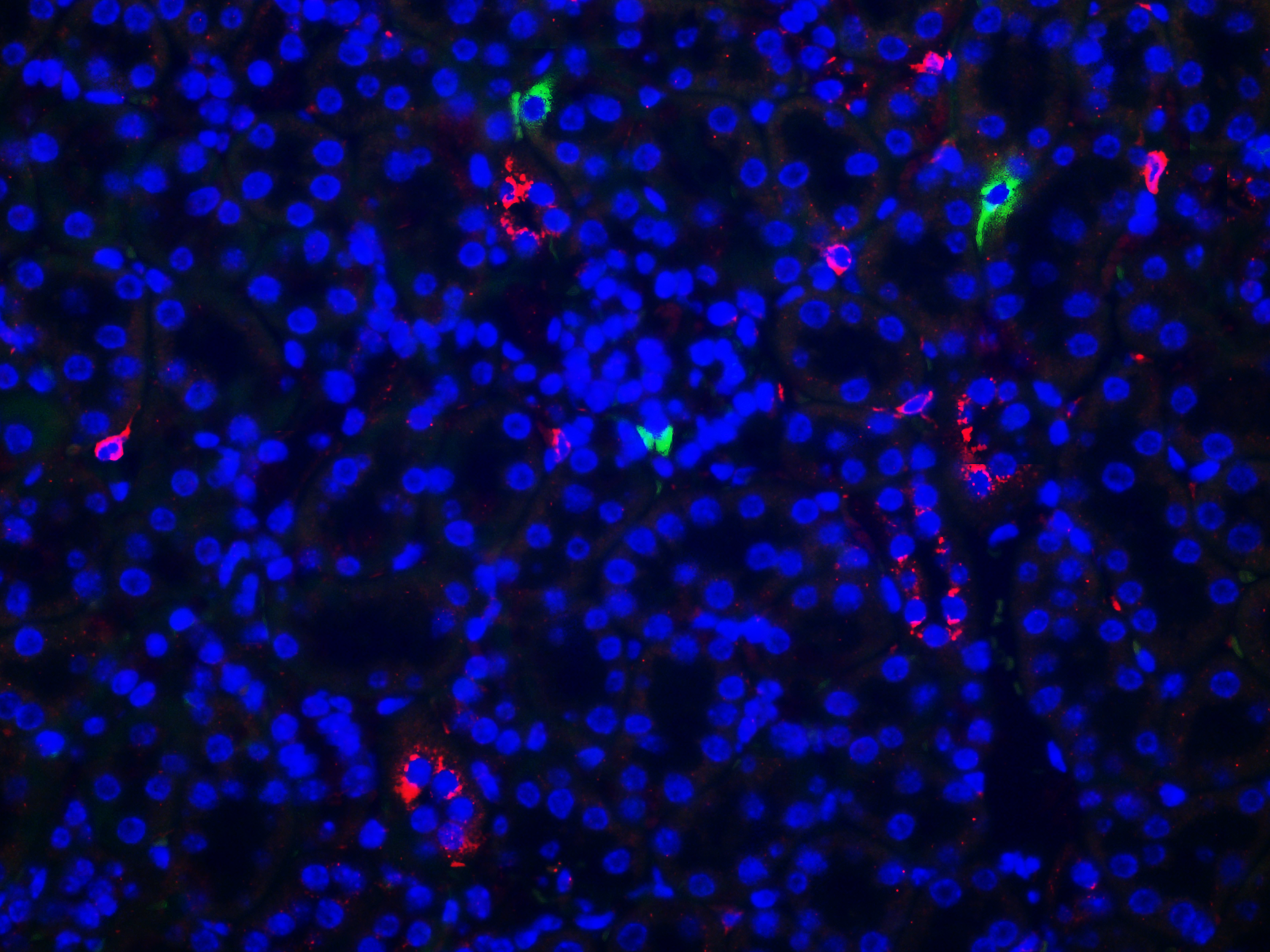

Supplement: Supplementary file 4 [file DataSheet4.ZIP › original data FIG8(I)/HLA-DR-1/1-5(400倍)-merge.tif]

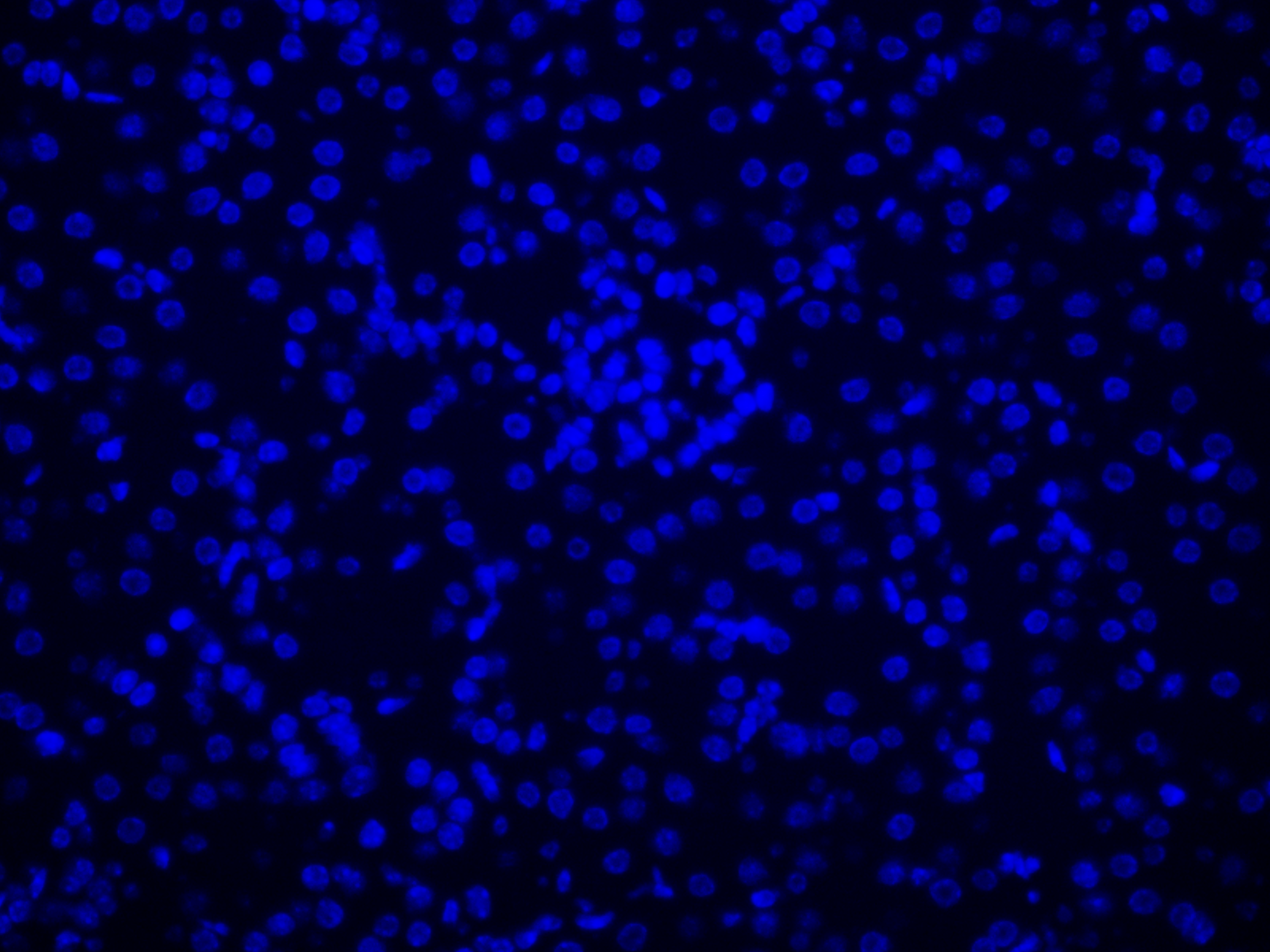

Supplement: Supplementary file 4 [file DataSheet4.ZIP › original data FIG8(I)/HLA-DR-1/1-5(400倍).tif]

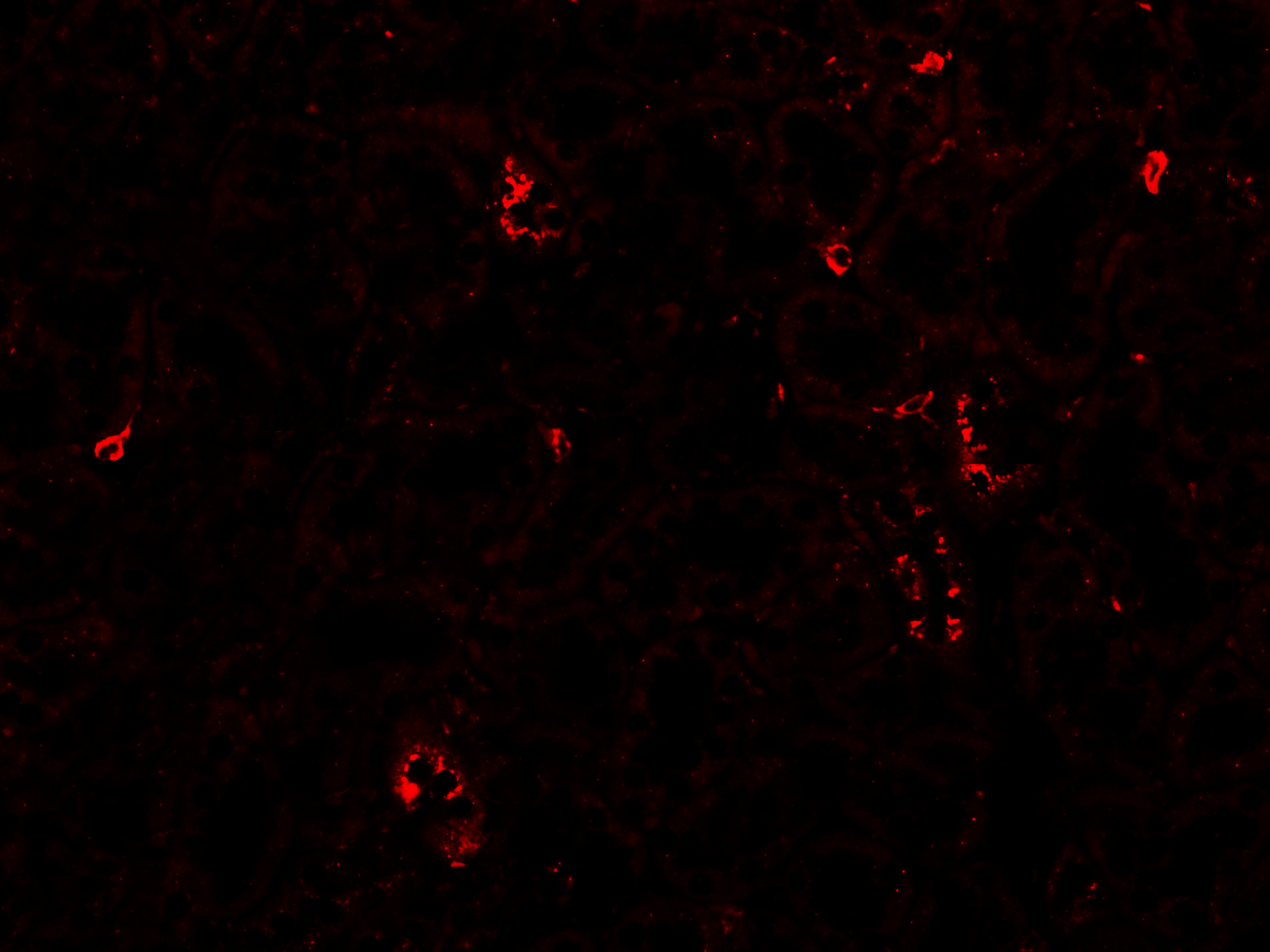

Supplement: Supplementary file 4 [file DataSheet4.ZIP › original data FIG8(I)/HLA-DR-1/1-6(400倍).tif]

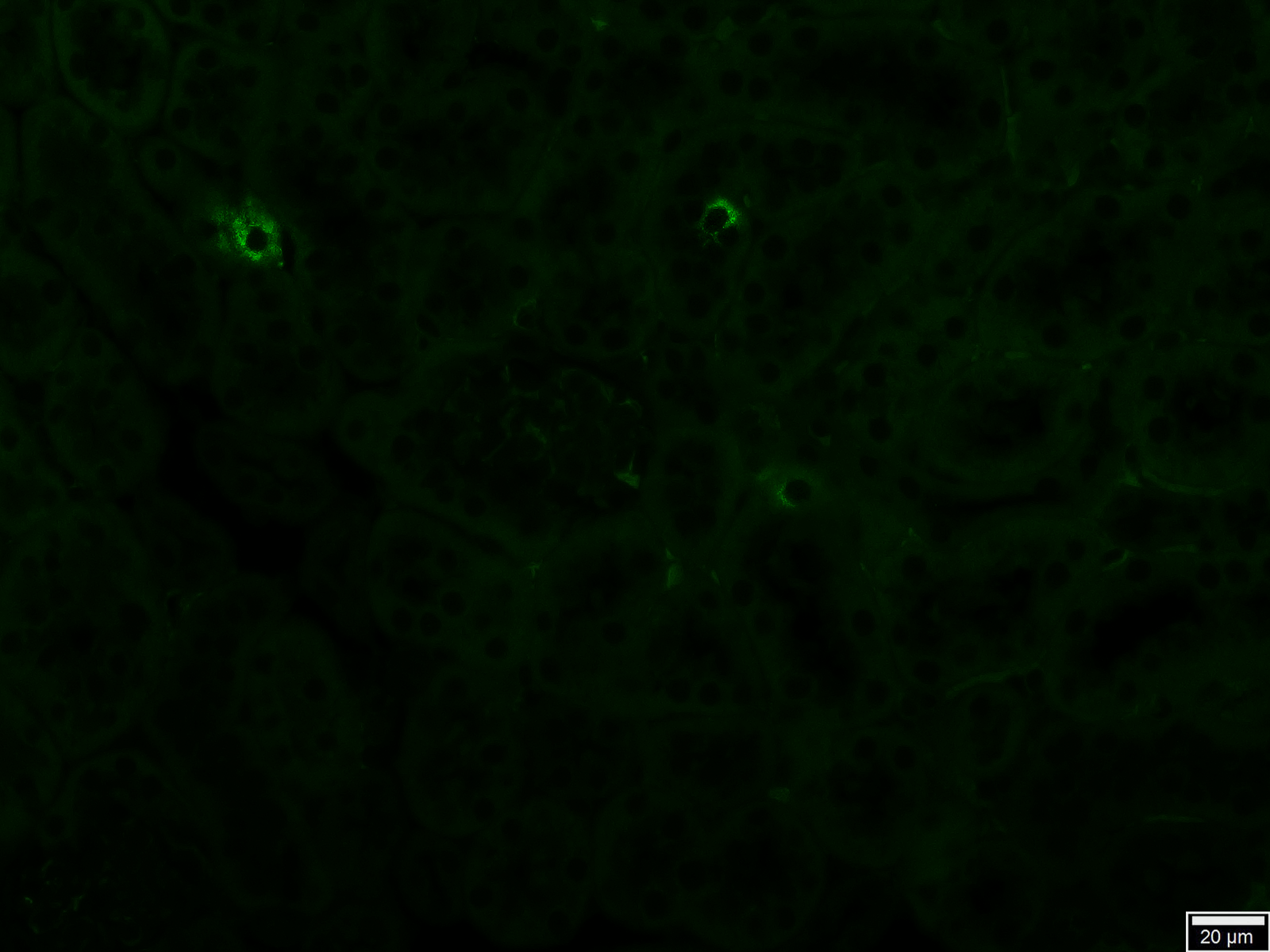

Supplement: Supplementary file 4 [file DataSheet4.ZIP › original data FIG8(I)/HLA-DR-1/1-7(400倍).tif]

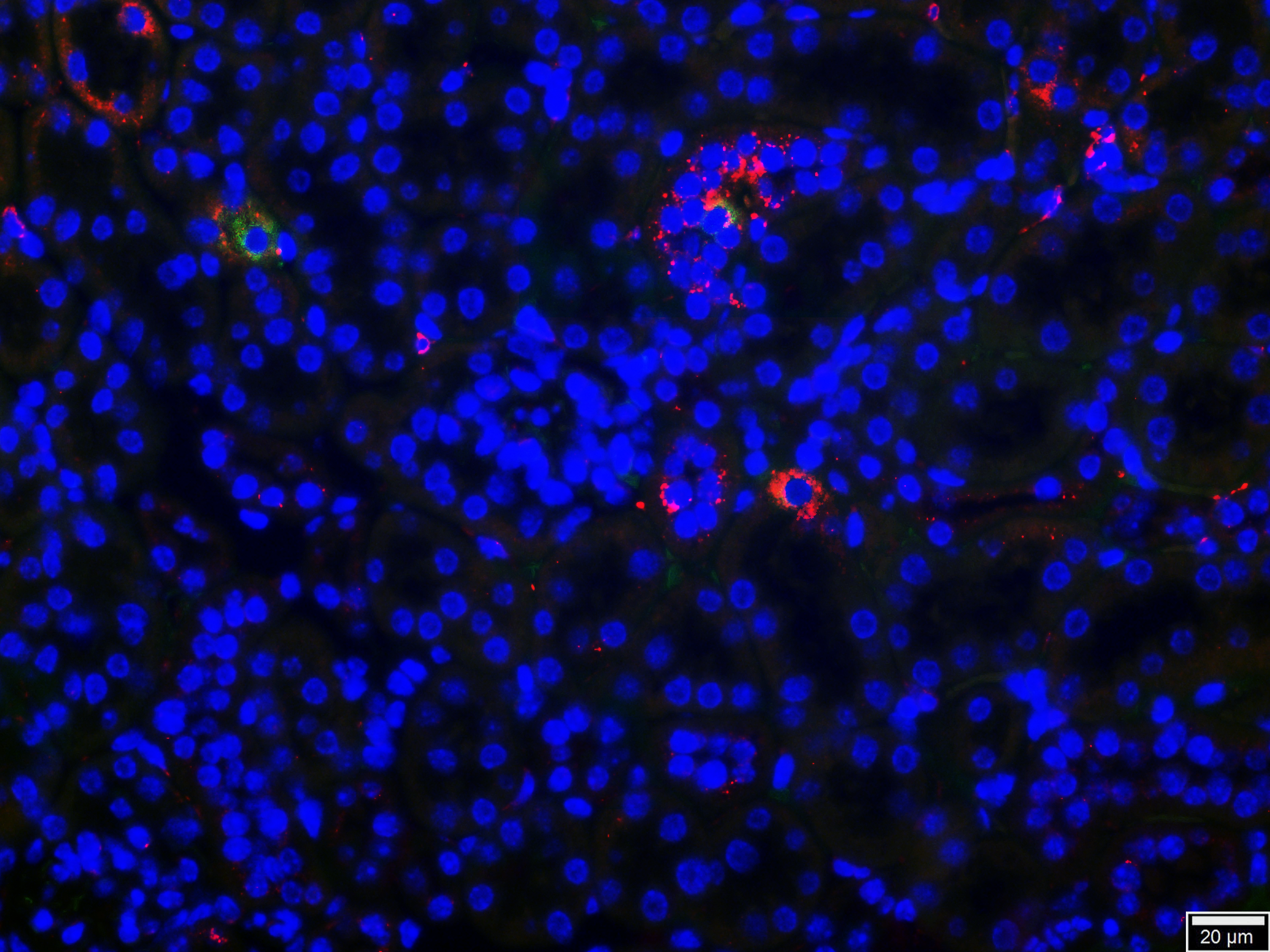

Supplement: Supplementary file 4 [file DataSheet4.ZIP › original data FIG8(I)/HLA-DR-1/1-8(400倍)-merge.tif]

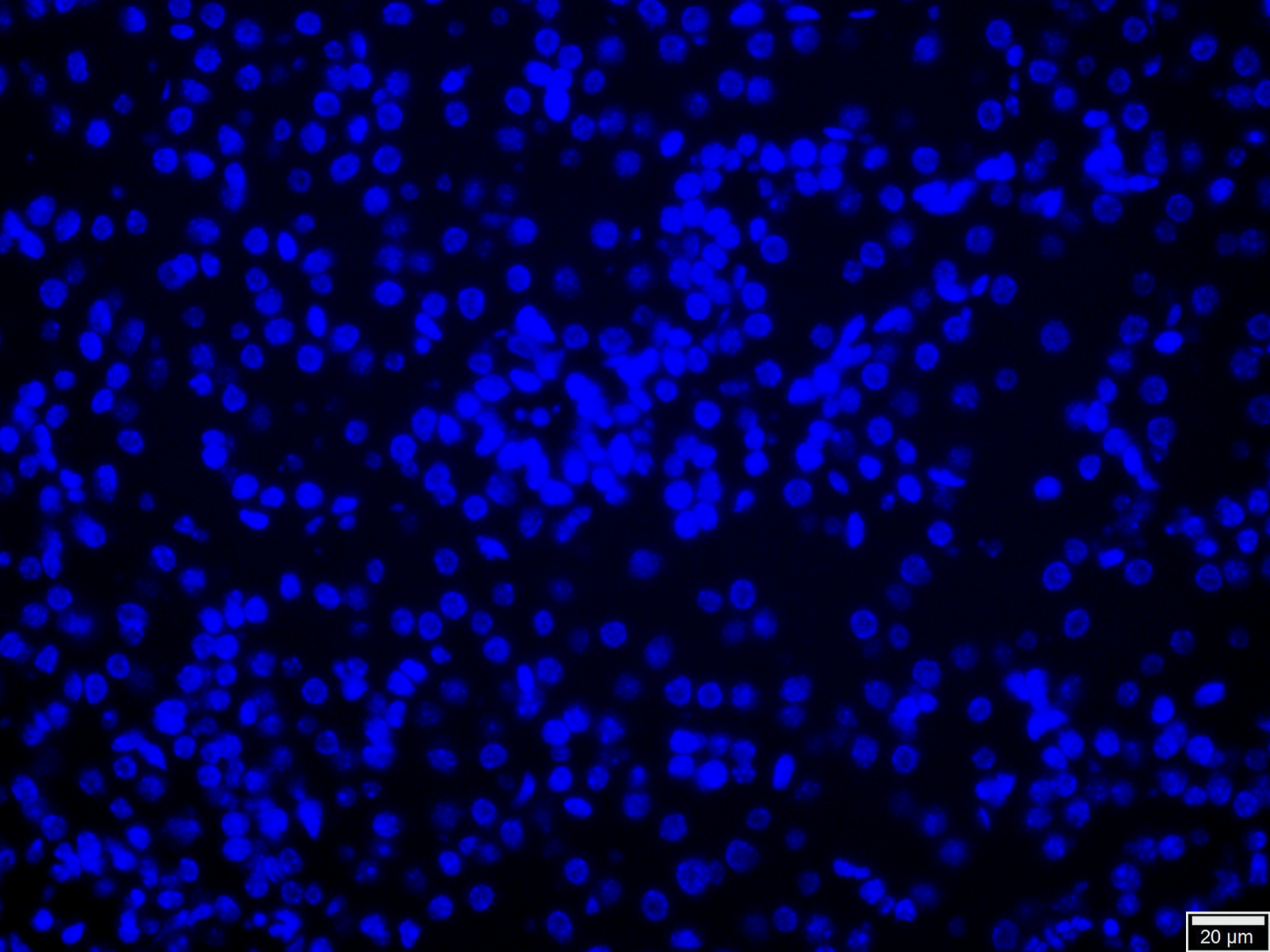

Supplement: Supplementary file 4 [file DataSheet4.ZIP › original data FIG8(I)/HLA-DR-1/1-8(400倍).tif]

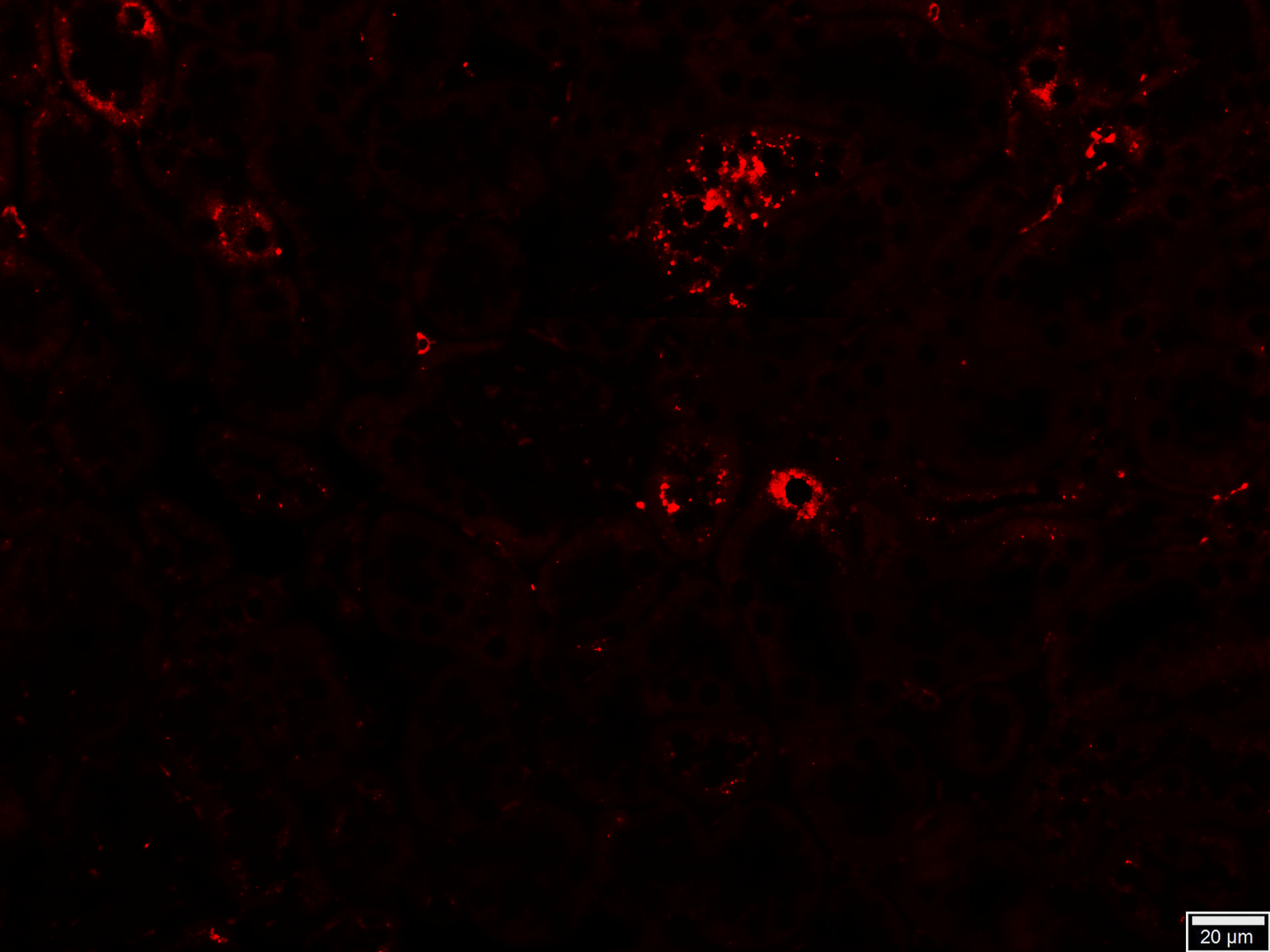

Supplement: Supplementary file 4 [file DataSheet4.ZIP › original data FIG8(I)/HLA-DR-1/1-9(400倍).tif]

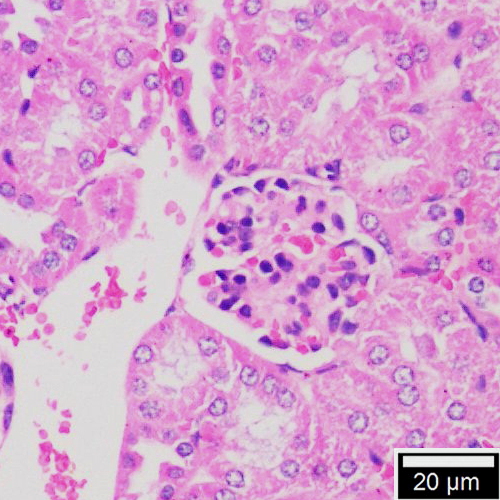

Supplement: Supplementary file 5 [file DataSheet1.ZIP › original data FIG1-2/Fig2A HE/1/1.tif]

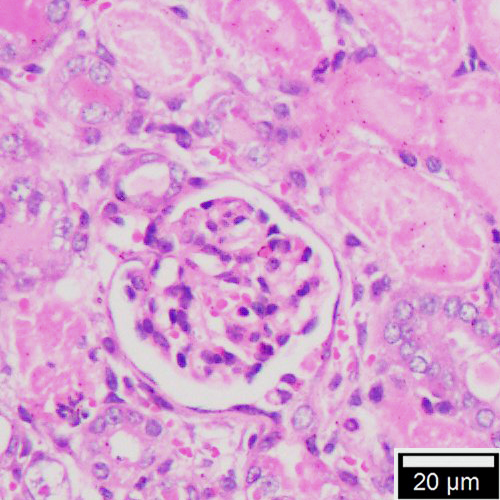

Supplement: Supplementary file 5 [file DataSheet1.ZIP › original data FIG1-2/Fig2A HE/1/2.tif]

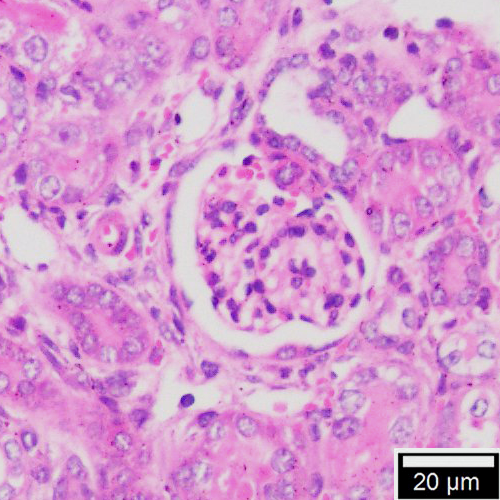

Supplement: Supplementary file 5 [file DataSheet1.ZIP › original data FIG1-2/Fig2A HE/1/3.tif]

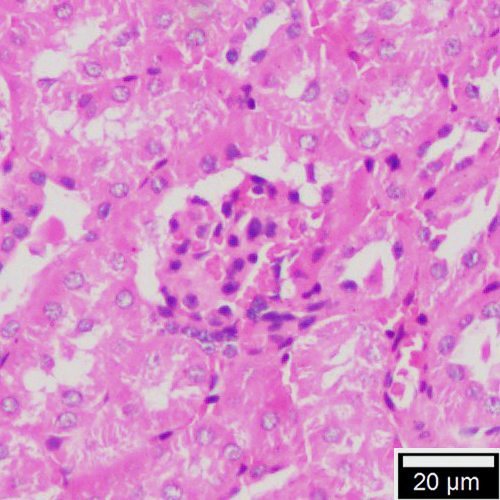

Supplement: Supplementary file 5 [file DataSheet1.ZIP › original data FIG1-2/Fig2A HE/2/1.tif]

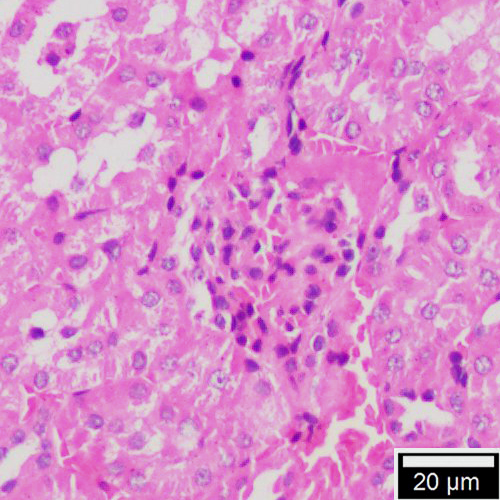

Supplement: Supplementary file 5 [file DataSheet1.ZIP › original data FIG1-2/Fig2A HE/2/2.tif]

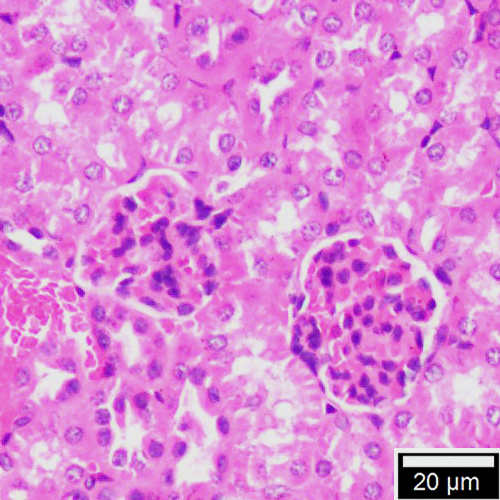

Supplement: Supplementary file 5 [file DataSheet1.ZIP › original data FIG1-2/Fig2A HE/2/3.tif]

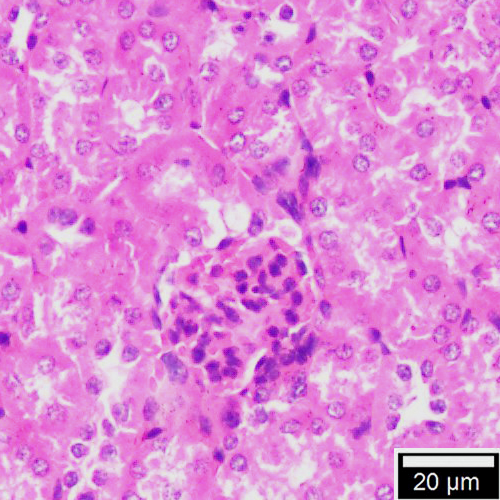

Supplement: Supplementary file 5 [file DataSheet1.ZIP › original data FIG1-2/Fig2A HE/3/1.tif]

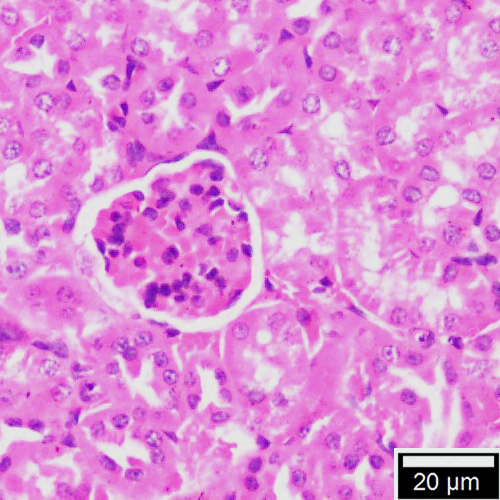

Supplement: Supplementary file 5 [file DataSheet1.ZIP › original data FIG1-2/Fig2A HE/3/2.tif]

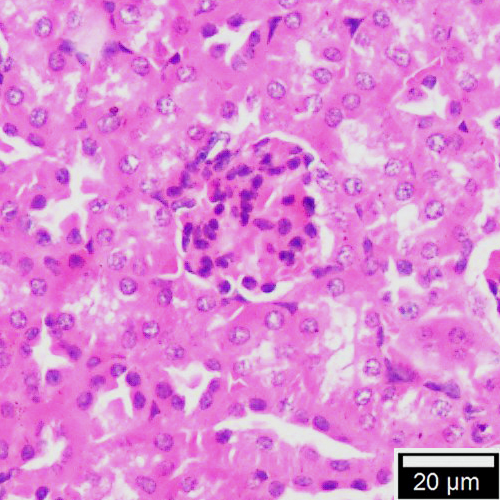

Supplement: Supplementary file 5 [file DataSheet1.ZIP › original data FIG1-2/Fig2A HE/3/3.tif]

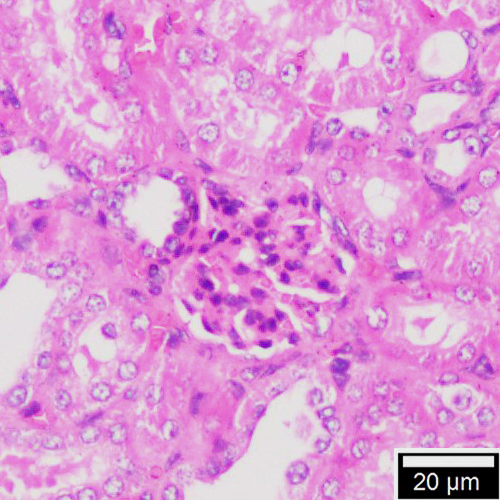

Supplement: Supplementary file 5 [file DataSheet1.ZIP › original data FIG1-2/Fig2A HE/4/1.tif]

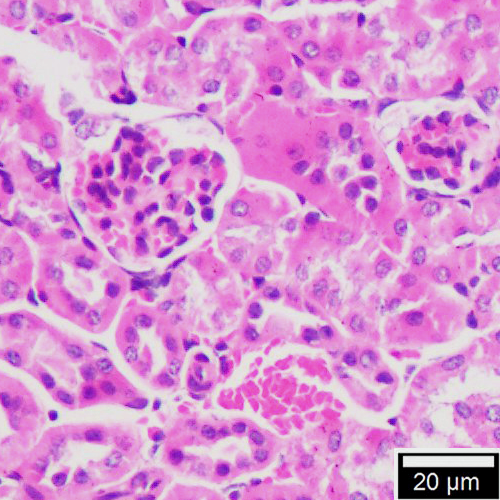

Supplement: Supplementary file 5 [file DataSheet1.ZIP › original data FIG1-2/Fig2A HE/4/2.tif]

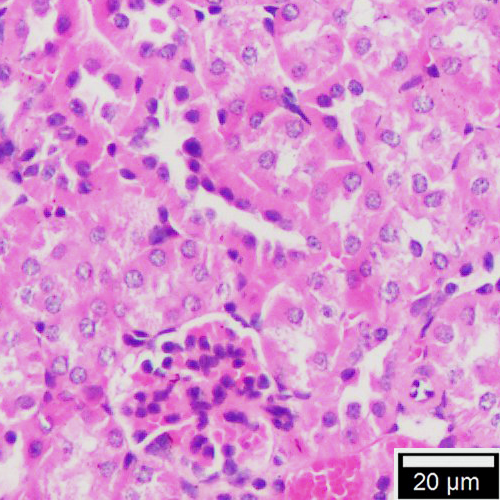

Supplement: Supplementary file 5 [file DataSheet1.ZIP › original data FIG1-2/Fig2A HE/4/3.tif]

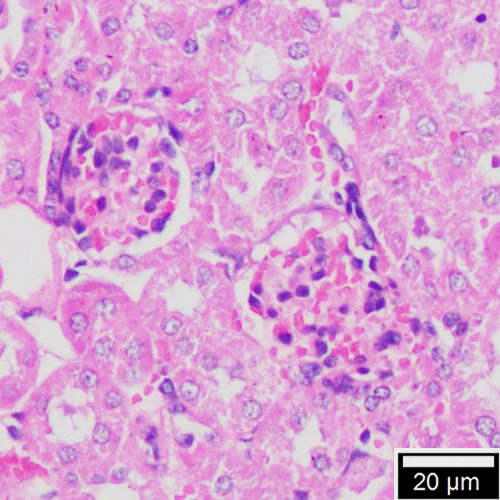

Supplement: Supplementary file 5 [file DataSheet1.ZIP › original data FIG1-2/Fig2A HE/5/1.tif]

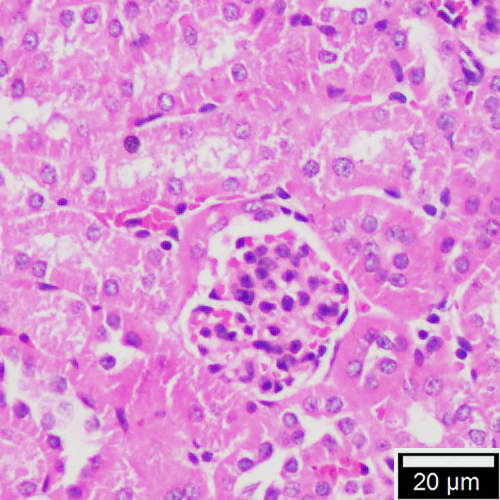

Supplement: Supplementary file 5 [file DataSheet1.ZIP › original data FIG1-2/Fig2A HE/5/2.tif]

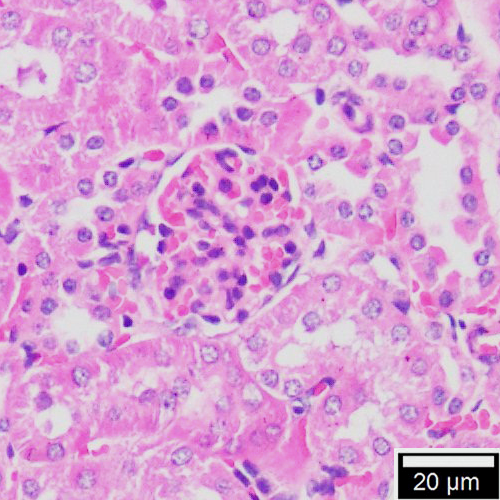

Supplement: Supplementary file 5 [file DataSheet1.ZIP › original data FIG1-2/Fig2A HE/5/3.tif]

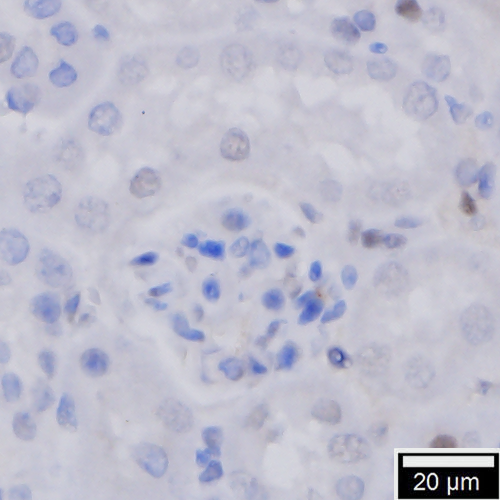

Supplement: Supplementary file 5 [file DataSheet1.ZIP › original data FIG1-2/Fig2B tunel/1/1.tif]

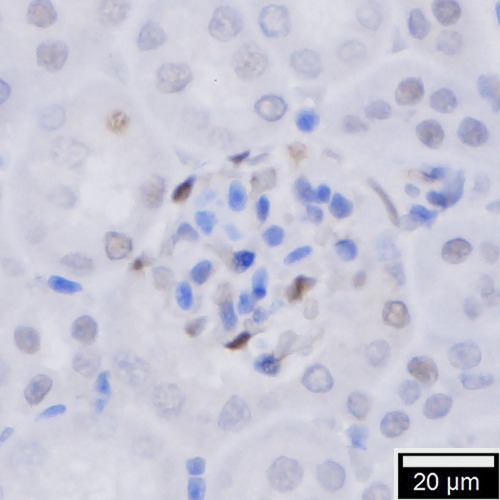

Supplement: Supplementary file 5 [file DataSheet1.ZIP › original data FIG1-2/Fig2B tunel/1/2.tif]

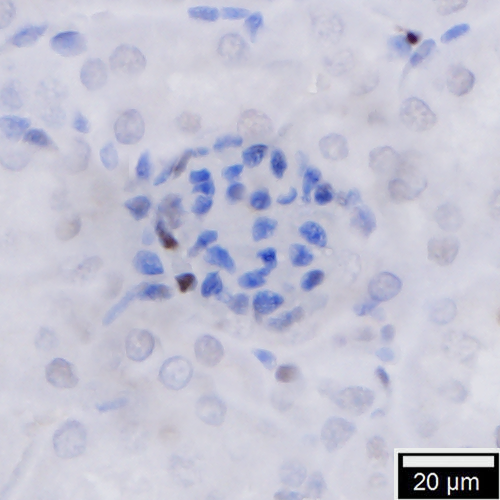

Supplement: Supplementary file 5 [file DataSheet1.ZIP › original data FIG1-2/Fig2B tunel/1/3.tif]

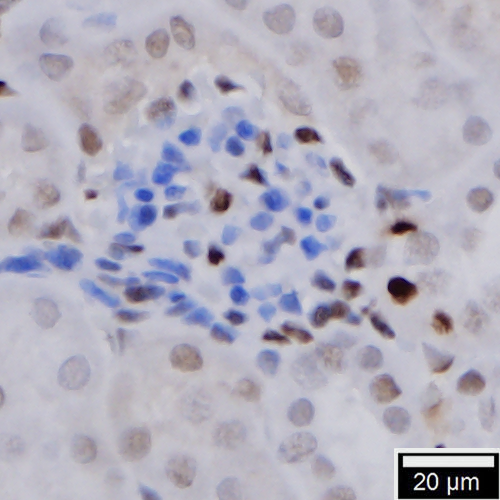

Supplement: Supplementary file 5 [file DataSheet1.ZIP › original data FIG1-2/Fig2B tunel/2/1.tif]

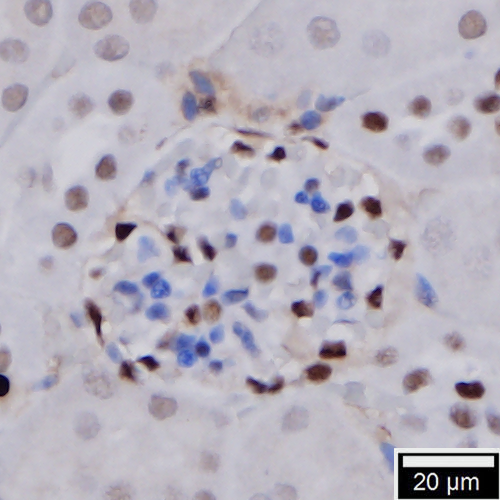

Supplement: Supplementary file 5 [file DataSheet1.ZIP › original data FIG1-2/Fig2B tunel/2/2.tif]

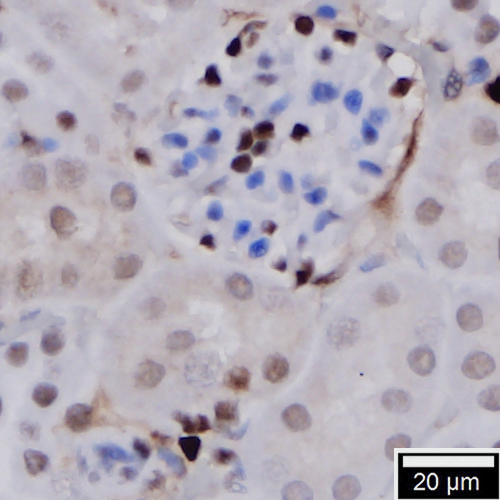

Supplement: Supplementary file 5 [file DataSheet1.ZIP › original data FIG1-2/Fig2B tunel/2/3.tif]

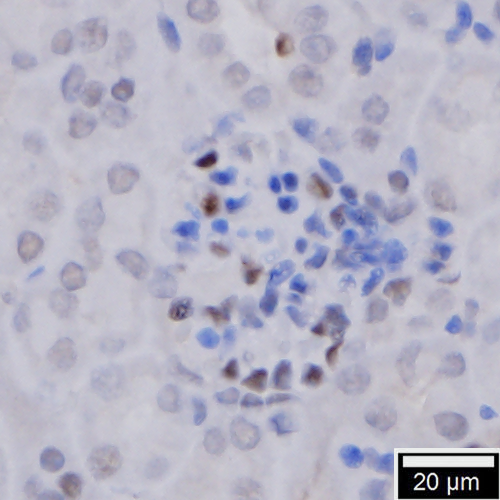

Supplement: Supplementary file 5 [file DataSheet1.ZIP › original data FIG1-2/Fig2B tunel/3/1.tif]

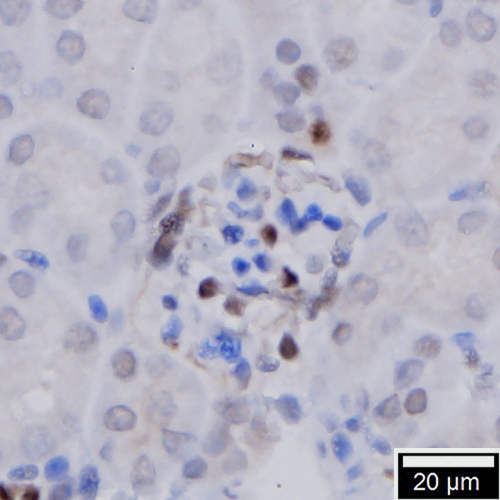

Supplement: Supplementary file 5 [file DataSheet1.ZIP › original data FIG1-2/Fig2B tunel/3/2.tif]

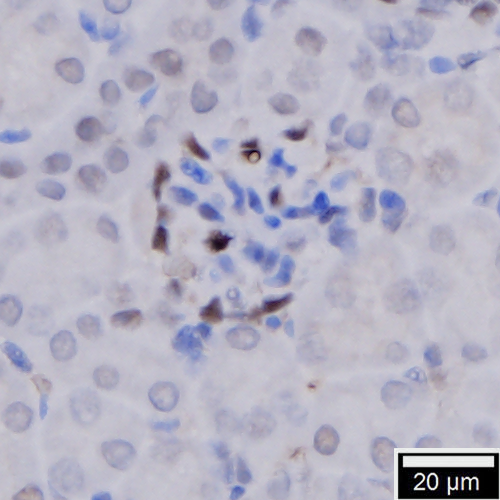

Supplement: Supplementary file 5 [file DataSheet1.ZIP › original data FIG1-2/Fig2B tunel/3/3.tif]

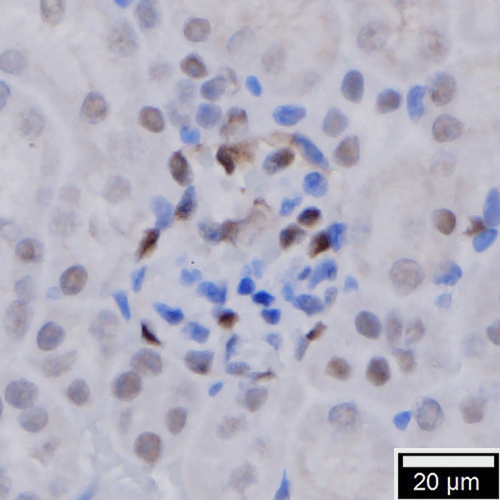

Supplement: Supplementary file 5 [file DataSheet1.ZIP › original data FIG1-2/Fig2B tunel/4/1.tif]

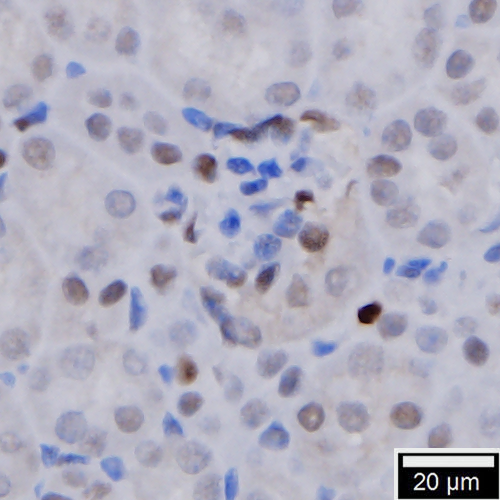

Supplement: Supplementary file 5 [file DataSheet1.ZIP › original data FIG1-2/Fig2B tunel/4/2.tif]

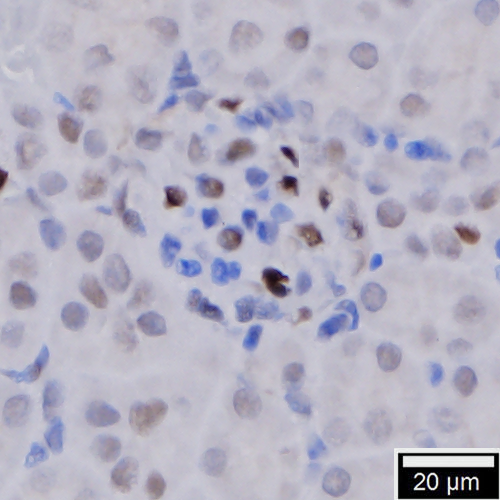

Supplement: Supplementary file 5 [file DataSheet1.ZIP › original data FIG1-2/Fig2B tunel/4/3.tif]

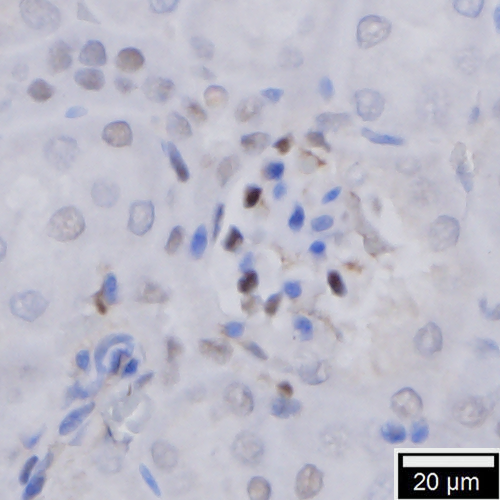

Supplement: Supplementary file 5 [file DataSheet1.ZIP › original data FIG1-2/Fig2B tunel/5/1.tif]

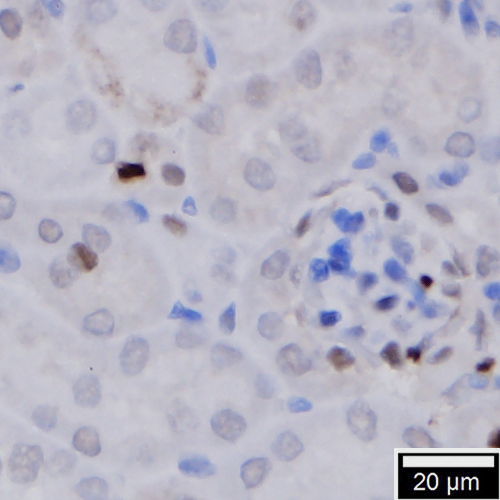

Supplement: Supplementary file 5 [file DataSheet1.ZIP › original data FIG1-2/Fig2B tunel/5/2.tif]

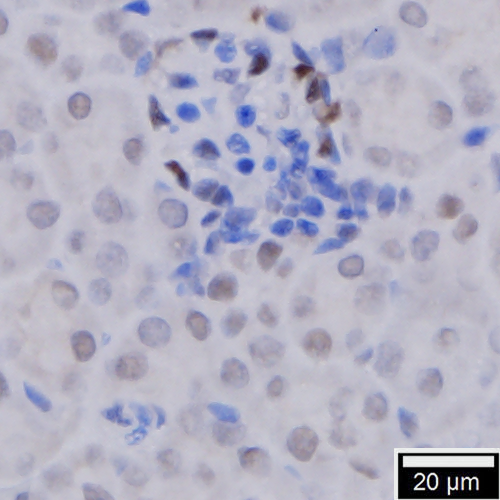

Supplement: Supplementary file 5 [file DataSheet1.ZIP › original data FIG1-2/Fig2B tunel/5/3.tif]

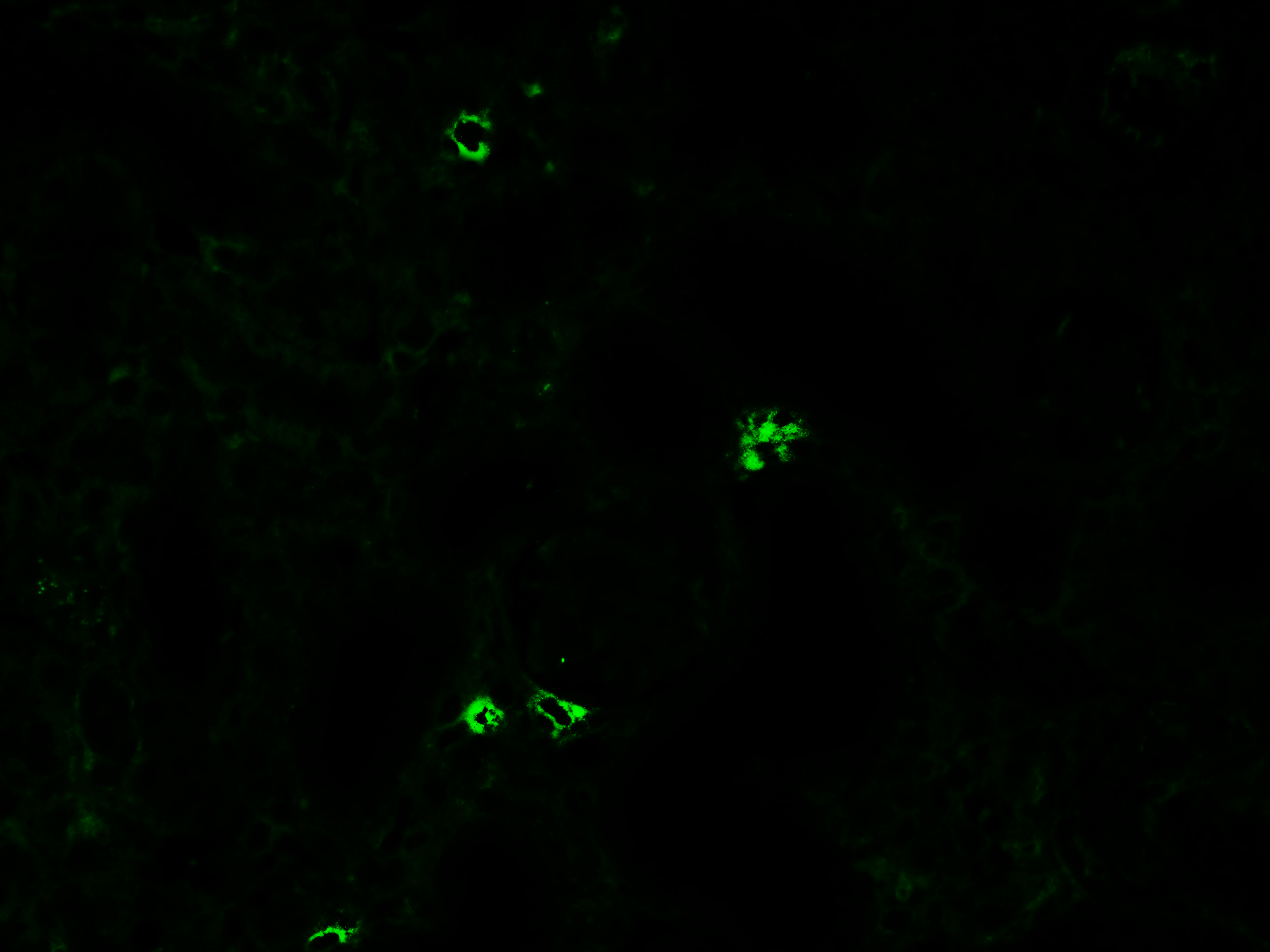

Supplement: Supplementary file 6 [file DataSheet6.ZIP › original data FIG8(III)/HLA-DR-3/3-1(400倍).tif]

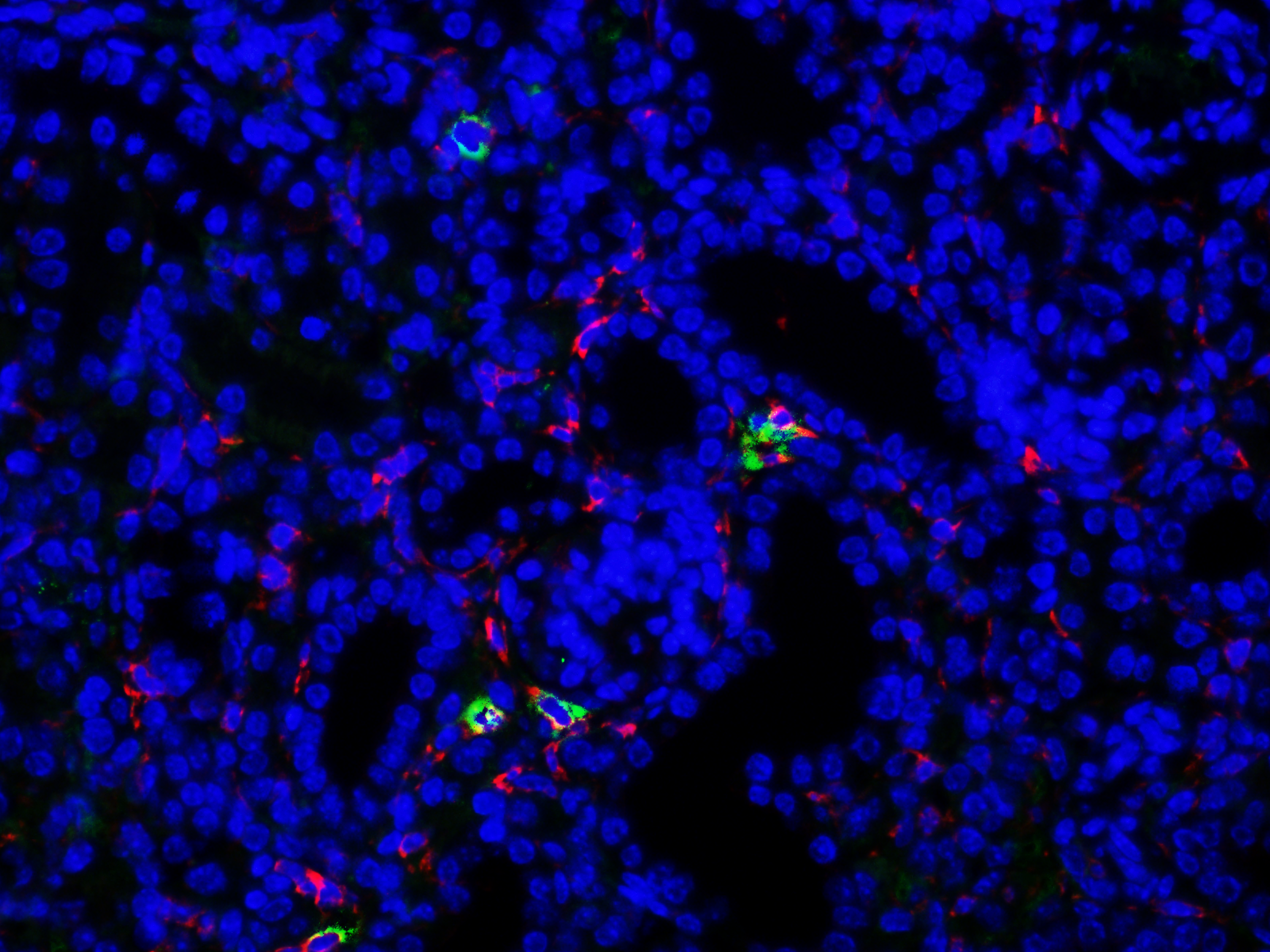

Supplement: Supplementary file 6 [file DataSheet6.ZIP › original data FIG8(III)/HLA-DR-3/3-2(400倍)-merge.tif]

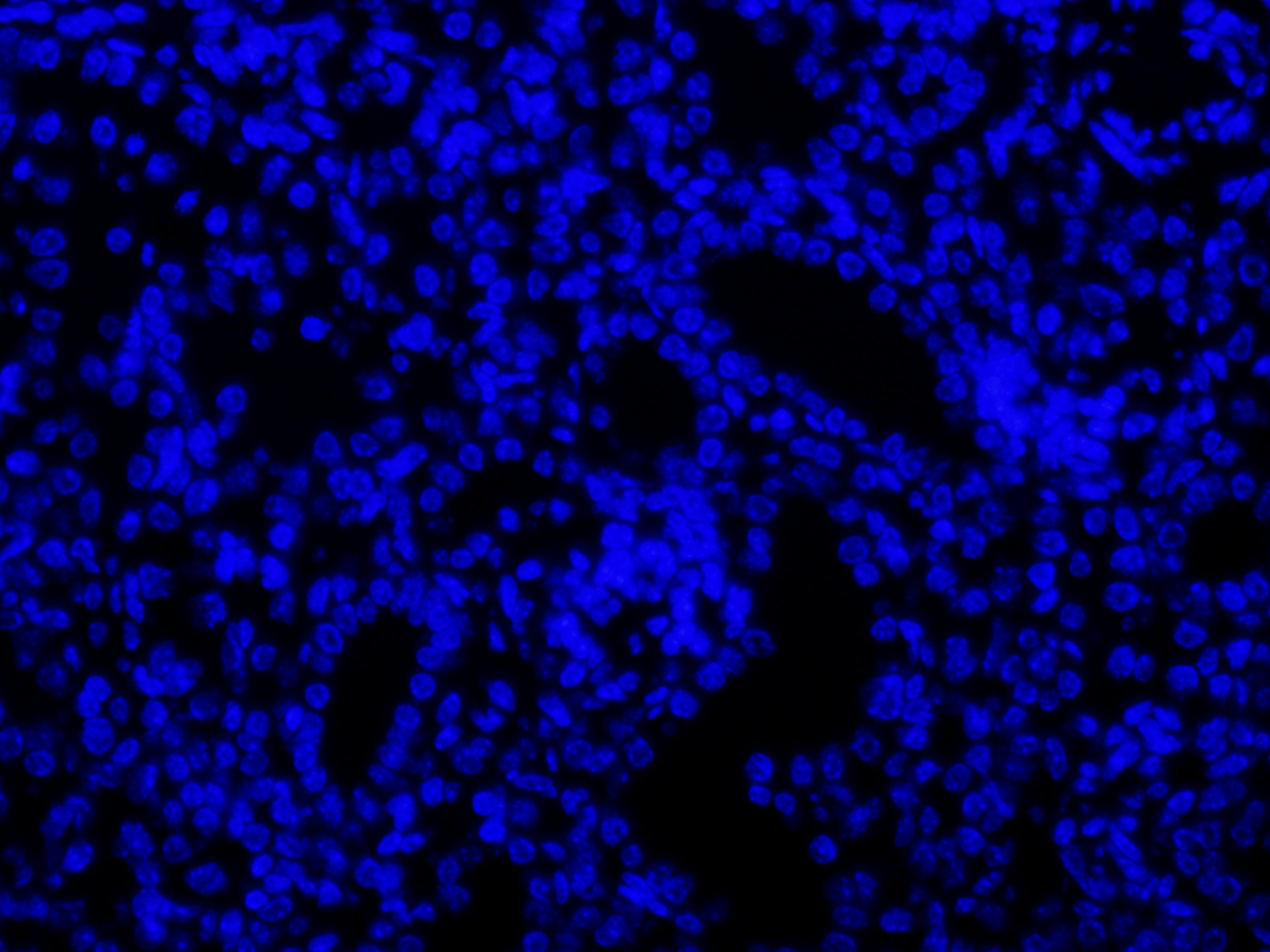

Supplement: Supplementary file 6 [file DataSheet6.ZIP › original data FIG8(III)/HLA-DR-3/3-2(400倍).tif]

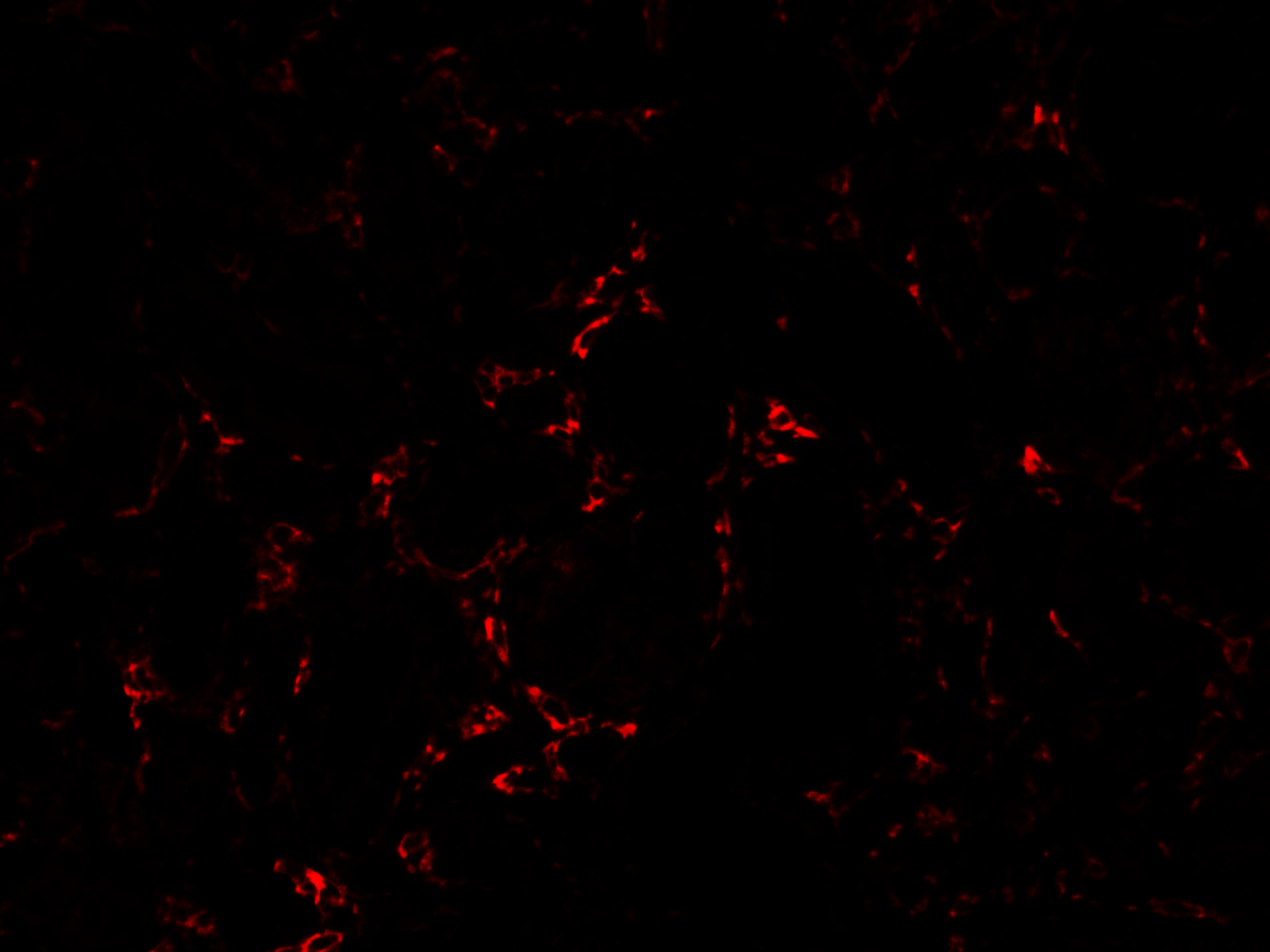

Supplement: Supplementary file 6 [file DataSheet6.ZIP › original data FIG8(III)/HLA-DR-3/3-3(400倍).tif]

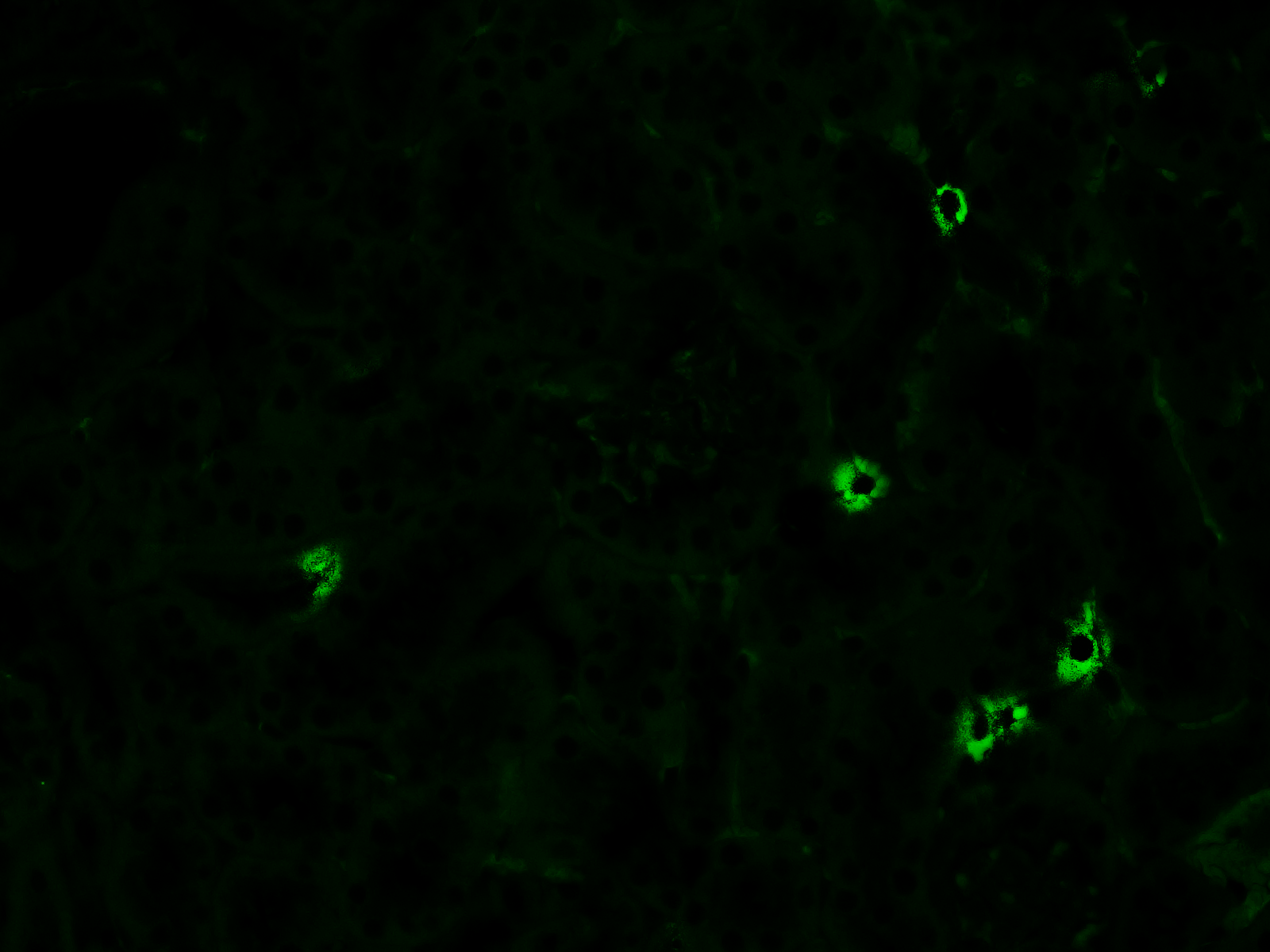

Supplement: Supplementary file 6 [file DataSheet6.ZIP › original data FIG8(III)/HLA-DR-3/3-4(400倍).tif]

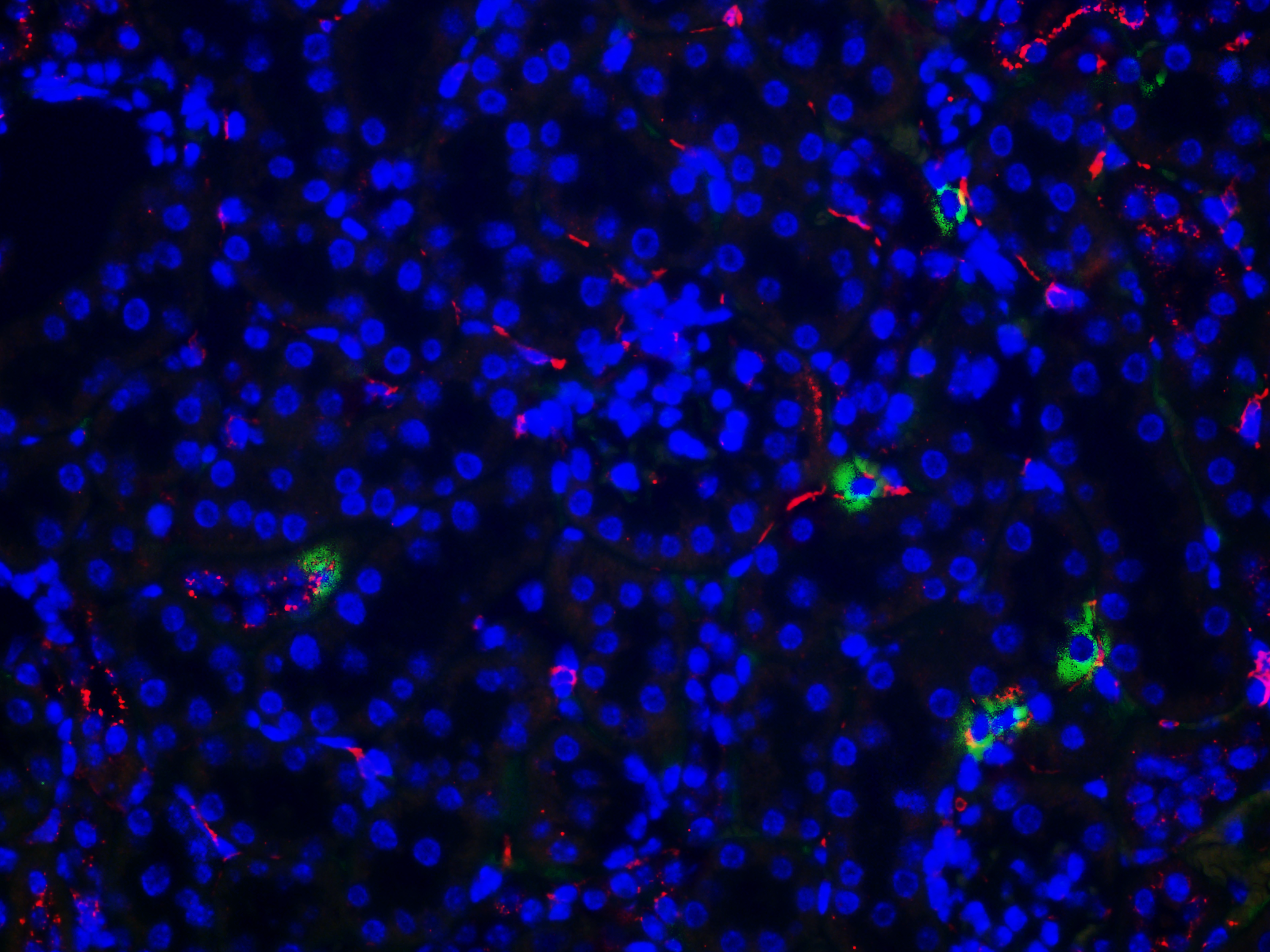

Supplement: Supplementary file 6 [file DataSheet6.ZIP › original data FIG8(III)/HLA-DR-3/3-5(400倍)-merge.tif]

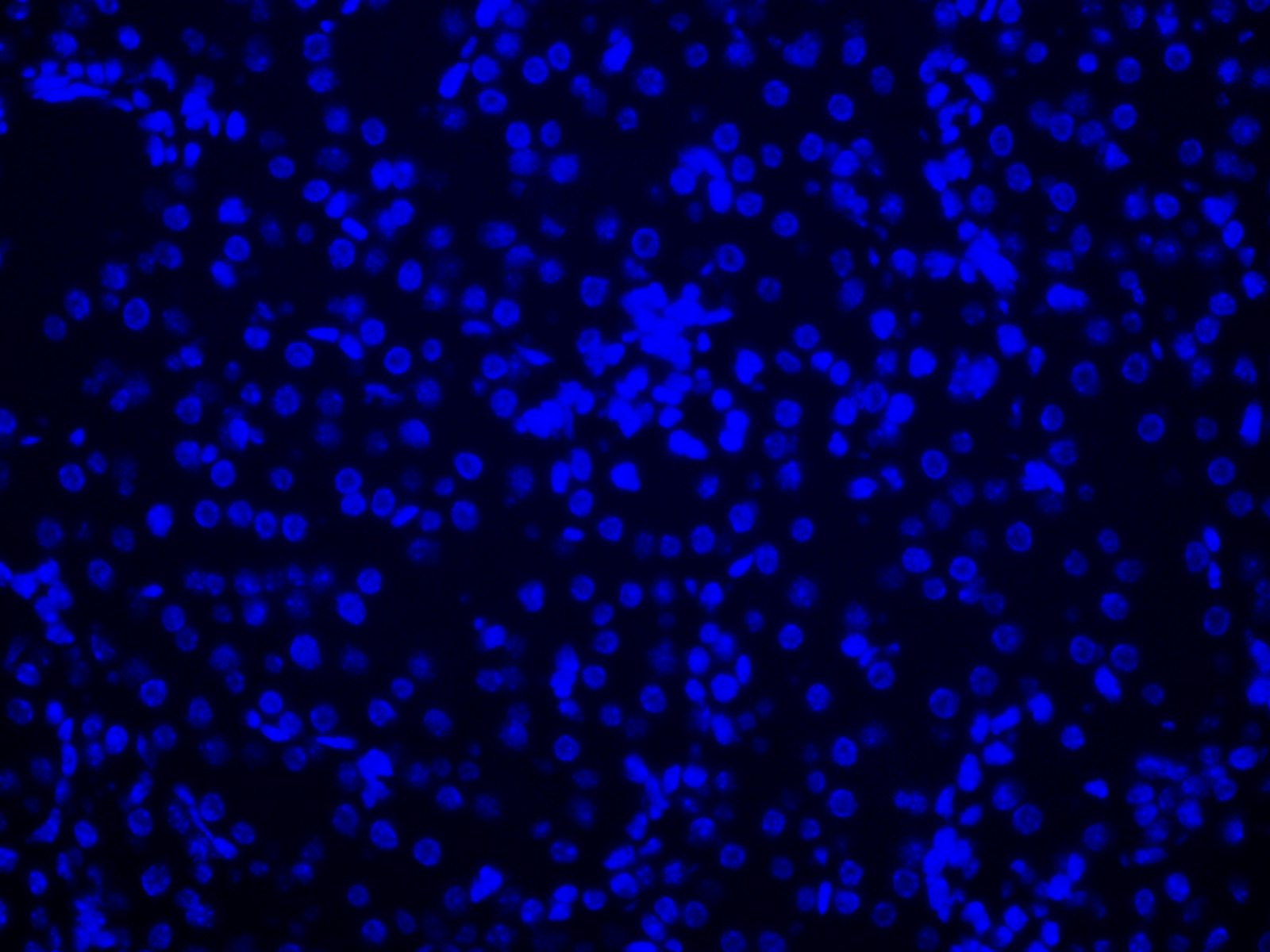

Supplement: Supplementary file 6 [file DataSheet6.ZIP › original data FIG8(III)/HLA-DR-3/3-5(400倍).tif]

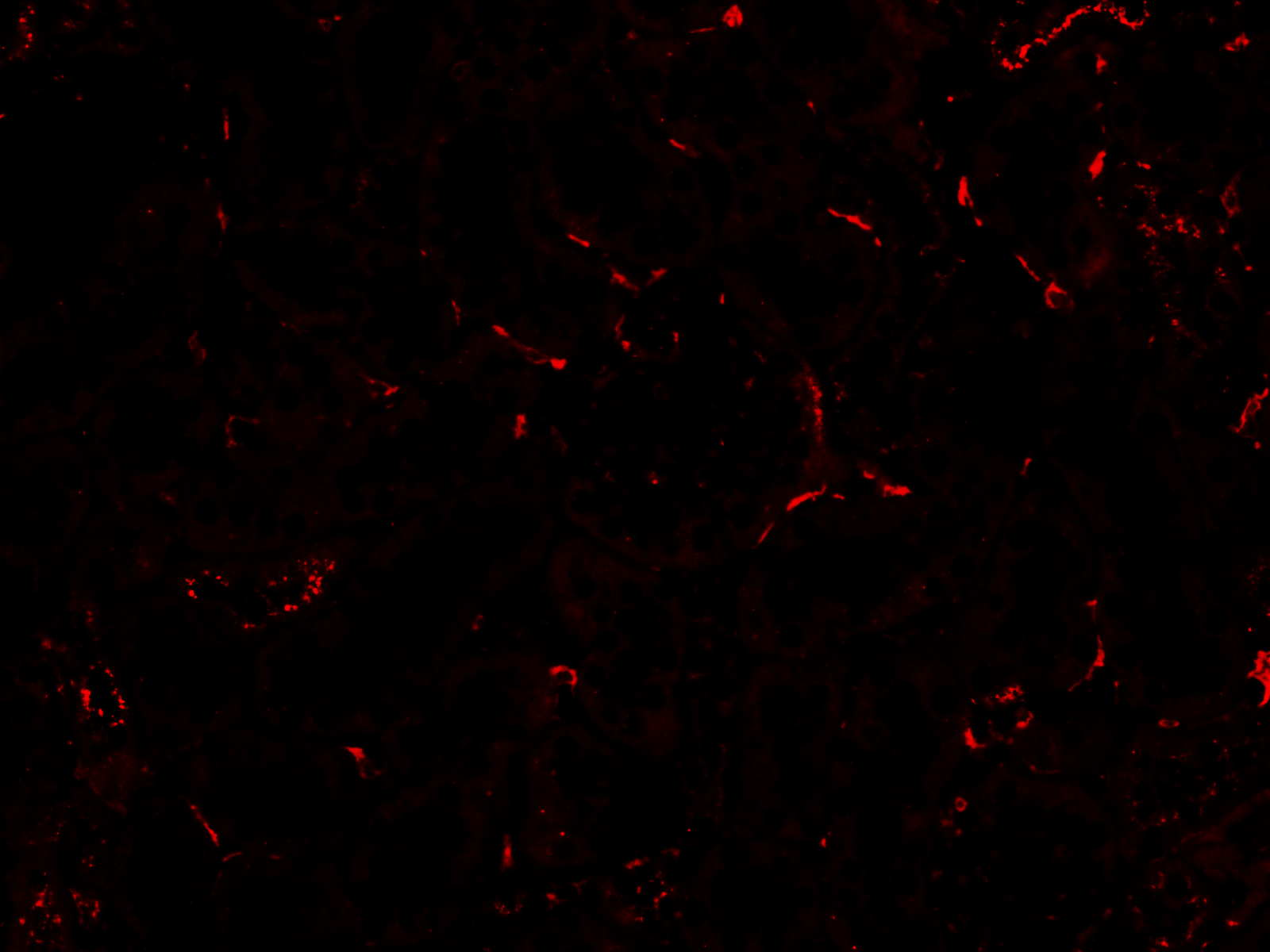

Supplement: Supplementary file 6 [file DataSheet6.ZIP › original data FIG8(III)/HLA-DR-3/3-6(400倍).tif]

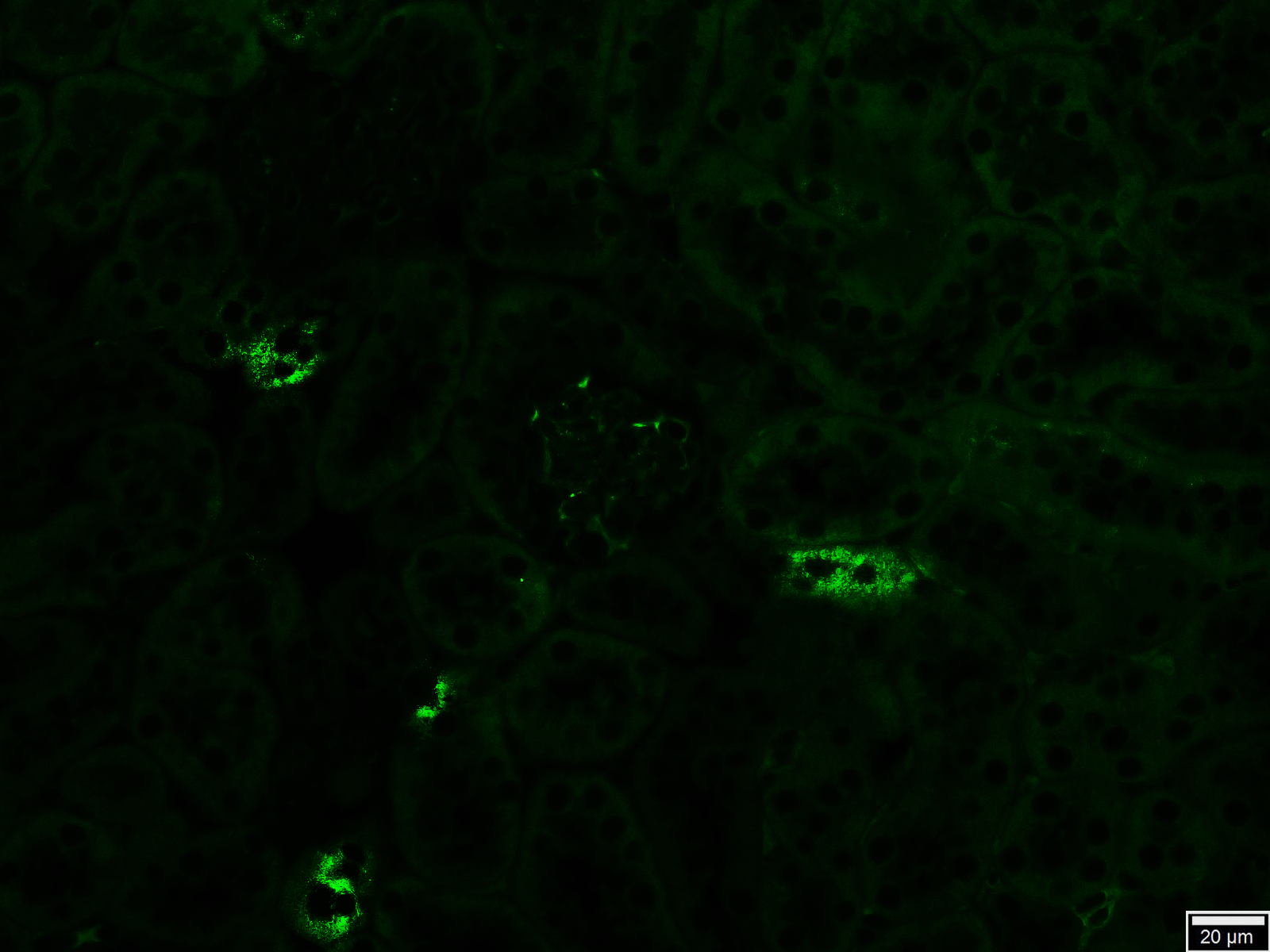

Supplement: Supplementary file 6 [file DataSheet6.ZIP › original data FIG8(III)/HLA-DR-3/3-7(400倍).tif]

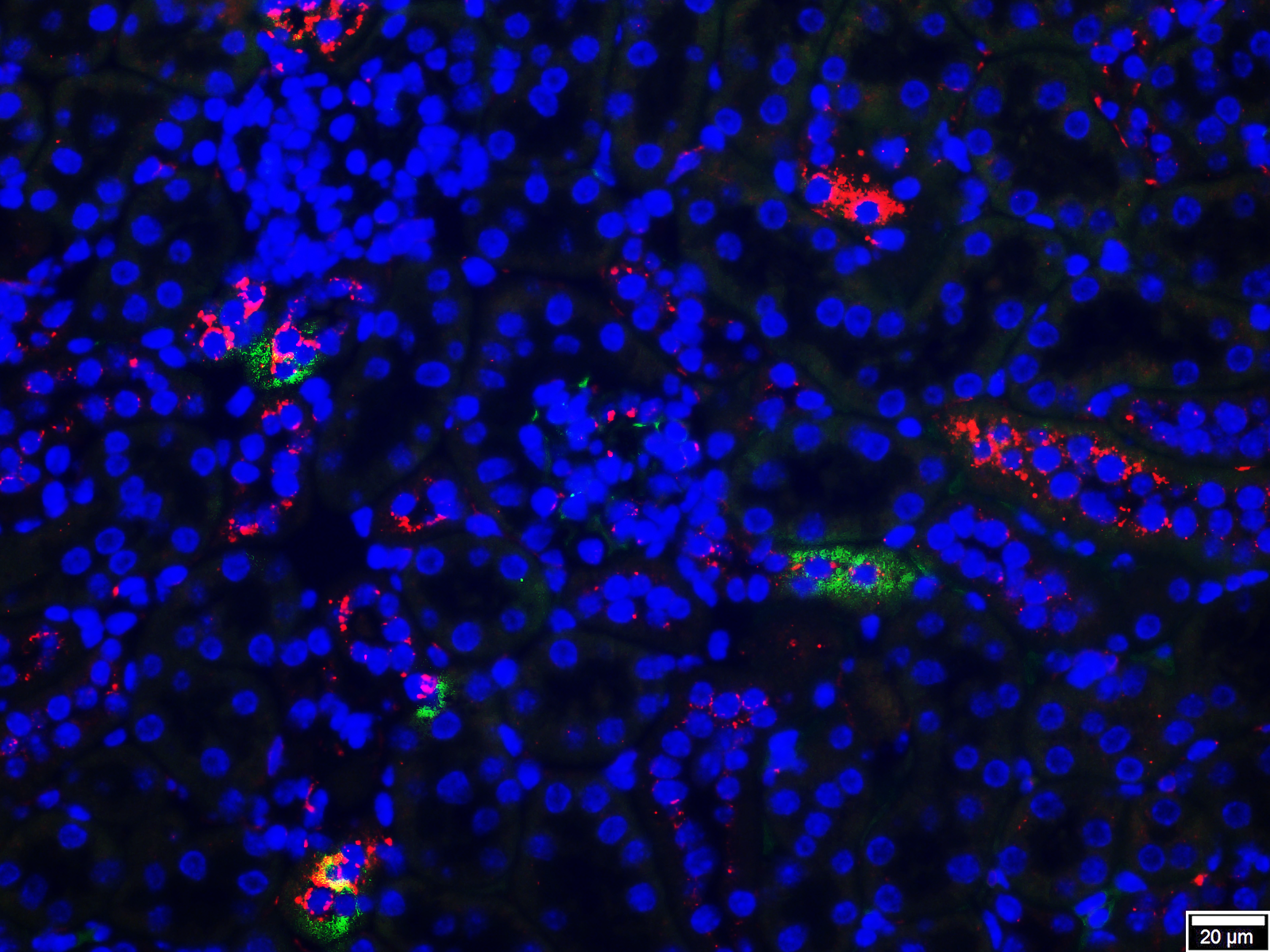

Supplement: Supplementary file 6 [file DataSheet6.ZIP › original data FIG8(III)/HLA-DR-3/3-8(400倍)-merg.tif]

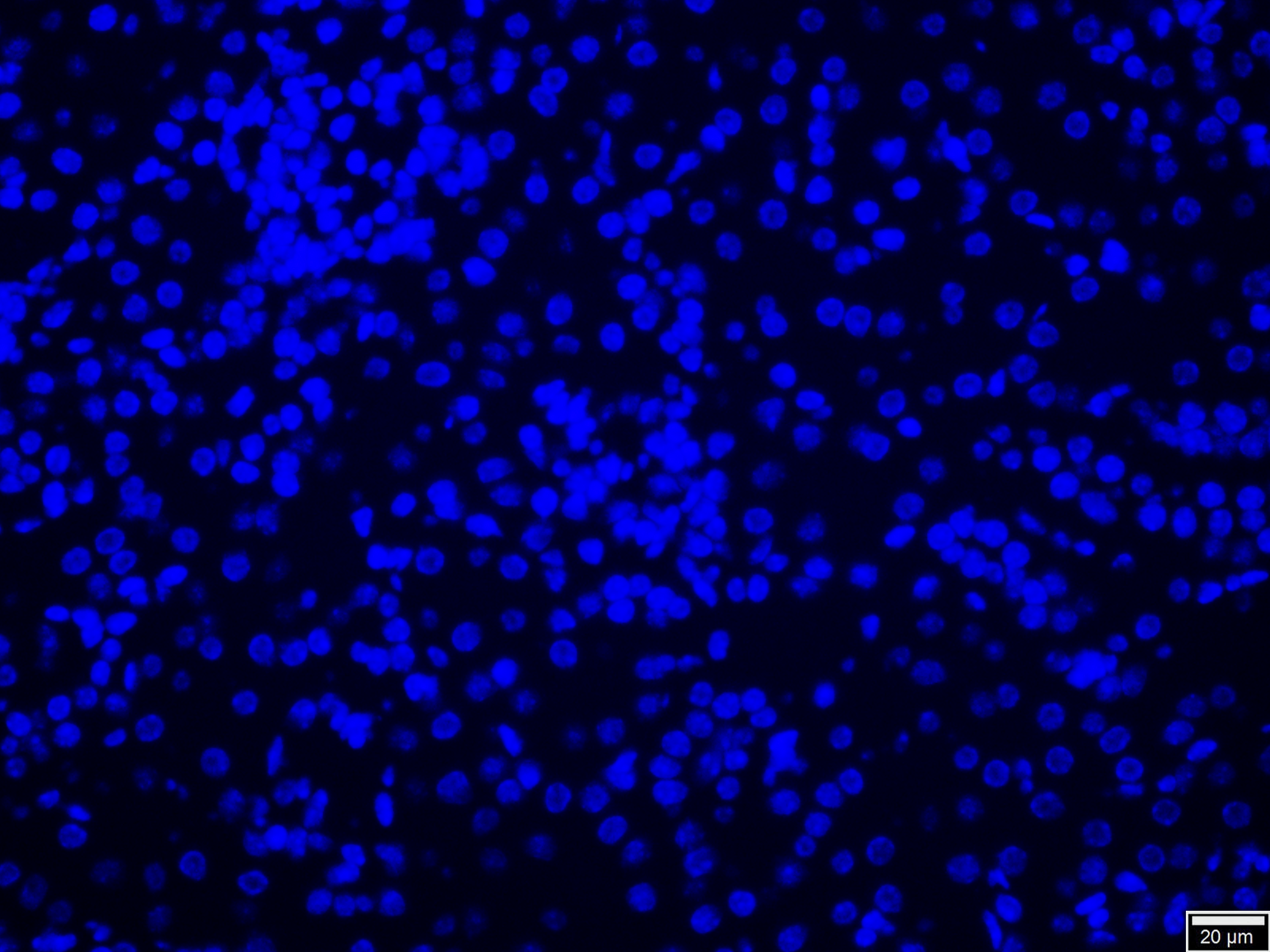

Supplement: Supplementary file 6 [file DataSheet6.ZIP › original data FIG8(III)/HLA-DR-3/3-8(400倍).tif]

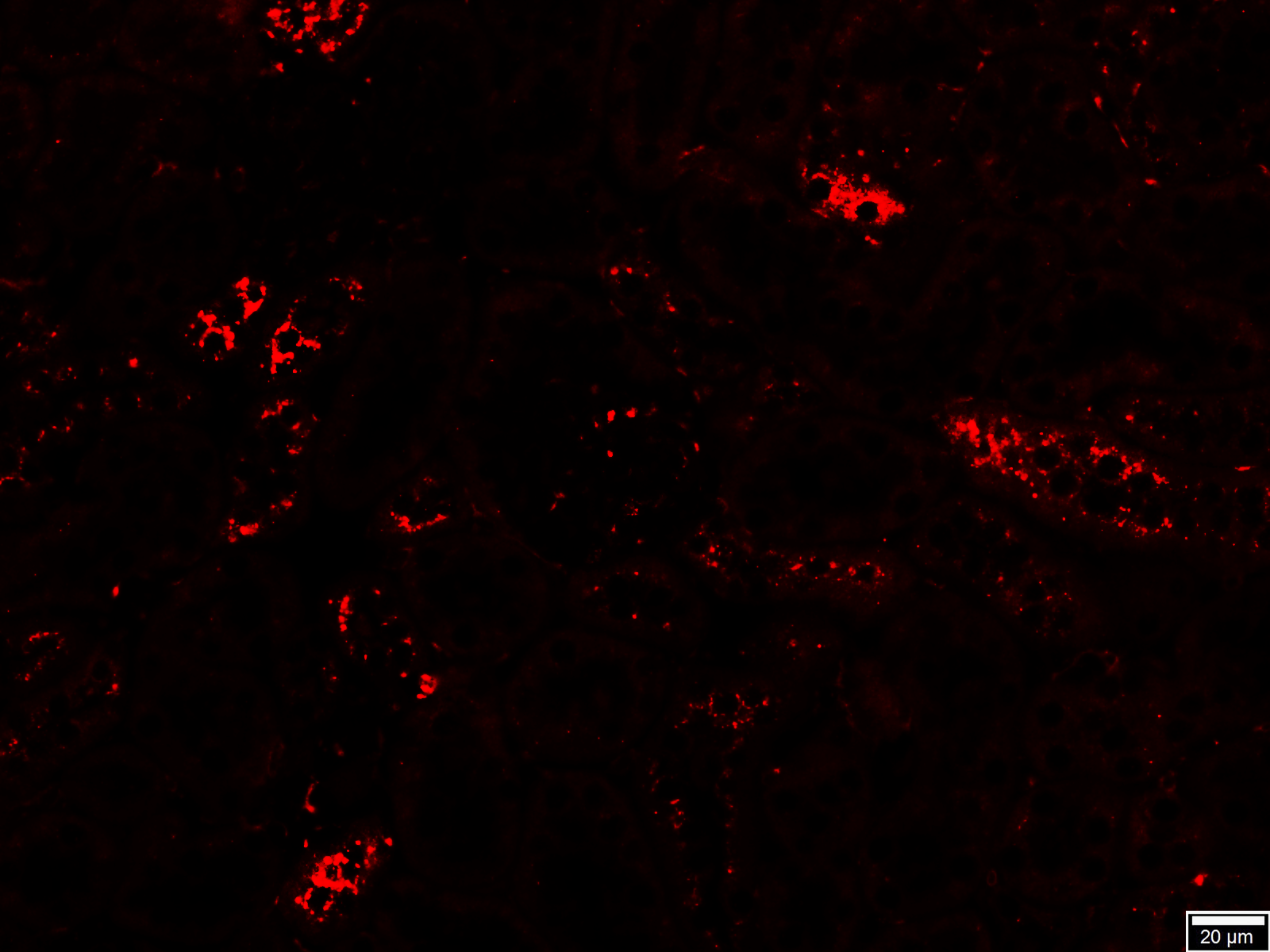

Supplement: Supplementary file 6 [file DataSheet6.ZIP › original data FIG8(III)/HLA-DR-3/3-9(400倍).tif]

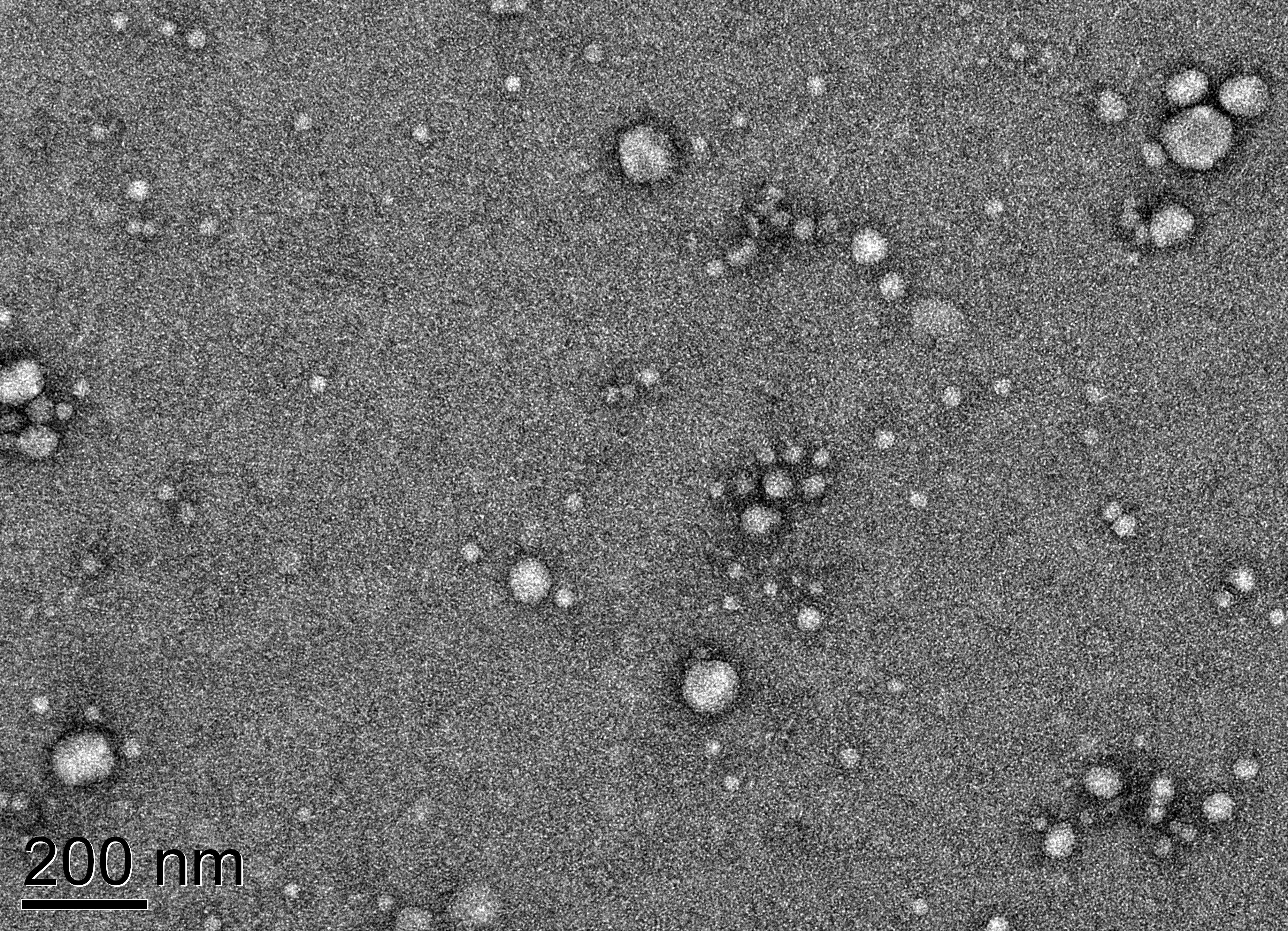

Supplement: Supplementary file 8 [file DataSheet2.ZIP › original data FIG3/Fig3A Exosome electron microscopy/1/B101.jpg]
