# Supplementary material for: Yi-Shen-Hua-Shi granules inhibit diabetic nephropathy by ameliorating podocyte injury induced by macrophage-derived exosomes
Source: Front Pharmacol. 2022 Nov 25;13:962606. doi: 10.3389/fphar.2022.962606 (PMC9732029; doi:10.3389/fphar.2022.962606)
Supplement: Supplementary file 3 [file Table1.DOCX]

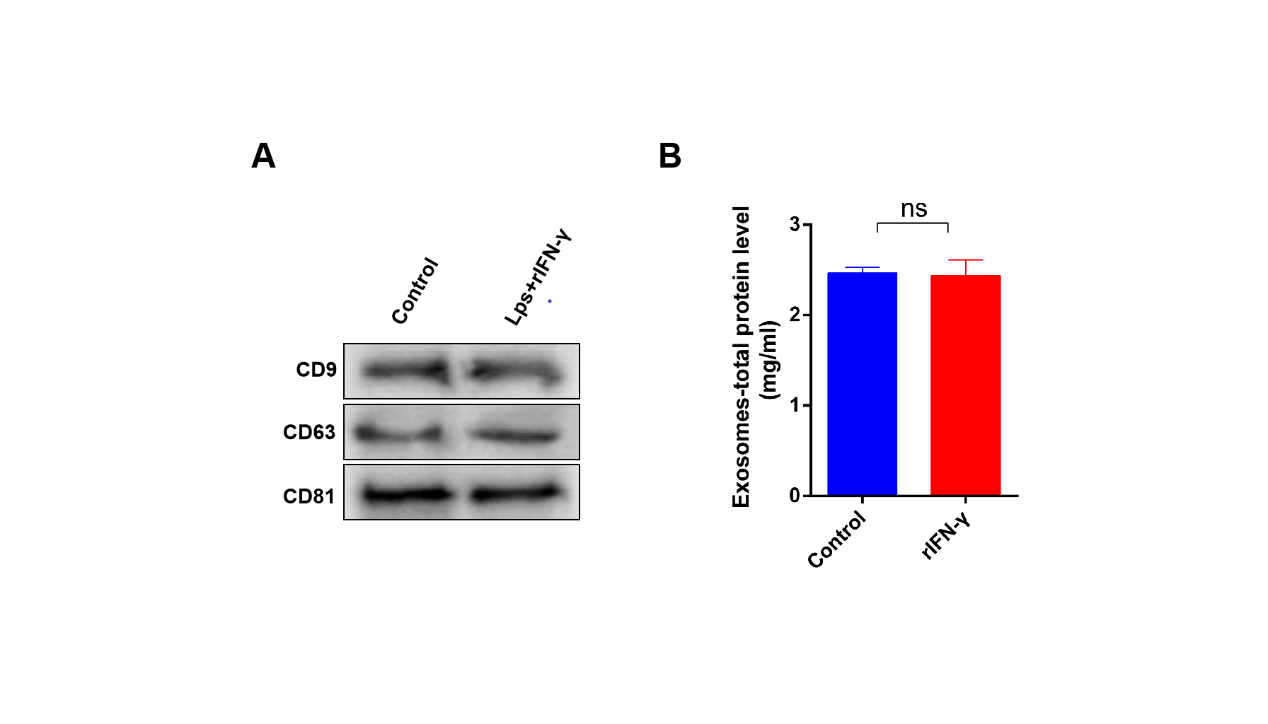
**Supplemental Figure 1. Exosomes identification and effect of macrophage M1 polarization on macrophage-derived exosome concentration.**

**A** easurement of protein level of CD9, CD63, and CD81 of exosome from macrophage treated with or without LPS (100ng/ml) and recombinant IFN-γ (20ng/ml) for 24h by western blotting. **B** Measurement of the protein Concentration of exosome from macrophage treated with or without LPS (100ng/ml) and recombinant IFN-γ (20ng/ml) for 24h by BCA kit. Data analysis adopts student’s t-test. SD indicates error bars. ns p>0.05.
