# Supplementary material for: Yi-Shen-Hua-Shi granules inhibit diabetic nephropathy by ameliorating podocyte injury induced by macrophage-derived exosomes
Source: Front Pharmacol. 2022 Nov 25;13:962606. doi: 10.3389/fphar.2022.962606 (PMC9732029; doi:10.3389/fphar.2022.962606)
Supplement: Supplementary file 7 [file Table2.DOCX]

| Gene | Primer (5’-3’) |
| --- | --- |
| miR-125a-5p | TGCGGCTCCCTGAGACCCTTTAAC |
| miR-148a-3p | TGCGGCTCAGTGCACTACAGAA |
| miR-21a-5p | TTGCGGCAGCTTATCAGACTGA |
| miR universal primer | CCAGTCTCAGGGTCCGAGGTATTC |
| U6 (Forward) | CTCGCTTCGGCAGCACA |
| U6 (Reverse) | AACGCTTCACGAATTTGCGT |

TABLE 1 | Primer sequences for real-time PCR analysis.
