# Supplementary figures and images for: Yi-Shen-Hua-Shi granules inhibit diabetic nephropathy by ameliorating podocyte injury induced by macrophage-derived exosomes (part 2 of 2)
Source: Front Pharmacol. 2022 Nov 25;13:962606. doi: 10.3389/fphar.2022.962606 (PMC9732029; doi:10.3389/fphar.2022.962606)

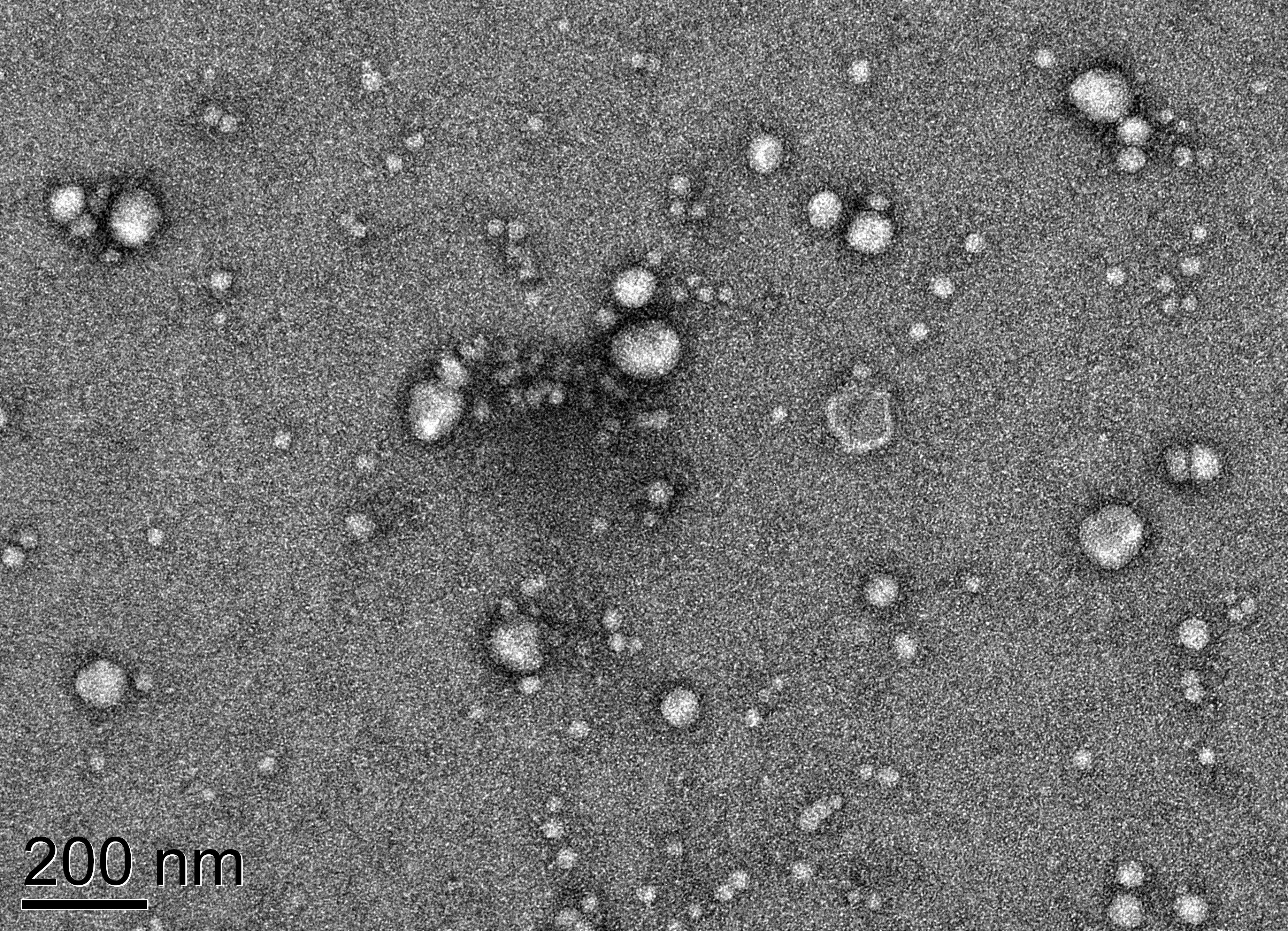

Supplement: Supplementary file 8 [file DataSheet2.ZIP › original data FIG3/Fig3A Exosome electron microscopy/1/B102.jpg]

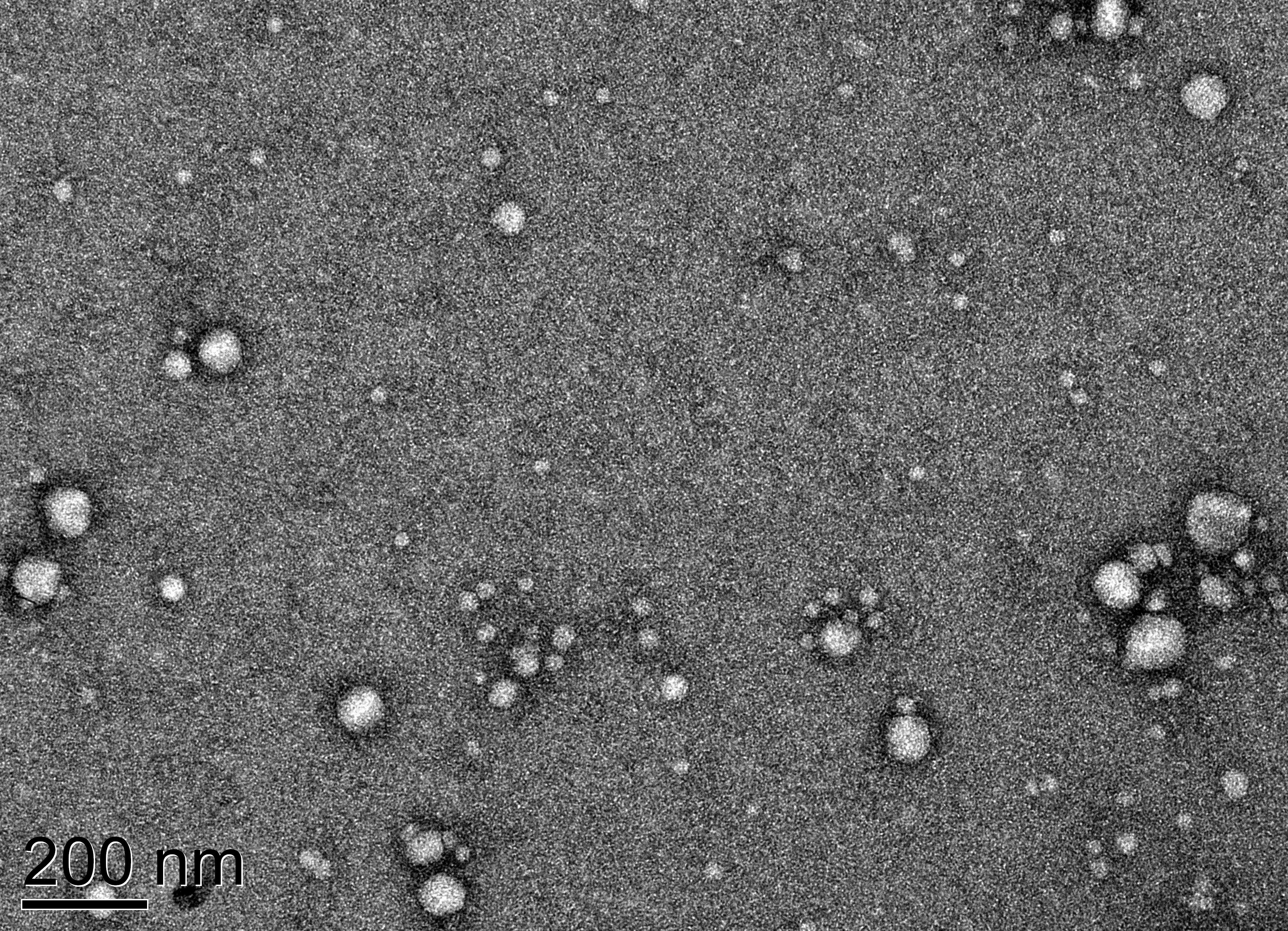

Supplement: Supplementary file 8 [file DataSheet2.ZIP › original data FIG3/Fig3A Exosome electron microscopy/1/B103.jpg]

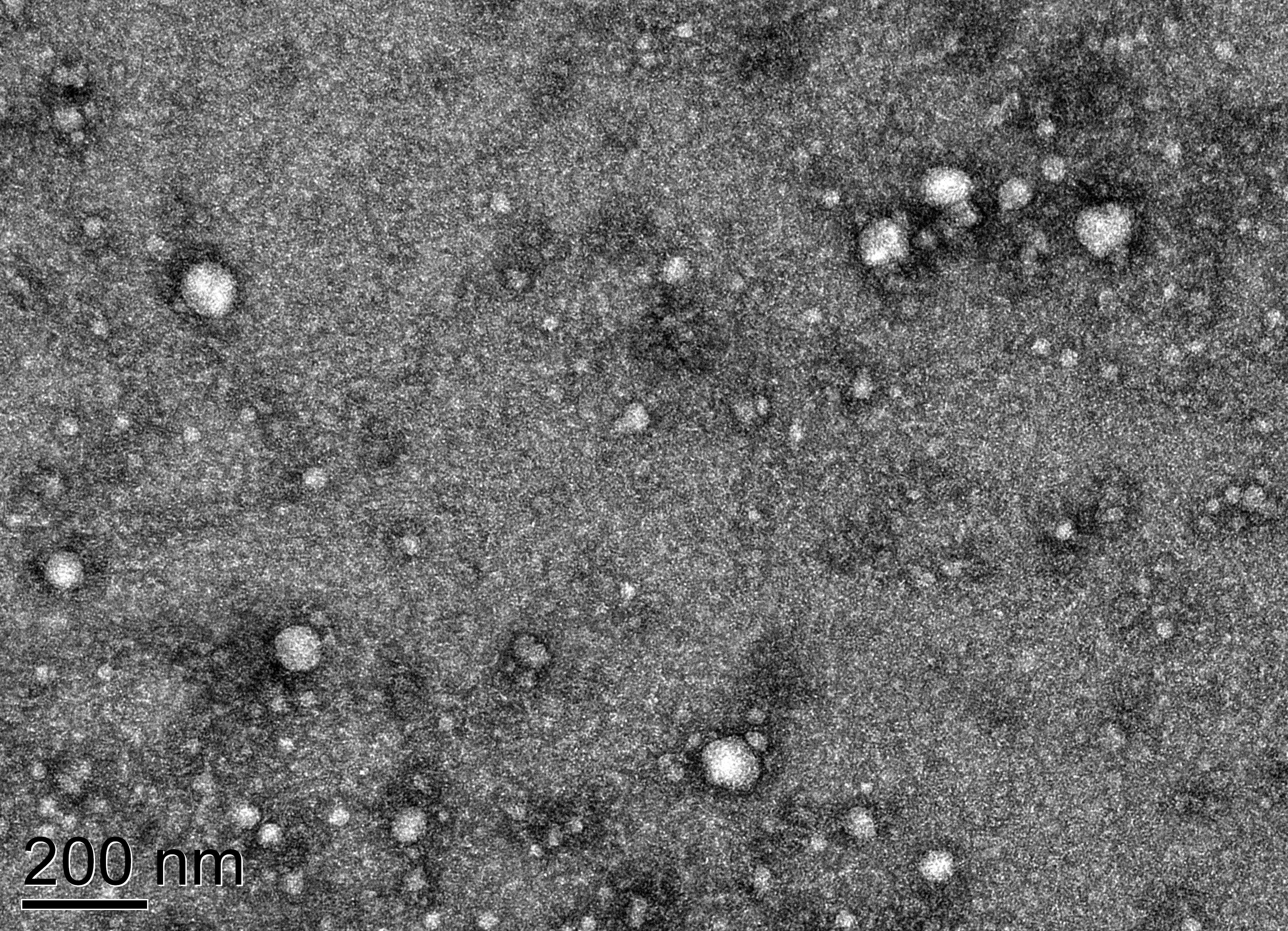

Supplement: Supplementary file 8 [file DataSheet2.ZIP › original data FIG3/Fig3A Exosome electron microscopy/2/B201.jpg]

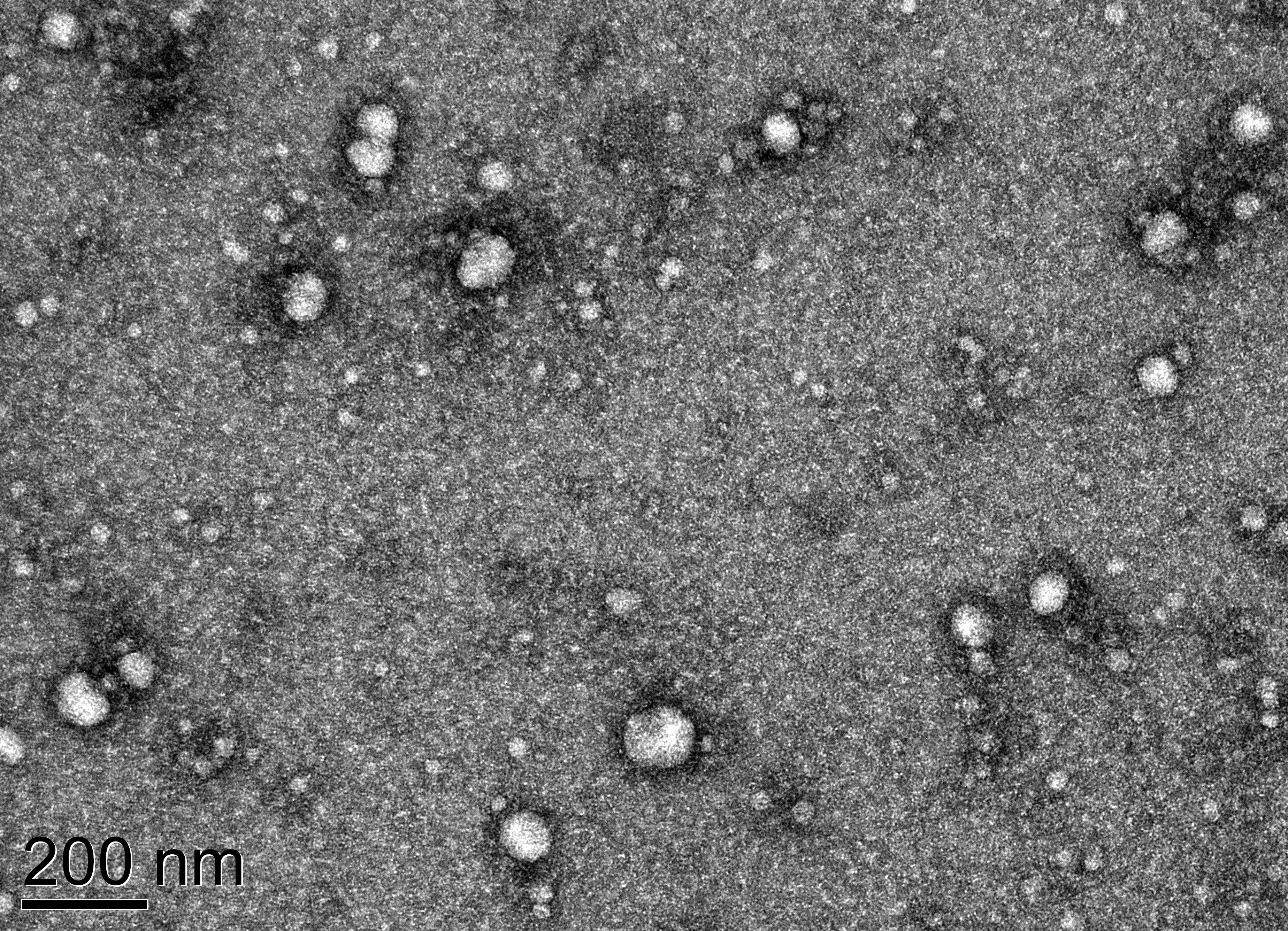

Supplement: Supplementary file 8 [file DataSheet2.ZIP › original data FIG3/Fig3A Exosome electron microscopy/2/B202.jpg]

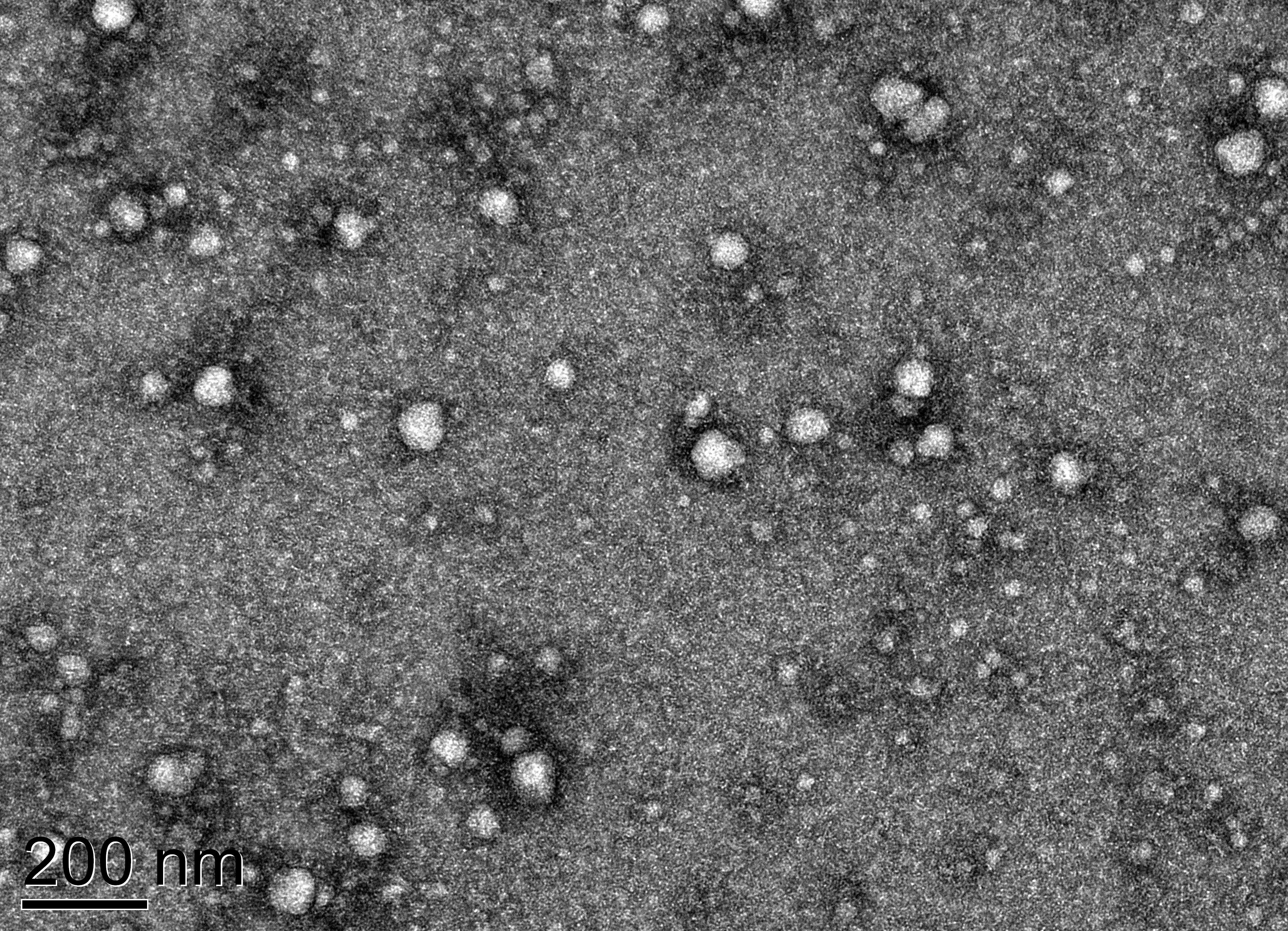

Supplement: Supplementary file 8 [file DataSheet2.ZIP › original data FIG3/Fig3A Exosome electron microscopy/2/B203.jpg]

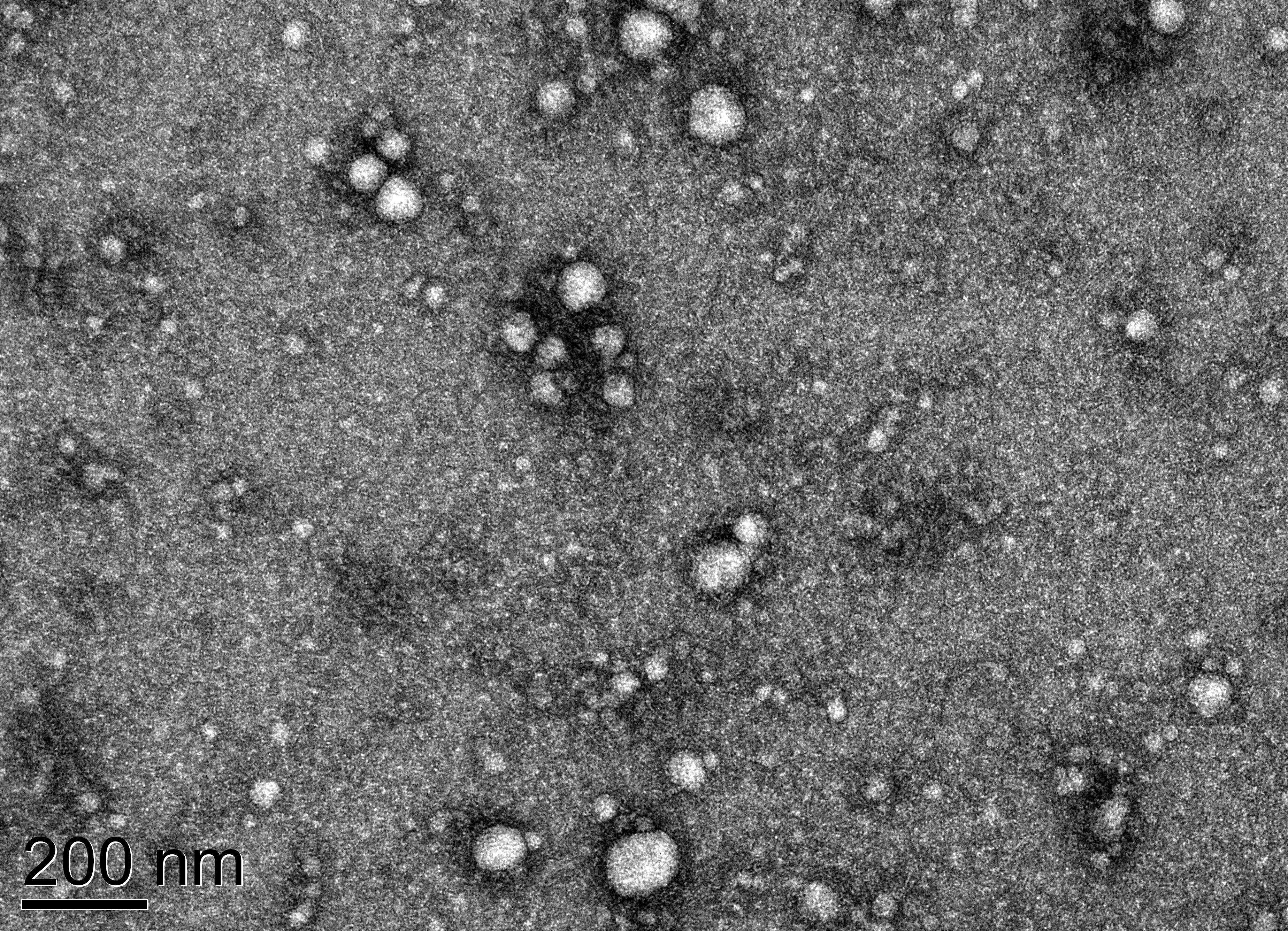

Supplement: Supplementary file 8 [file DataSheet2.ZIP › original data FIG3/Fig3A Exosome electron microscopy/3/B301.jpg]

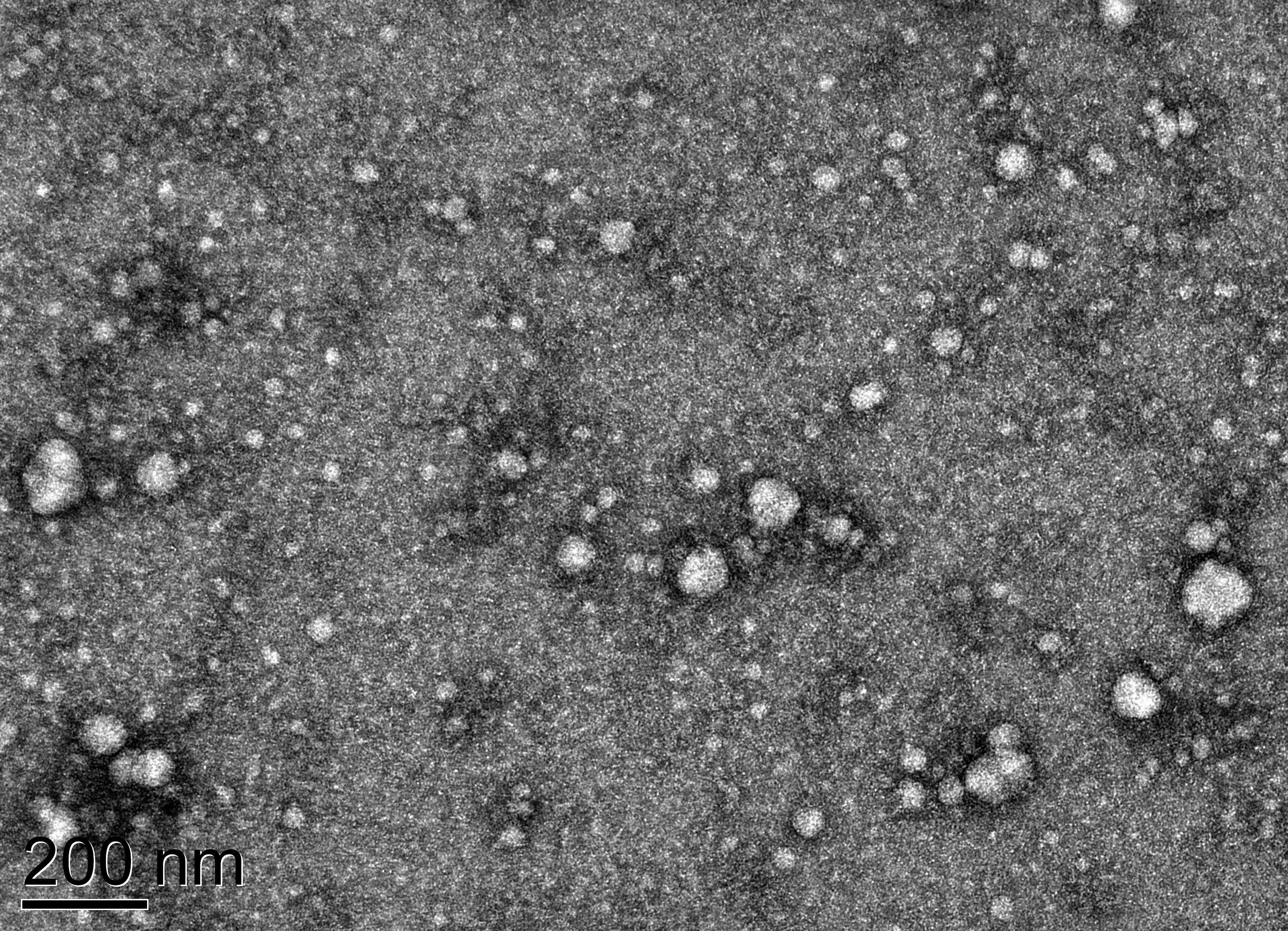

Supplement: Supplementary file 8 [file DataSheet2.ZIP › original data FIG3/Fig3A Exosome electron microscopy/3/B302.jpg]

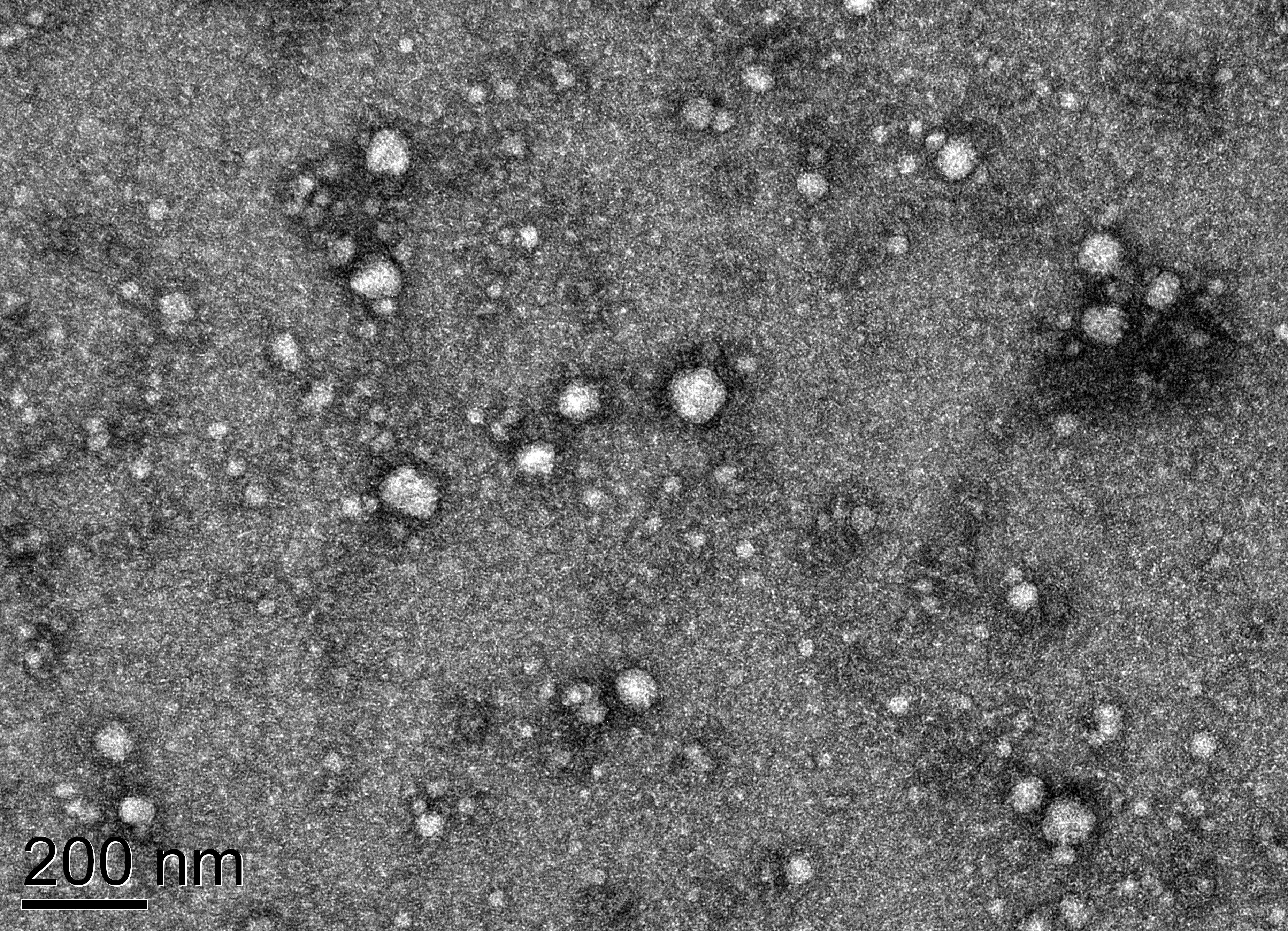

Supplement: Supplementary file 8 [file DataSheet2.ZIP › original data FIG3/Fig3A Exosome electron microscopy/3/B303.jpg]

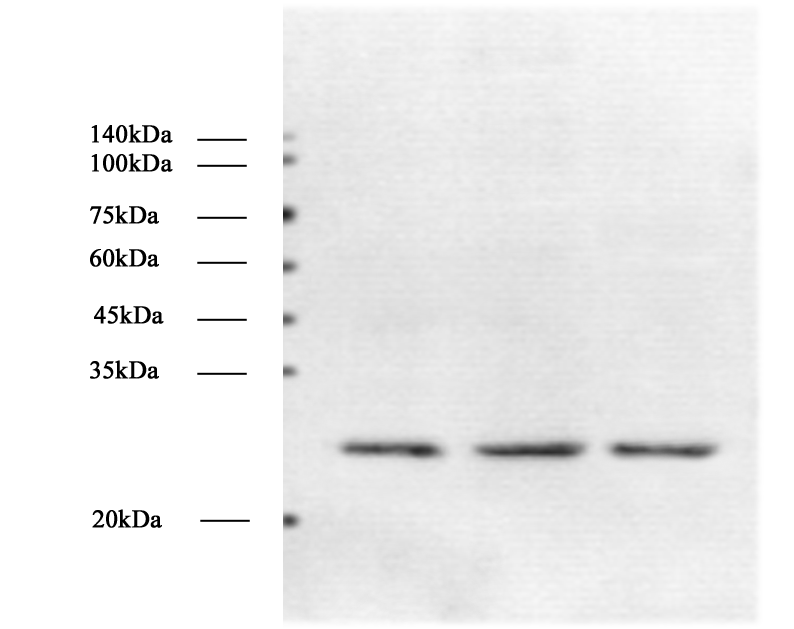

Supplement: Supplementary file 8 [file DataSheet2.ZIP › original data FIG3/Fig3B Exosomes-WB/CD63-marker.tif]

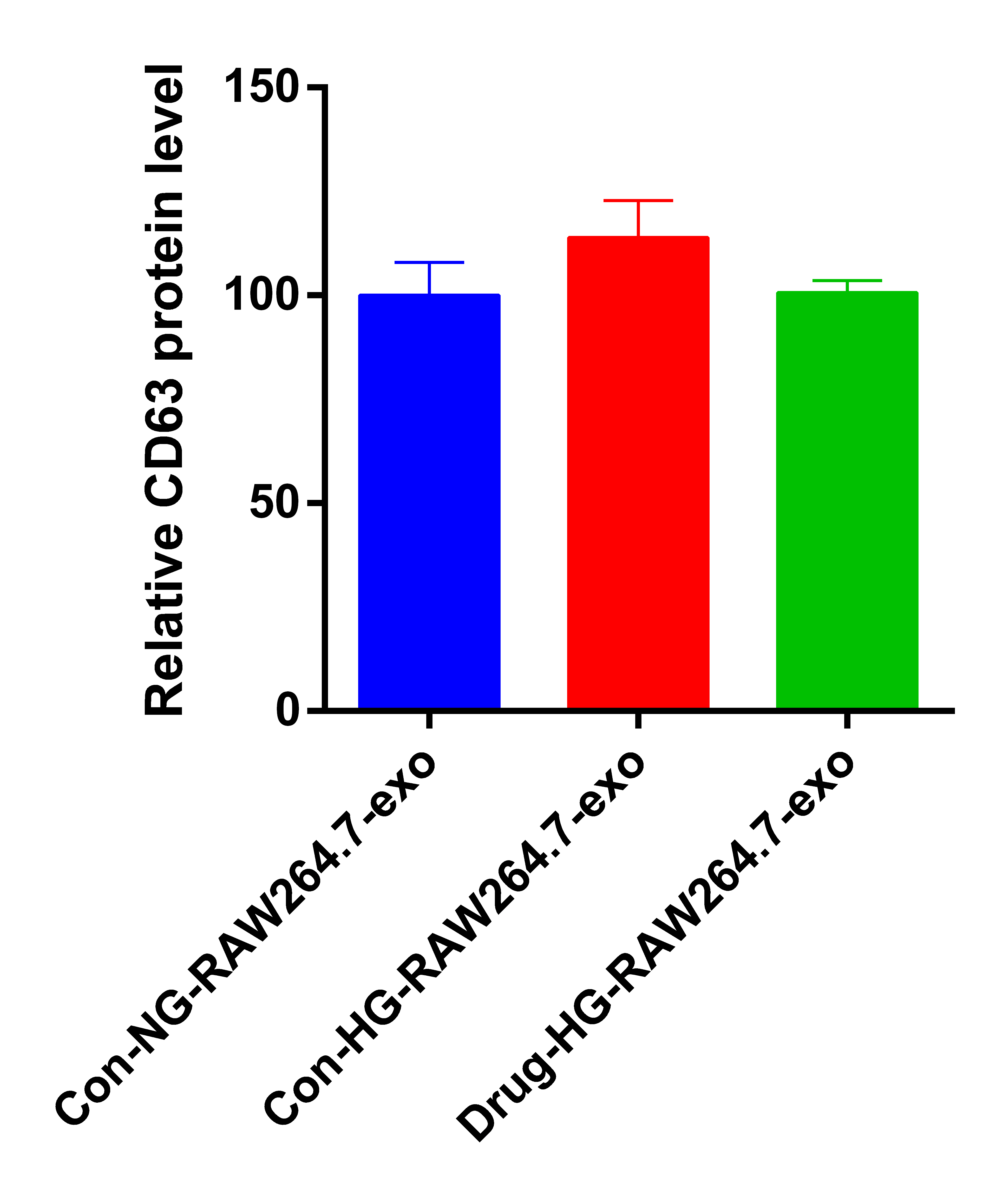

Supplement: Supplementary file 8 [file DataSheet2.ZIP › original data FIG3/Fig3B Exosomes-WB/CD63.jpg]

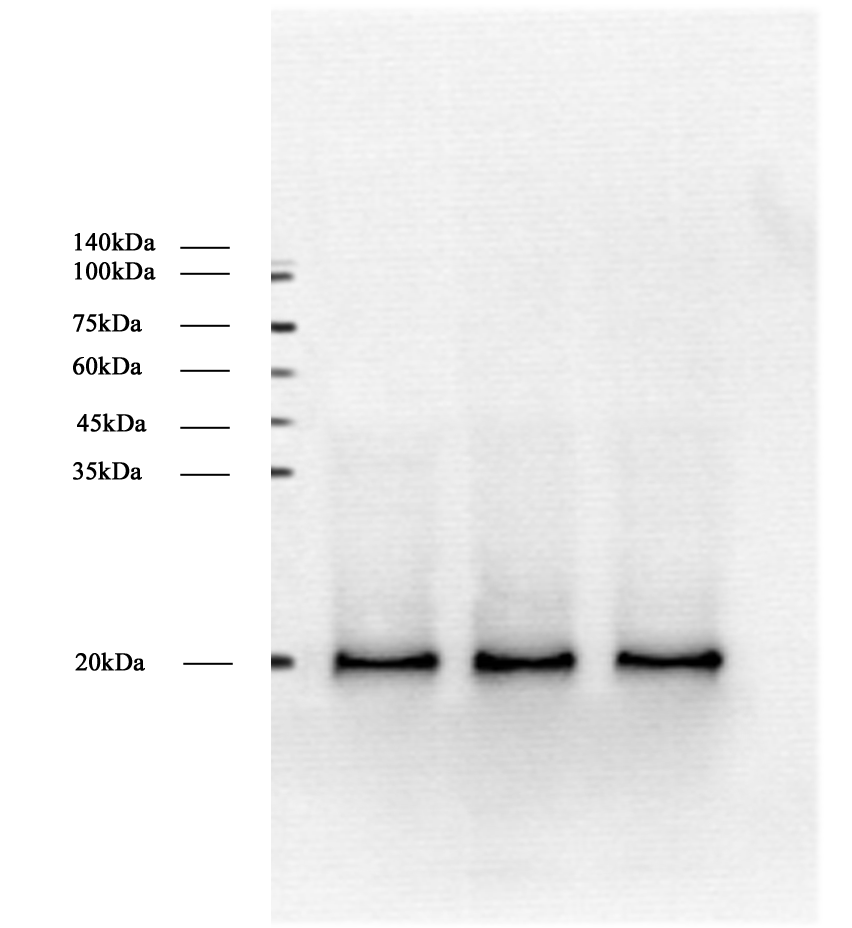

Supplement: Supplementary file 8 [file DataSheet2.ZIP › original data FIG3/Fig3B Exosomes-WB/CD81-marker.tif]

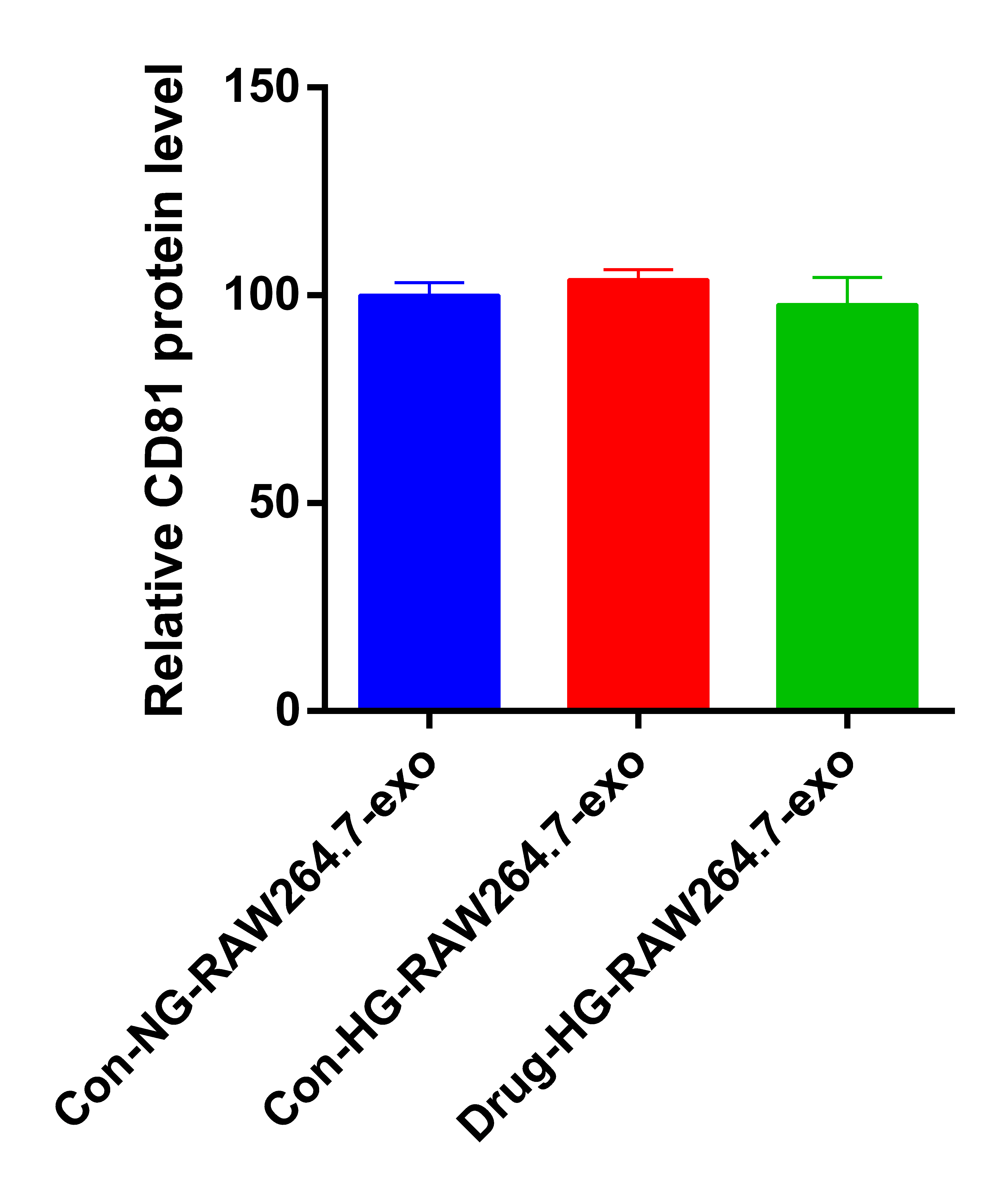

Supplement: Supplementary file 8 [file DataSheet2.ZIP › original data FIG3/Fig3B Exosomes-WB/CD81.jpg]

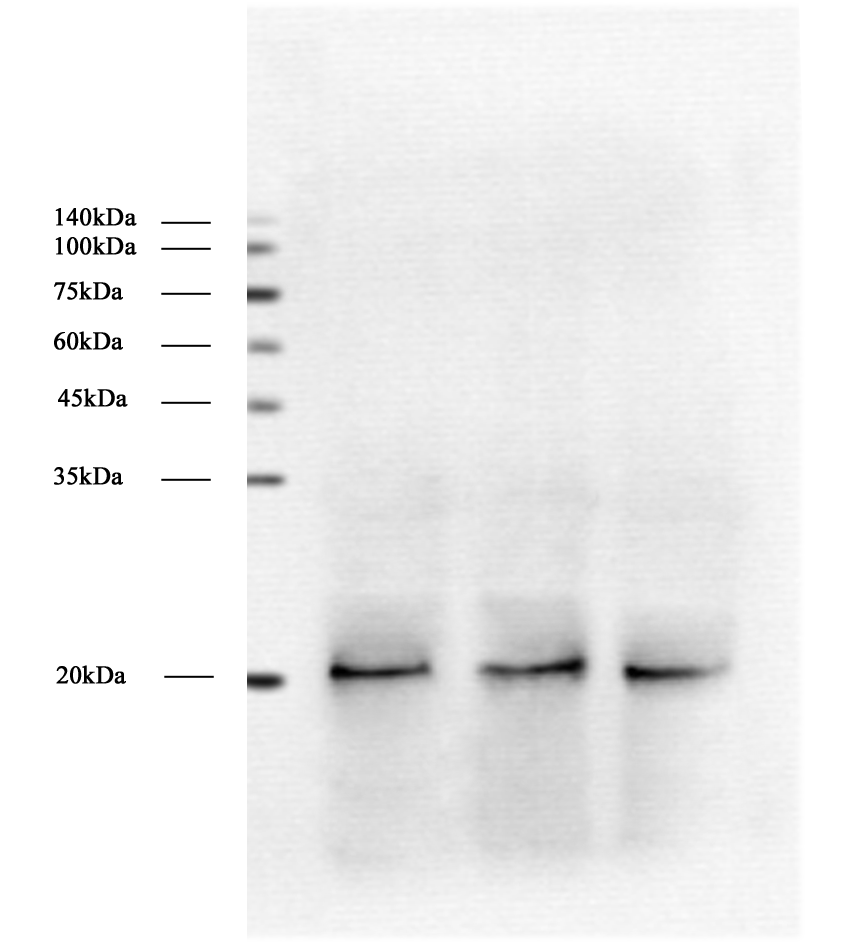

Supplement: Supplementary file 8 [file DataSheet2.ZIP › original data FIG3/Fig3B Exosomes-WB/CD9-marker.tif]

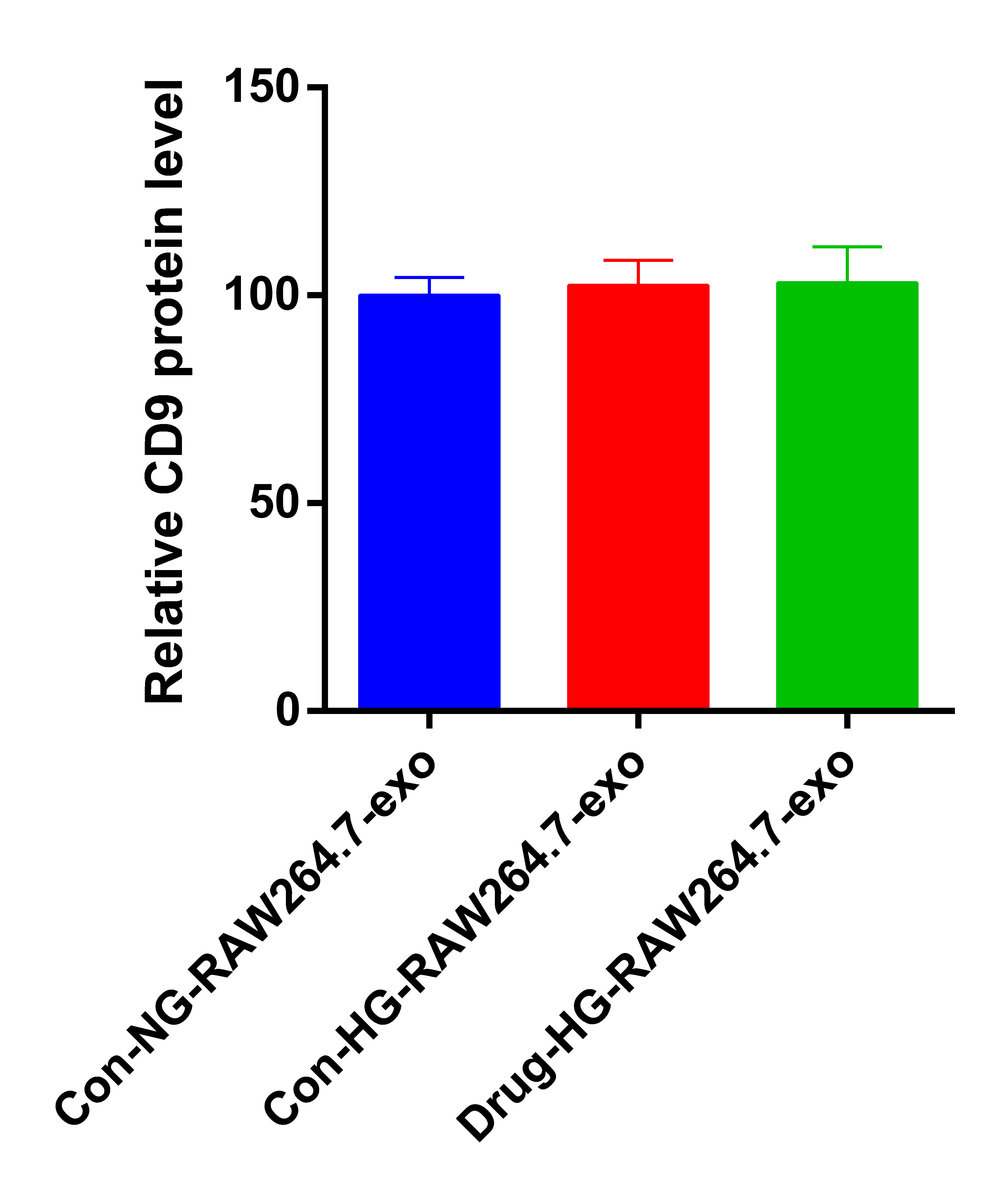

Supplement: Supplementary file 8 [file DataSheet2.ZIP › original data FIG3/Fig3B Exosomes-WB/CD9.jpg]

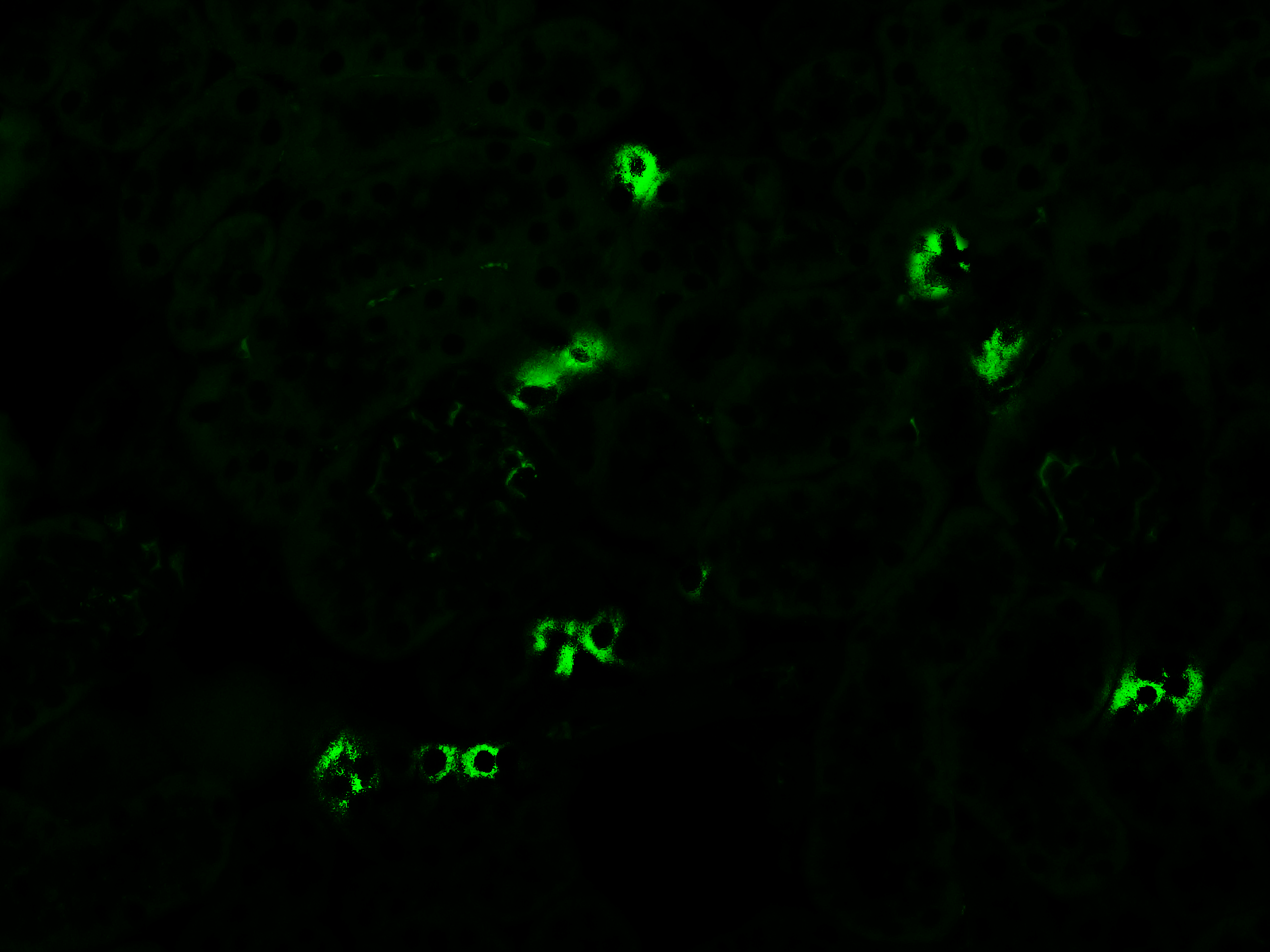

Supplement: Supplementary file 9 [file DataSheet5.ZIP › original data FIG8(II)/HLA-DR-2/2-1(400倍).tif]

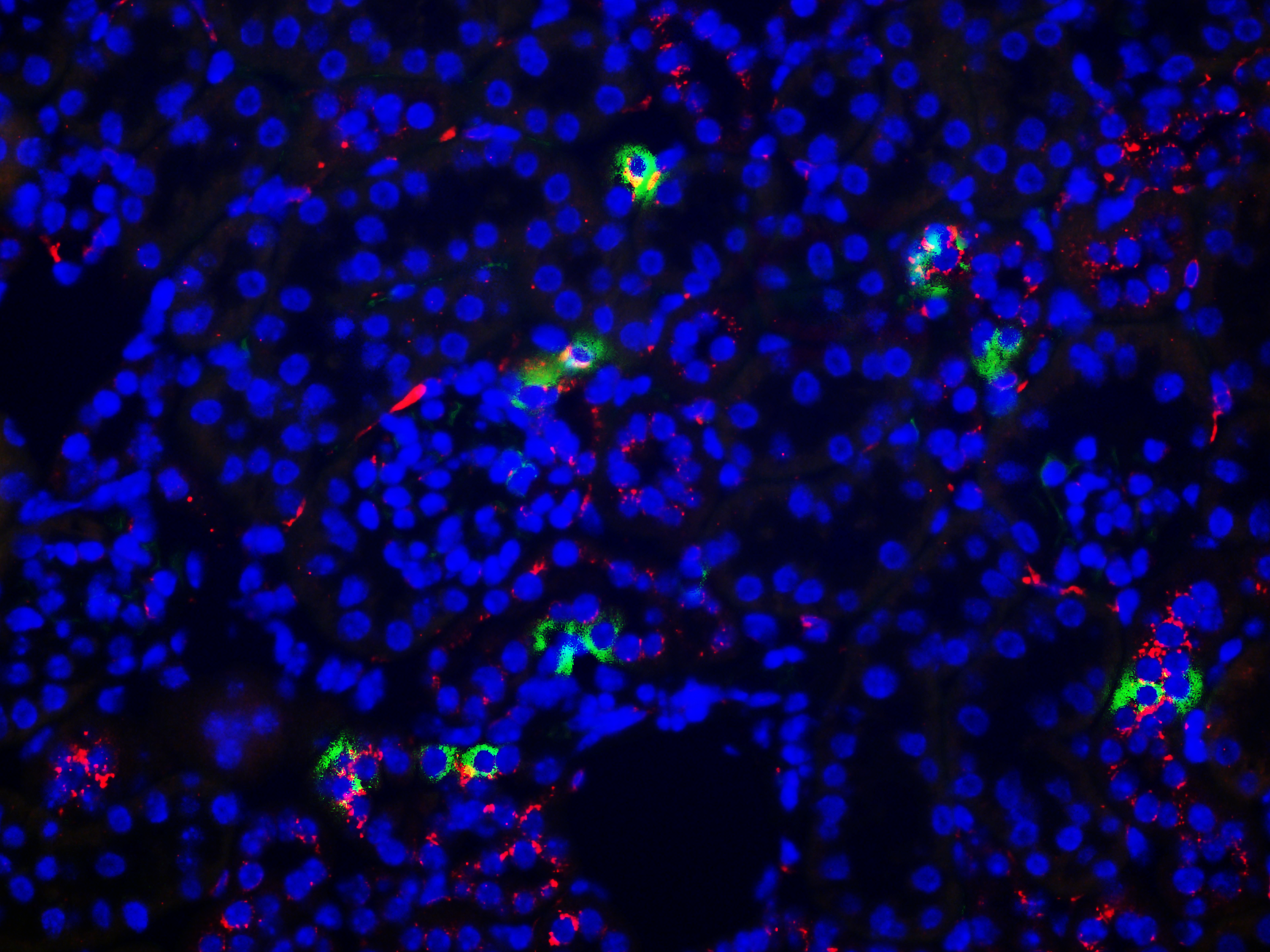

Supplement: Supplementary file 9 [file DataSheet5.ZIP › original data FIG8(II)/HLA-DR-2/2-2(400倍)-merge.tif]

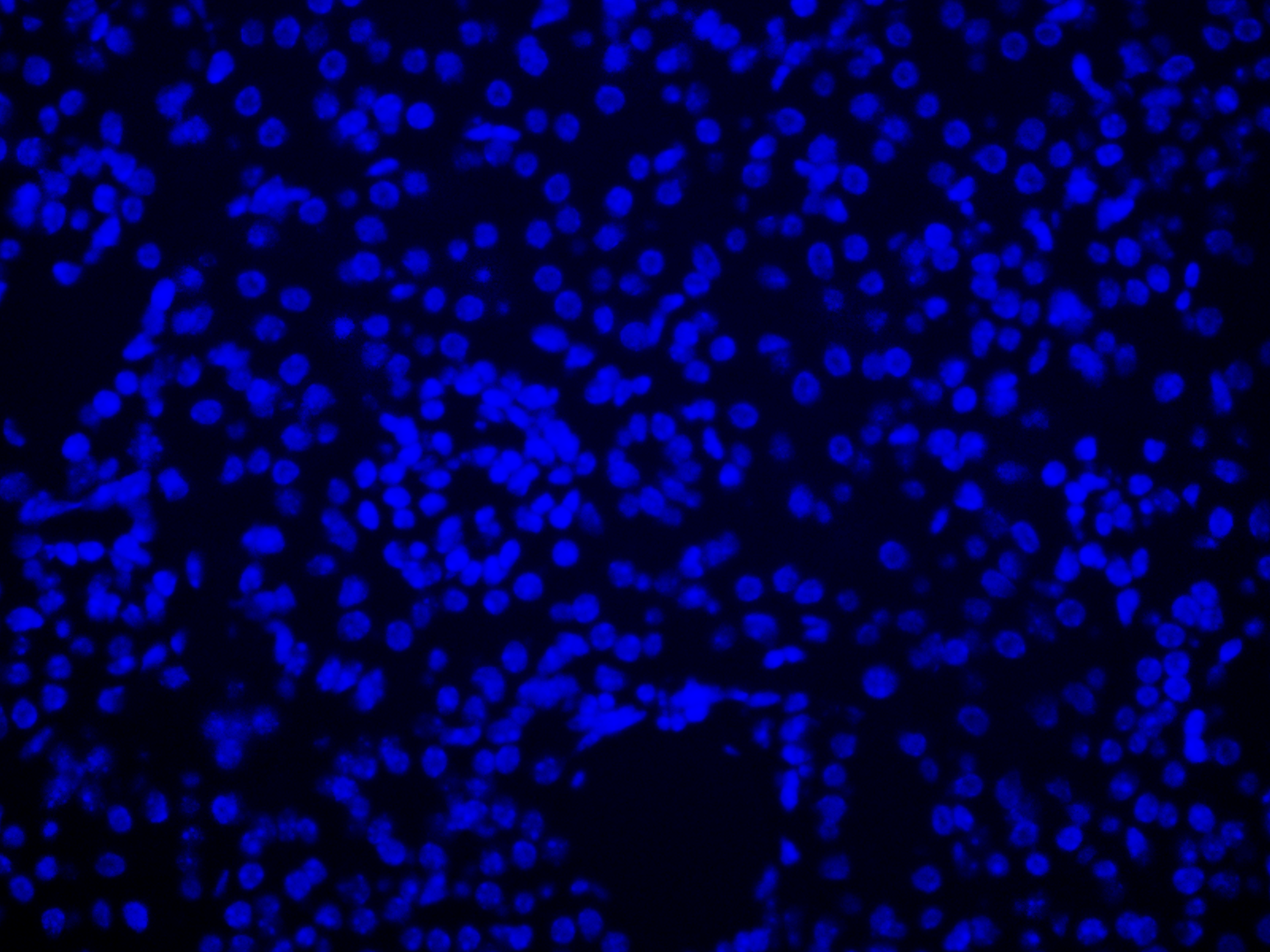

Supplement: Supplementary file 9 [file DataSheet5.ZIP › original data FIG8(II)/HLA-DR-2/2-2(400倍).tif]

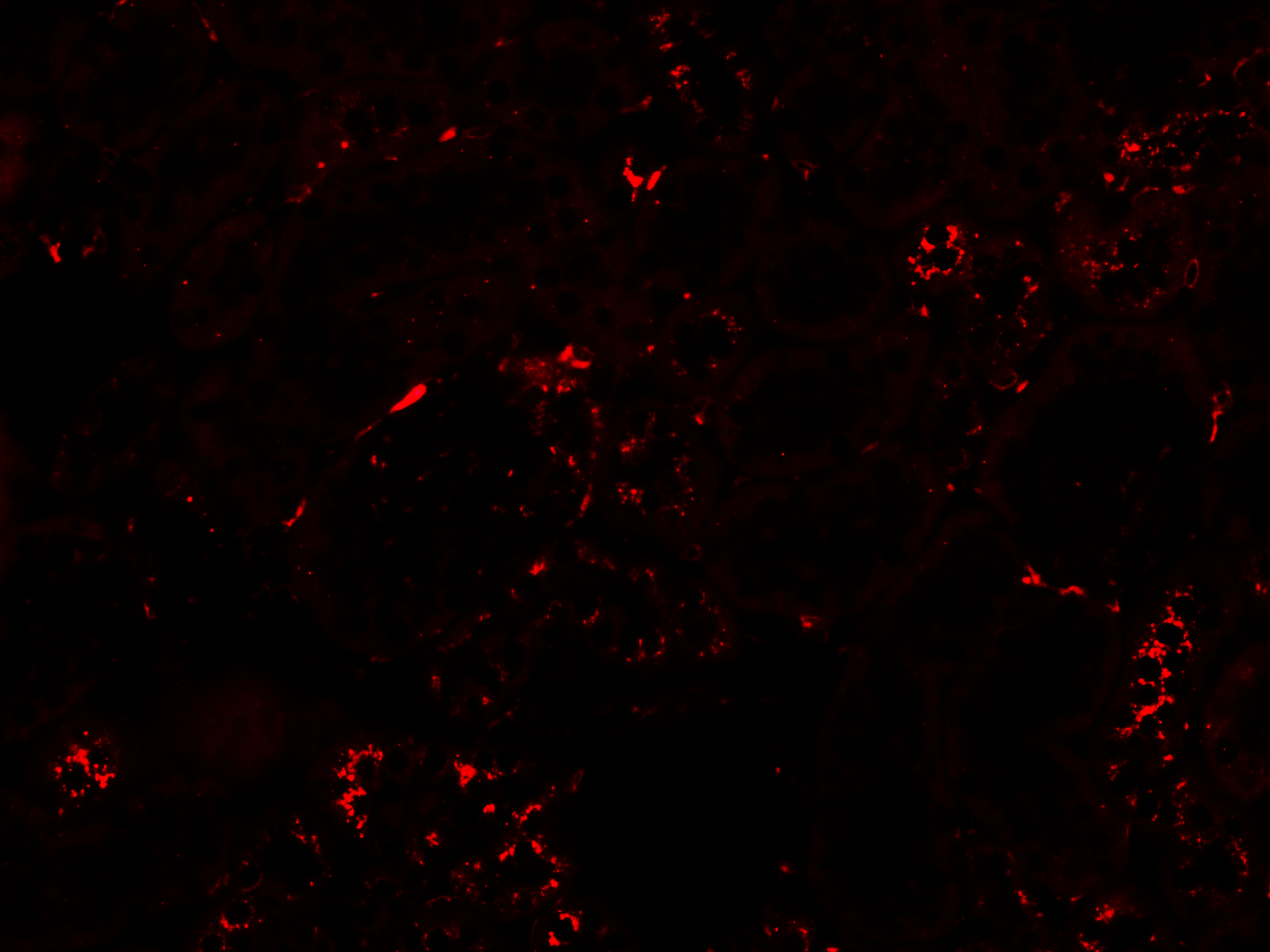

Supplement: Supplementary file 9 [file DataSheet5.ZIP › original data FIG8(II)/HLA-DR-2/2-3(400倍).tif]

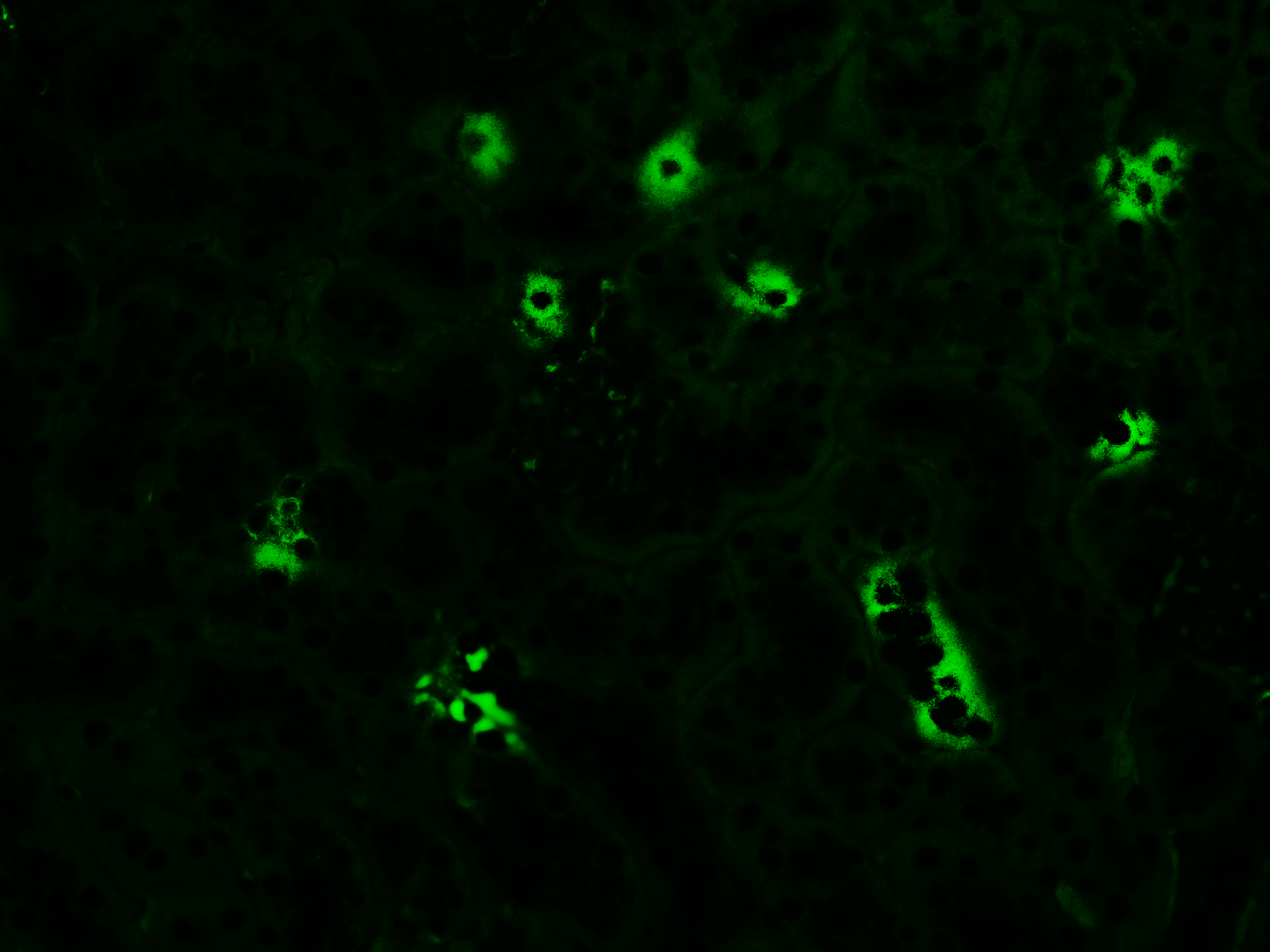

Supplement: Supplementary file 9 [file DataSheet5.ZIP › original data FIG8(II)/HLA-DR-2/2-4(400倍).tif]

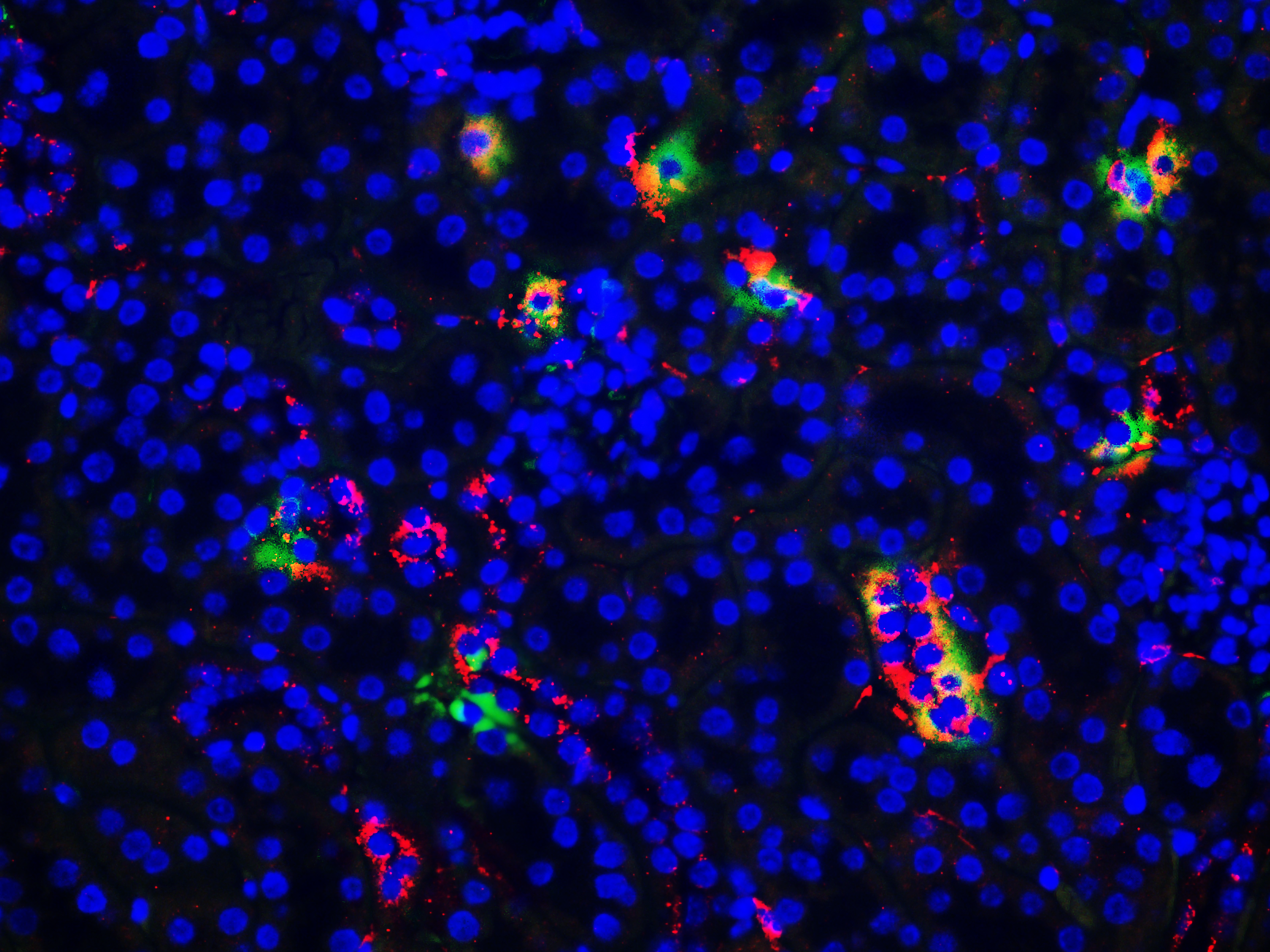

Supplement: Supplementary file 9 [file DataSheet5.ZIP › original data FIG8(II)/HLA-DR-2/2-5(400倍)-merge.tif]

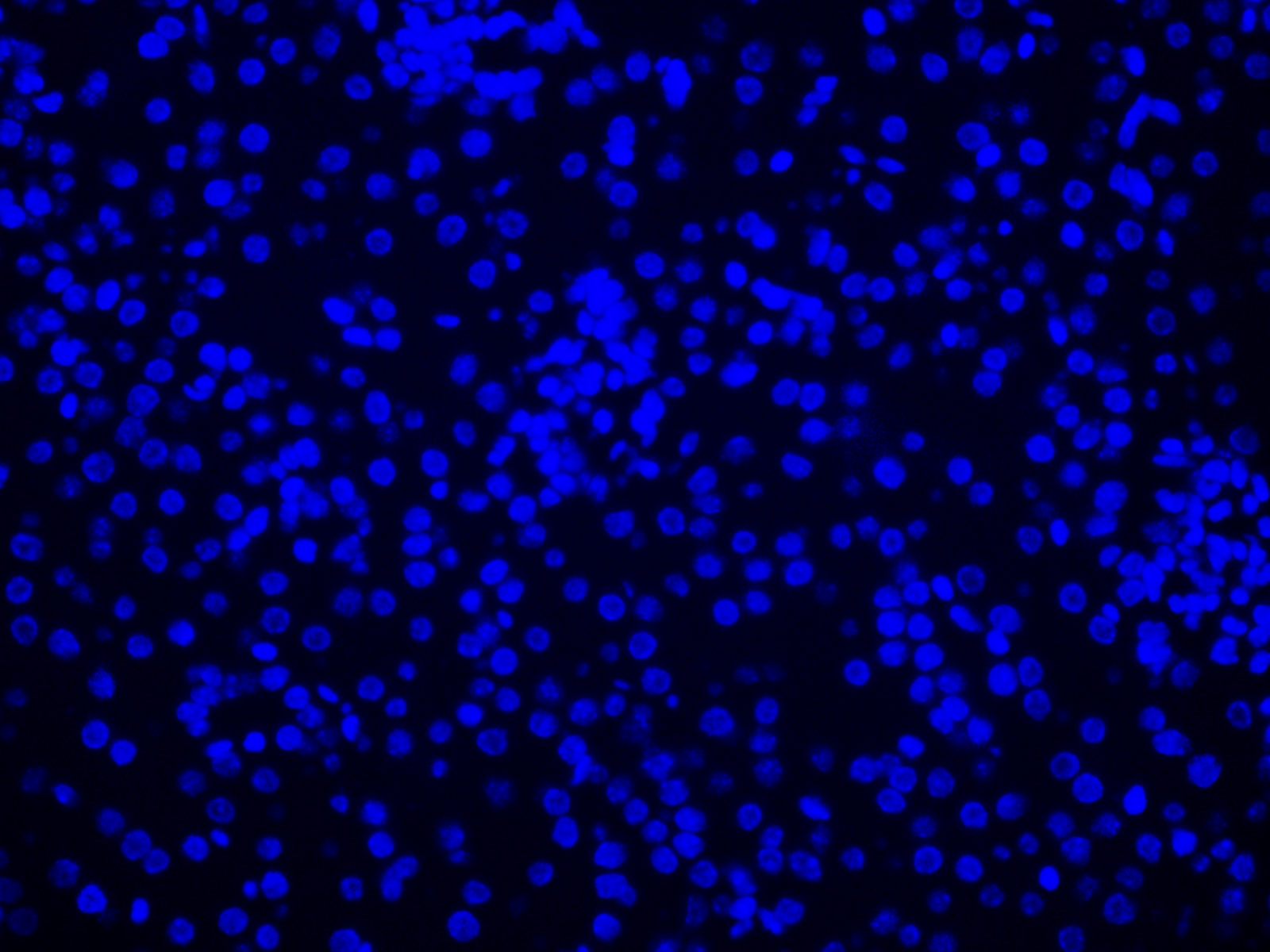

Supplement: Supplementary file 9 [file DataSheet5.ZIP › original data FIG8(II)/HLA-DR-2/2-5(400倍).tif]

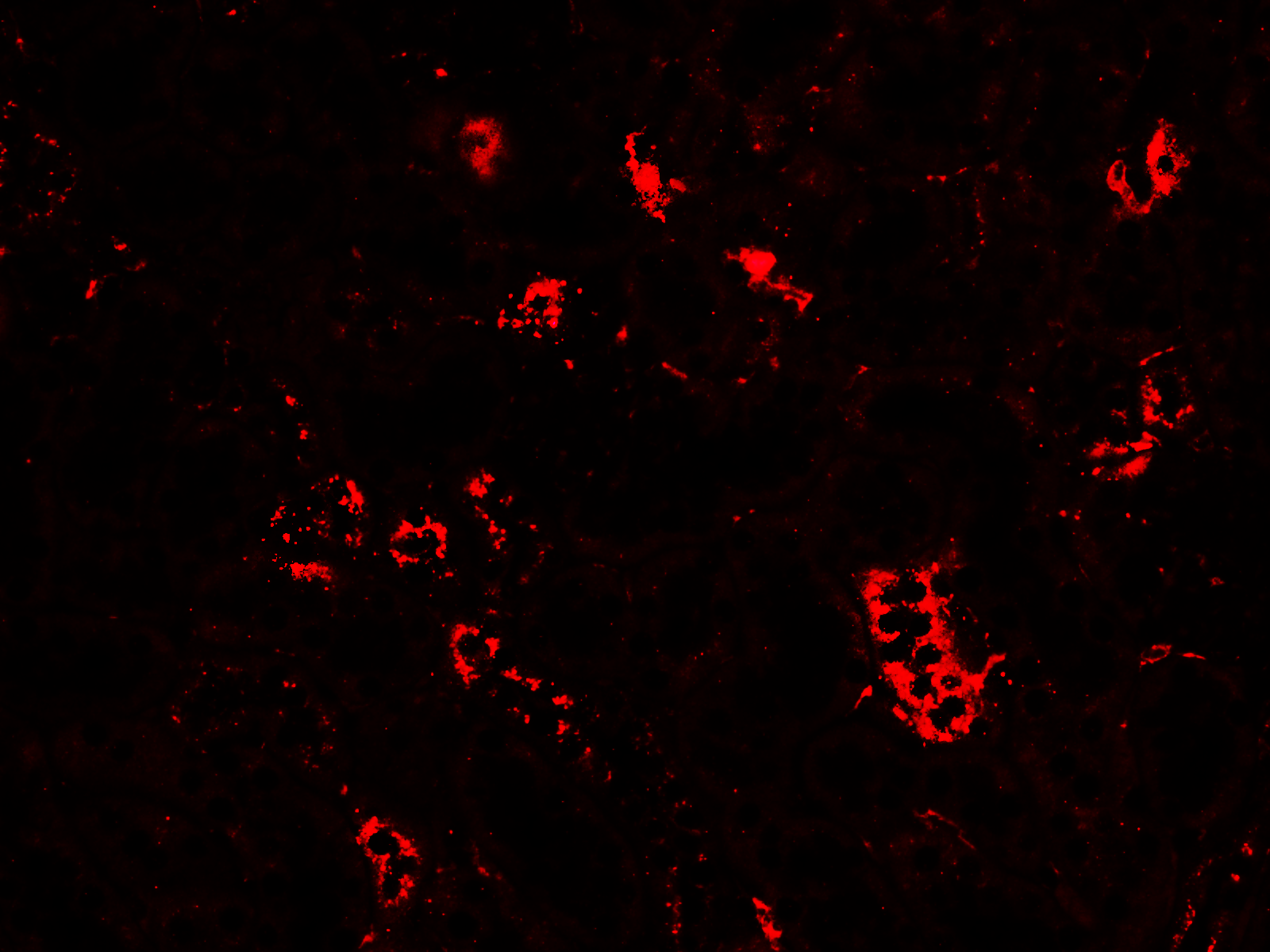

Supplement: Supplementary file 9 [file DataSheet5.ZIP › original data FIG8(II)/HLA-DR-2/2-6(400倍).tif]

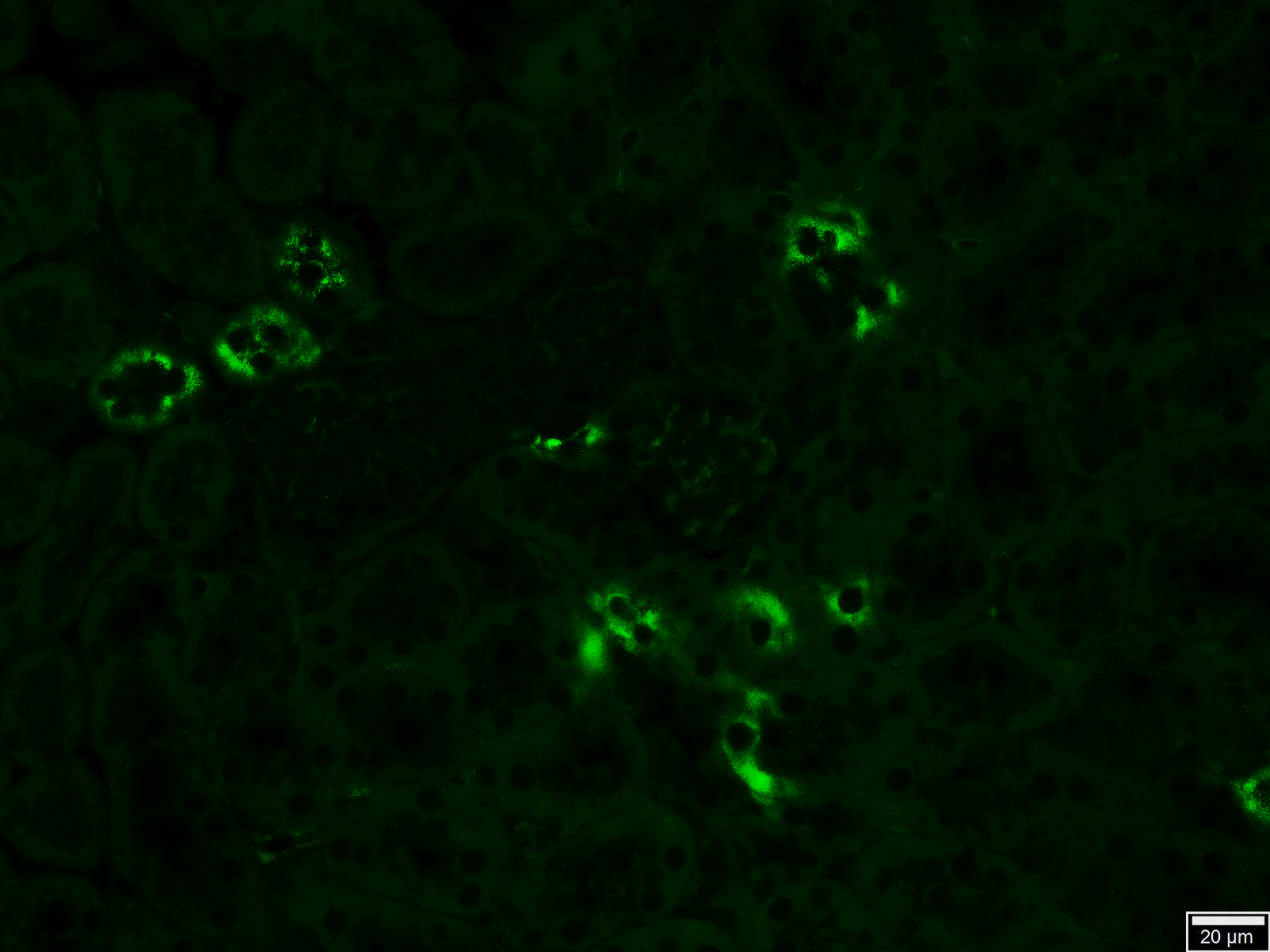

Supplement: Supplementary file 9 [file DataSheet5.ZIP › original data FIG8(II)/HLA-DR-2/2-7(400倍).tif]

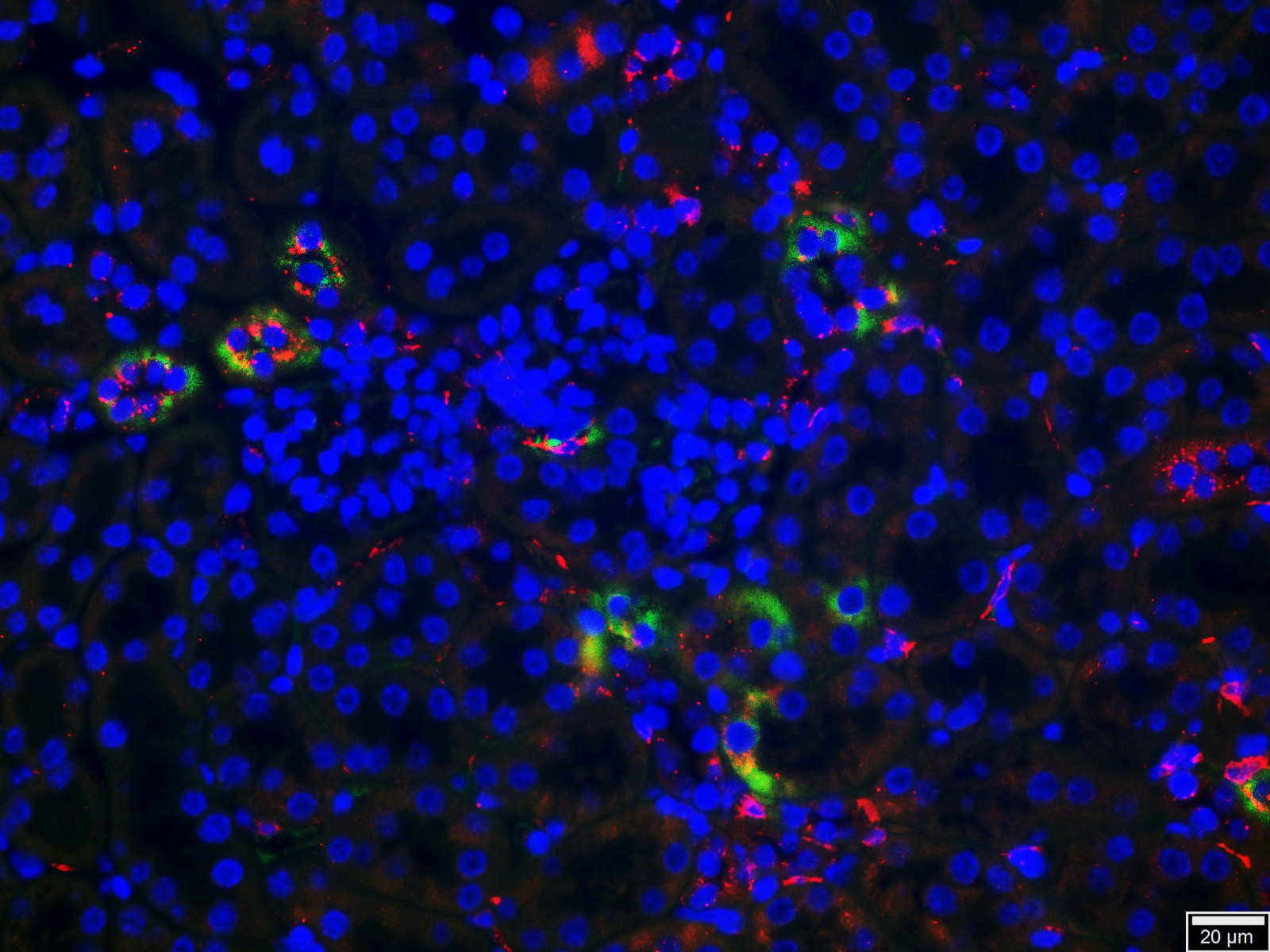

Supplement: Supplementary file 9 [file DataSheet5.ZIP › original data FIG8(II)/HLA-DR-2/2-8(400倍)-merge.tif]

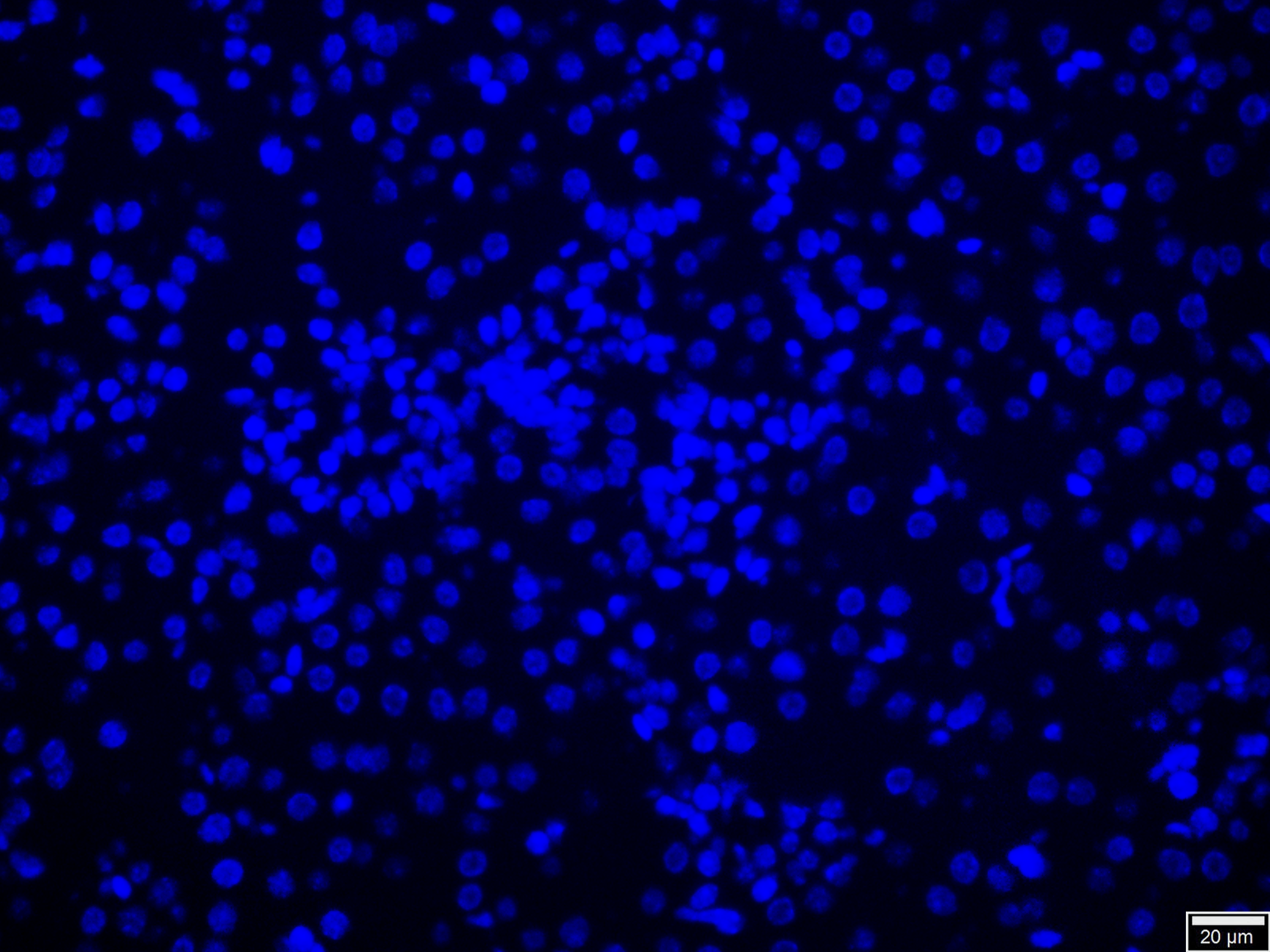

Supplement: Supplementary file 9 [file DataSheet5.ZIP › original data FIG8(II)/HLA-DR-2/2-8(400倍).tif]

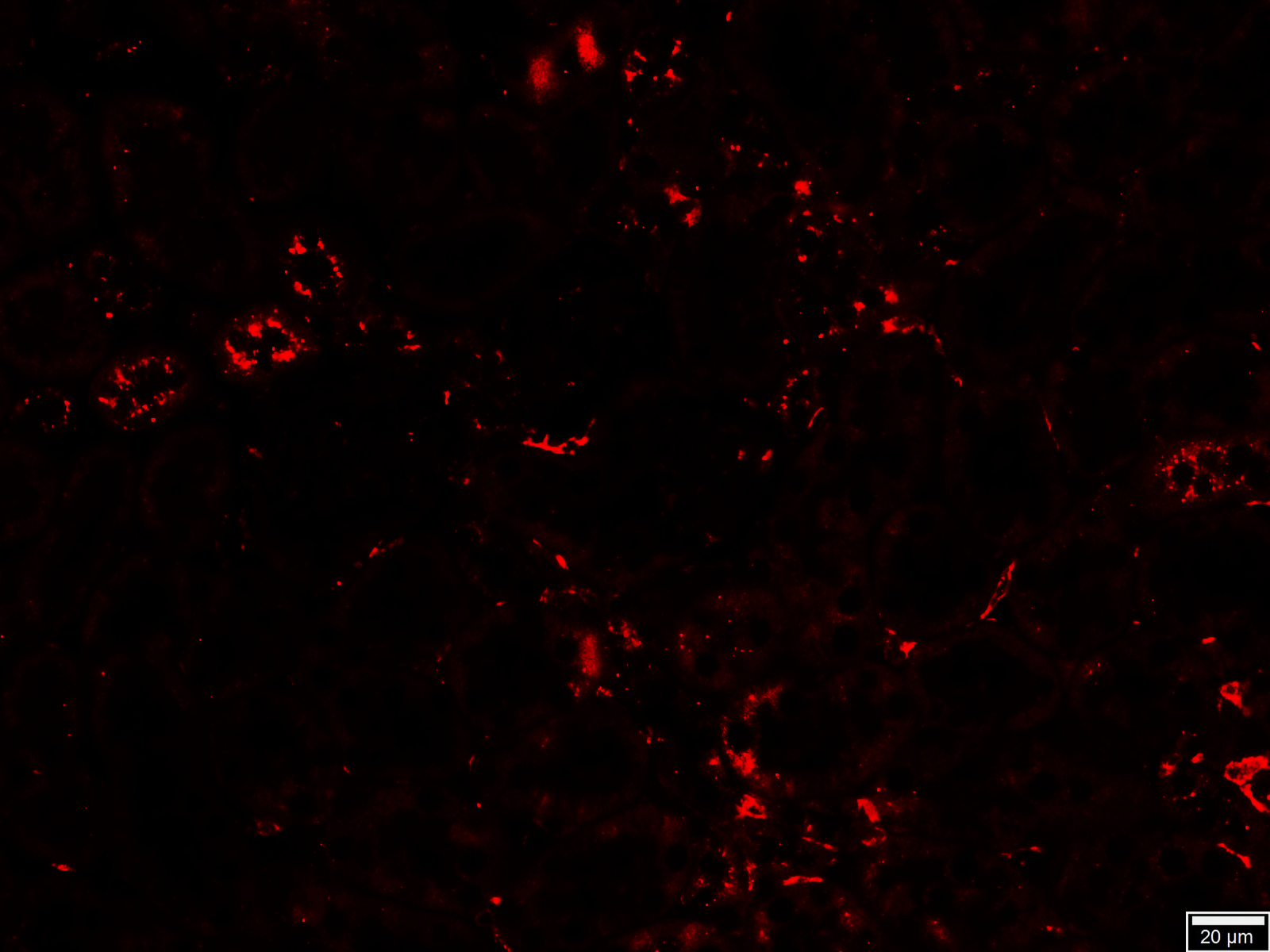

Supplement: Supplementary file 9 [file DataSheet5.ZIP › original data FIG8(II)/HLA-DR-2/2-9(400倍).tif]

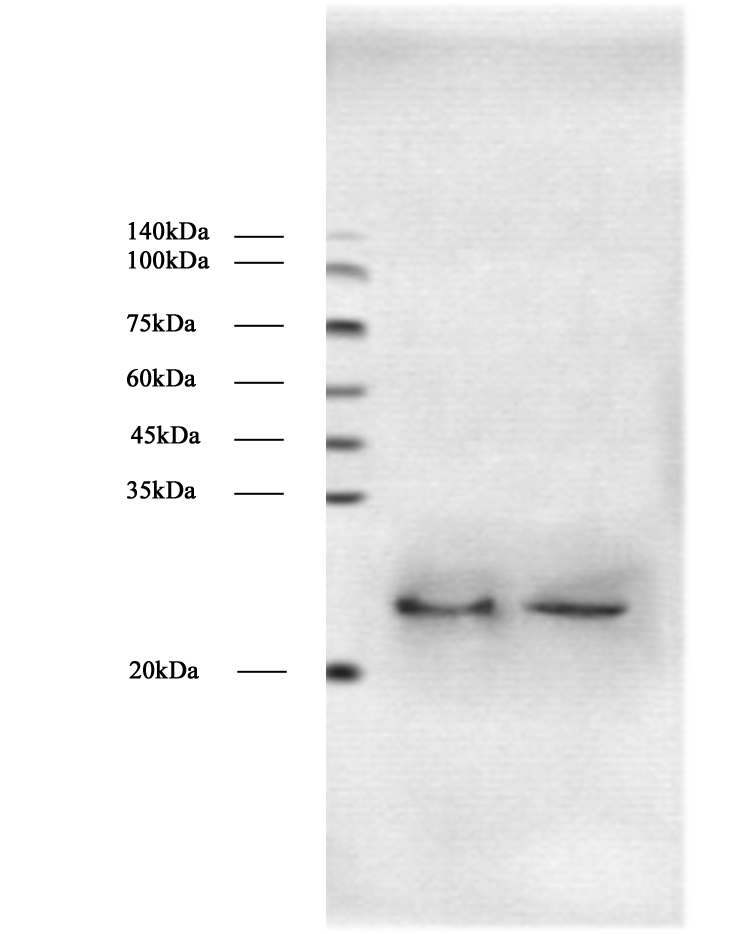

Supplement: Supplementary file 10 [file DataSheet7.ZIP › Supplemental data/Supplemental Fig1A Exosomes-WB/CD63-marker.tif]

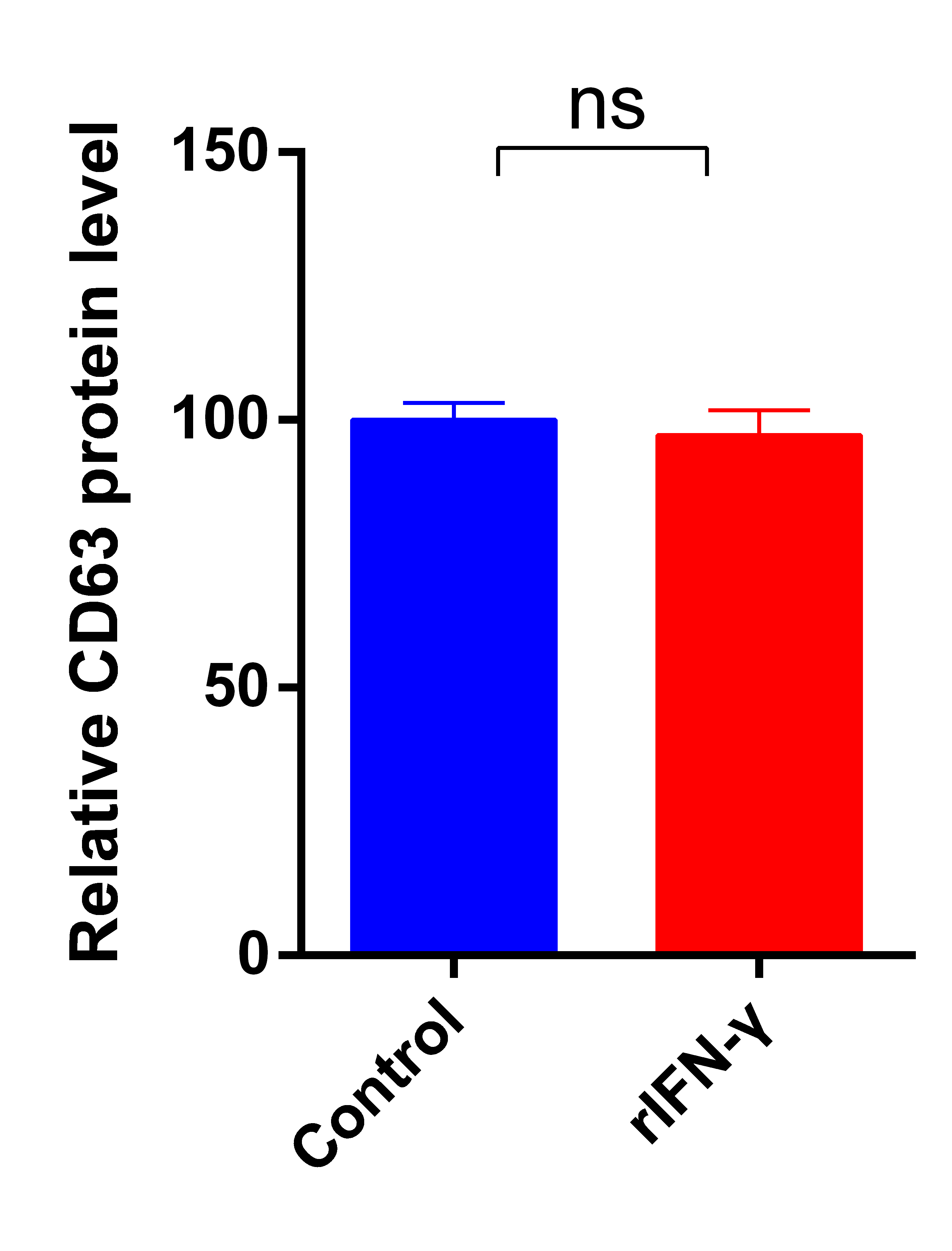

Supplement: Supplementary file 10 [file DataSheet7.ZIP › Supplemental data/Supplemental Fig1A Exosomes-WB/cd63.jpg]

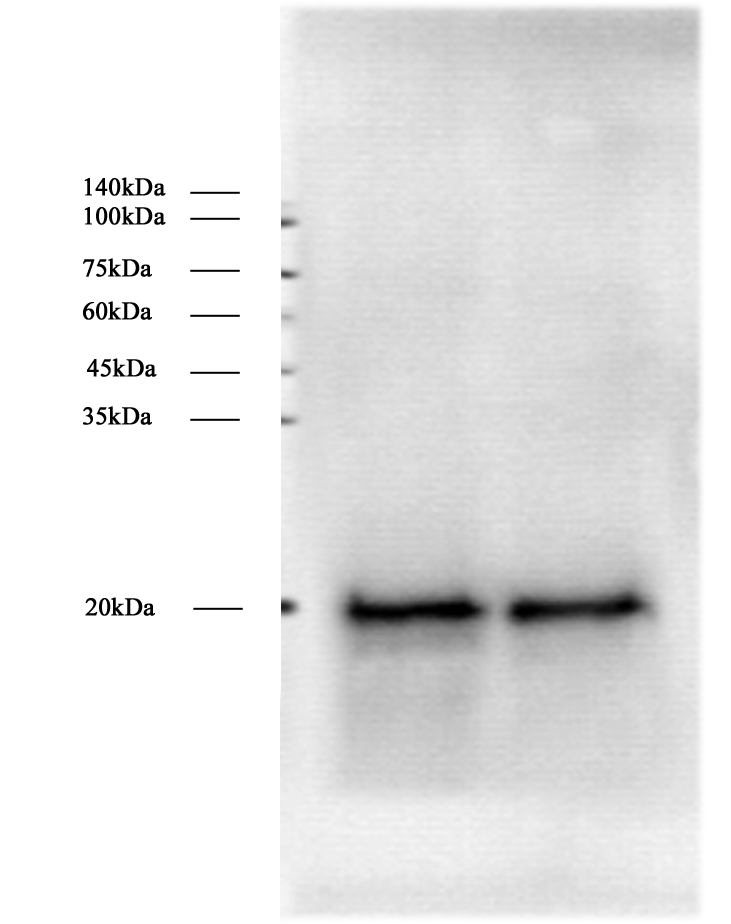

Supplement: Supplementary file 10 [file DataSheet7.ZIP › Supplemental data/Supplemental Fig1A Exosomes-WB/CD81-marker.tif]

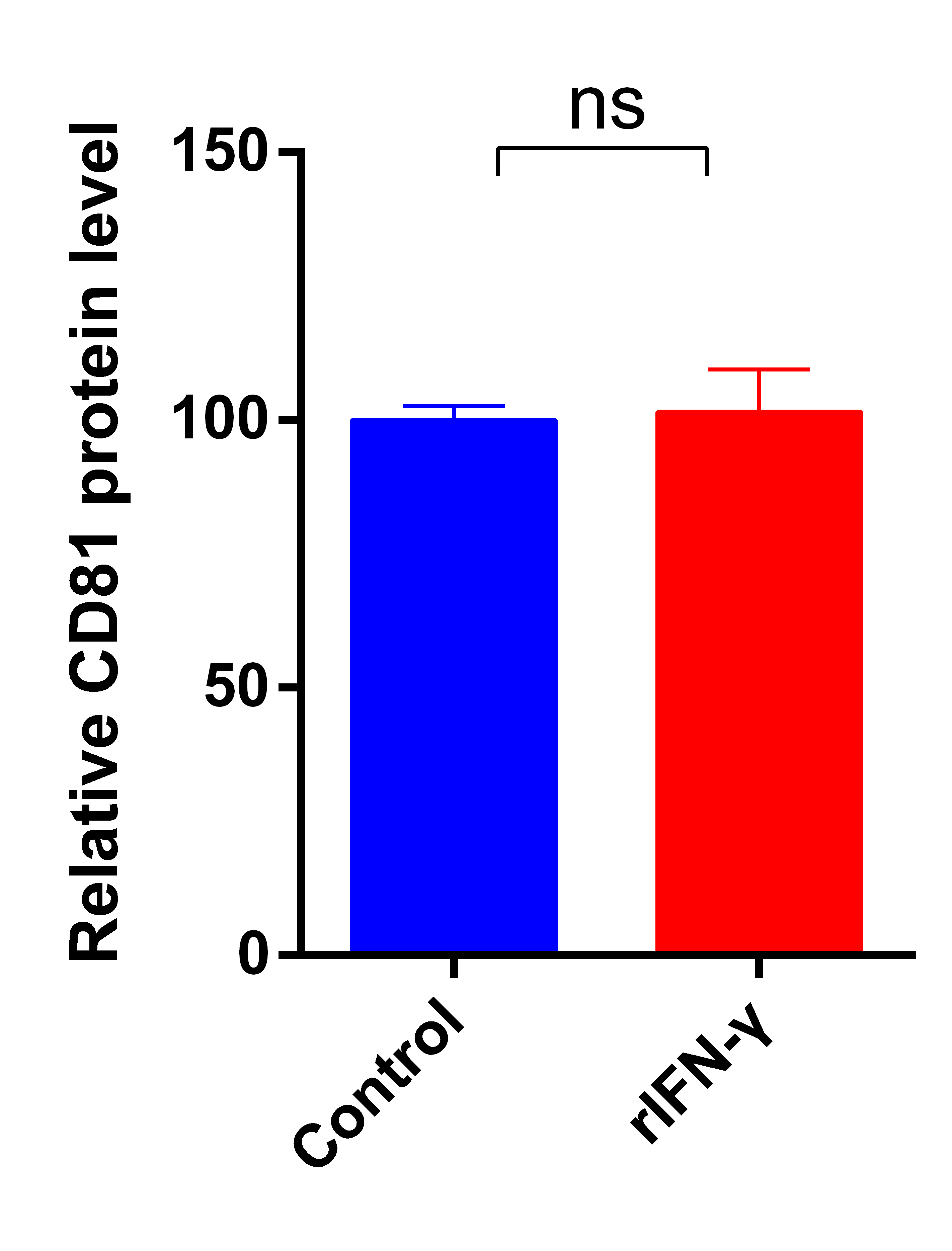

Supplement: Supplementary file 10 [file DataSheet7.ZIP › Supplemental data/Supplemental Fig1A Exosomes-WB/cd81.jpg]

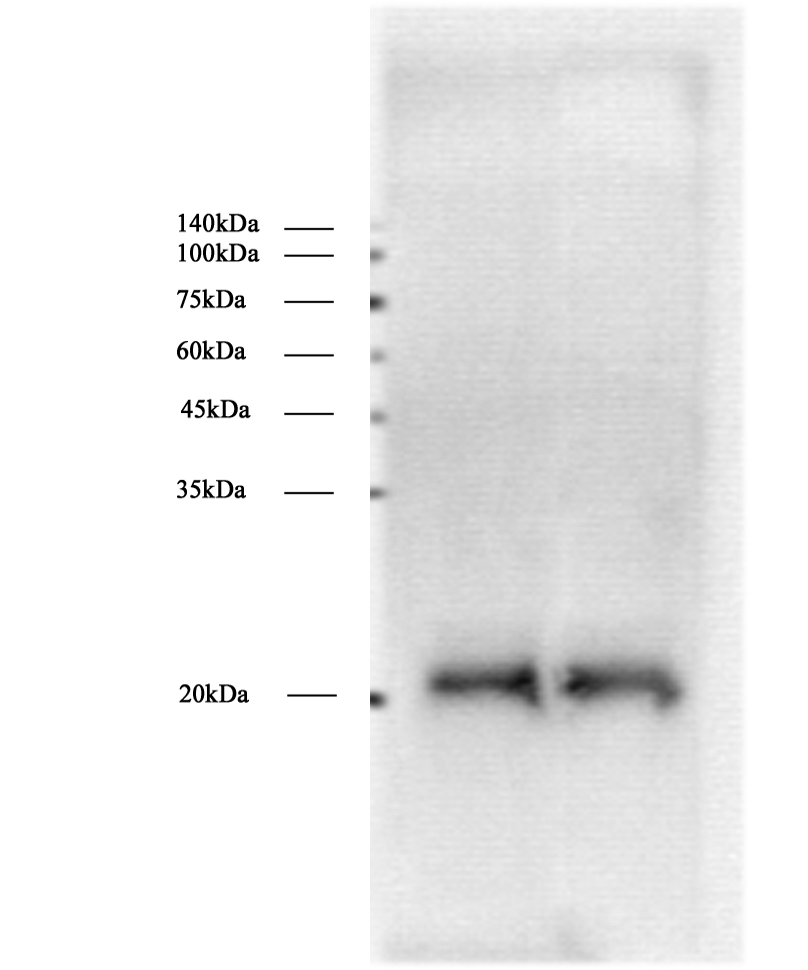

Supplement: Supplementary file 10 [file DataSheet7.ZIP › Supplemental data/Supplemental Fig1A Exosomes-WB/CD9-marker.tif]

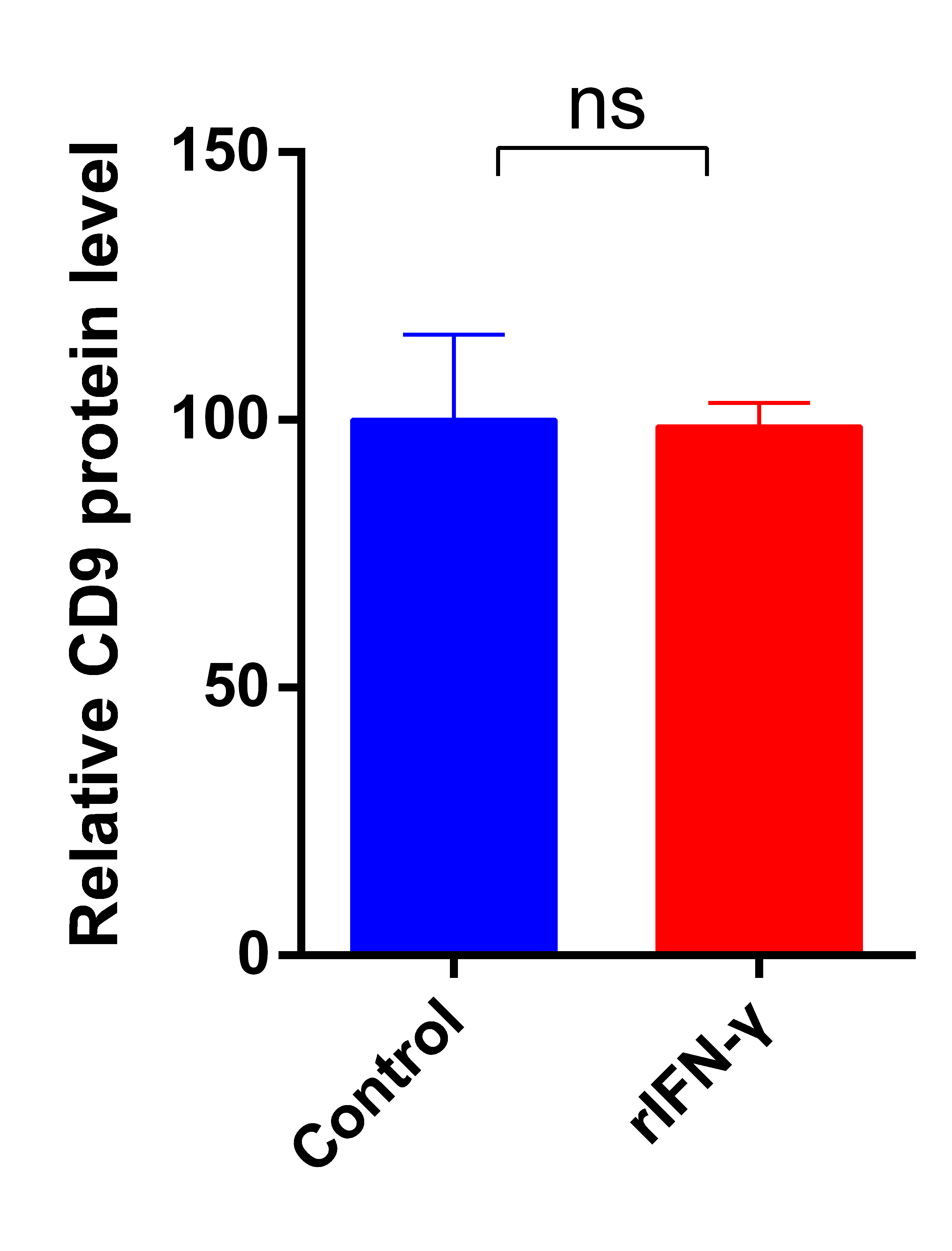

Supplement: Supplementary file 10 [file DataSheet7.ZIP › Supplemental data/Supplemental Fig1A Exosomes-WB/cd9.jpg]
